# [B(O_2_C_2_(CF_3_)_4_)_2_]^−^ ([FPB]^−^): Repurposing This Weakly Coordinating Anion for Solid-State Molecular Organometallic (SMOM) Chemistry

**DOI:** 10.1021/acs.organomet.5c00112

**Published:** 2025-05-04

**Authors:** Kristof M. Altus, M. Arif Sajjad, Stuart A. Macgregor, Andrew S. Weller

**Affiliations:** † Department of Chemistry, 8748 University of York, York YO10 5DD, U.K.; ‡ EaStCHEM School of Chemistry, North Haugh, University of St. Andrews, St Andrews KY16 9ST, U.K.

## Abstract

The perfluoropinacol borate-based anion [B­(O_2_C_2_(CF_3_)_4_)_2_]^−^, **[FPB]** ^–^, is developed as a weakly coordinating anion for single-crystal to single-crystal organometallic solid/gas reactivity, resulting in the isolation and characterization (including periodic DFT and IGMH analysis) of the σ-alkane complex [Rh­(Cy_2_PCH_2_CH_2_PCy_2_)­(*exo*-η^2^η^2^-norbornane)]­[FPB]. The synthetically useful solvent-free Na^+^ salt, Na­[FPB], and oxonium acid [H­(OEt_2_)_2_]­[FPB] are also reported.

## Graphical abstract

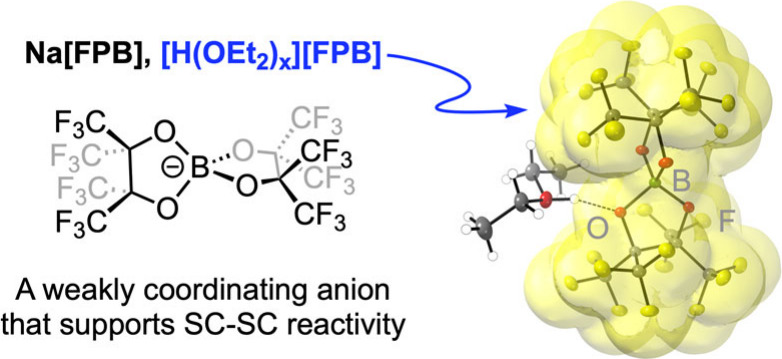

Weakly coordinating anions (WCAs) have been instrumental in the development of the synthetic and catalytic organometallic and main-group chemistry of reactive cationic species.^1–4^ The ideal WCA should be chemically robust and of low polarizability, with the negative charge delocalized over a large surface area. While there are many different WCAs,^1^ the most popular are based upon alkoxyaluminates, [Al­(OR^F^)_4_]^−^ (e.g., OR^F^ = OC­(CF_3_)_3_, OCH­(CF_3_)_2_),^5^ or arylborates, e.g., [BAr^F^_4_]^−^ (Ar^F^ = 3,5-(CF_3_)_2_C_6_H_3_),^6^ Chart 1.

**Chart 1:**
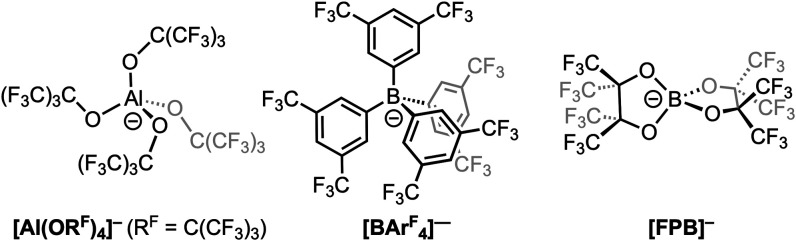
Common WCAs and the [FPB]^−^ Anion

We have used the [BAr^F^_4_]^−^ anion extensively in single-crystal to single-crystal (SC-SC) solid-state molecular organometallic chemistry (SMOM),^7–12^ where partnering with a reactive transition metal cation allows for the isolation of solution-unstable complexes by solid/gas reactivity. For example, the stable σ-alkane complex [Rh­(Cy_2_PCH_2_CH_2_PCy_2_)­(*endo*-η^2^η^2^-NBA)]­[BAr^F^_4_] **[1-NBA]­[BAr** ^**F**^_**4**_ **]** (NBA = norbornane) results from reaction of a norbornadiene precursor with H_2_.^7^ The framework of [BAr^F^_4_]^−^ anions supports metal-centered reactivity,^7–9^ has CF_3_ groups that promote substrate diffusion^13,14^ and provides stability from noncovalent interactions.^9,10^ In this system, different anions, such as [Al­(OR^F^)_4_]^−^ ^7^ or [B­(3,5-Cl_2_–C_6_H_3_)_4_]^−^ ^15^ either do not support SC-SC reactivity or result in NBA displacement to ultimately form an arene-coordinated zwitterion.

The multistep synthesis^16^ of solvent-free precursor M­[BAr^F^_4_] (M = Li^+^, Na^+^, K^+^) creates a motivation to identify alternative anions that can facilitate SC-SC transformations. These anions should be cost-competitive and easily prepared on the gram scale as solvate-free salts of group 1 cations. The perfluoropinacol borate-based anion [B­(O_2_C_2_(CF_3_)_4_)_2_]^−^, **[FPB]** ^–^ (Chart 1), offers these advantages. While its potential for use in organometallic chemistry has been suggested,^17^ this has not been reported outside of a patent disclosing its use in olefin polymerization.^18^ The stability of its group 1 salts, however, is demonstrated in its use as battery electrolytes.^19–21^ We show here that **[FPB]** ^**–**^ can be used as a WCA for SMOM, by its use in the synthesis of a σ-alkane complex using SC-SC methods.

Solvate-free **Na­[FPB]** is synthesized from commercial perfluoropinacol and Na­[BH_4_] in THF solvent, using the reported method for group 1 cation salts, which have been structurally characterized as etherate solvates.^18–22^ Heating the resulting solid under dynamic vacuum (10^–2^ mbar) at 80 °C (18 h) forms free-flowing powdered **Na­[FPB]**.^22^ A single-crystal X-ray diffraction (SCXRD) study of **Na­[FPB]** (crystals isolated from hot 1,2-dichloroethane) revealed a 1D coordination polymer with B–O···Na linkages, Scheme 1A. Solution NMR data^22^ (THF-*d*_8_) showed no significant resonances associated with THF-*h*_8_ in the ^1^H NMR spectrum. The cost of preparing **Na­[FPB]** is competitive with Na­[BAr^F^_4_], ∼£10 versus £7/mmol (January 2025 online prices); while the Process Mass Intensity metric (PMI) favors **Na­[FPB]**, 22.6 versus 52.6.^23^ **[Caution!]** These advantages, however, should be balanced with the toxicity associated with the starting perfluoropinacol reagent;^24^ see the Supporting Information.

**Scheme 1:**
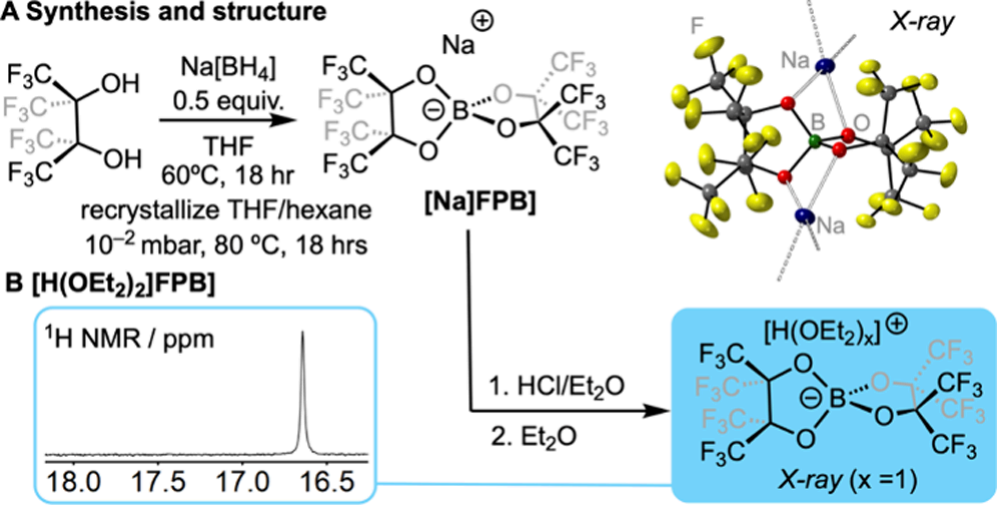
Synthesis of Na­[FPB] and [H­(OEt_2_)_2_]­[FPB]

The synthetically useful oxonium acid^6,25^ **[H­(OEt**_**2**_ **)**_**2**_ **]­[FPB]** can be prepared as a free-flowing white microcrystalline powder by addition of HCl to **Na­[FPB]** in Et_2_O solvent, following by filtration and removal of solvent [δ­(^1^H) = 16.6 *H*(OEt_2_)_2_, CD_2_Cl_2_], Scheme 1B. Addition of an excess of Proton Sponge (PS, 1,8-bis­(dimethylamino)­naphthalene) to **[H­(OEt**_**2**_ **)**_**2**_ **]­[FPB]** resulted in a mixture of unchanged PS and **[PS-H]­[FPB]**/Et_2_O, from which reliable integrals could be obtained in the ^1^H NMR spectrum. This allows for the determination of Et_2_O solvation of the oxonium acid in the bulk powder, i.e., [H­(OEt_2_)_2_]^+^. Analysis by SCXRD (crystals grown from CH_2_Cl_2_/Et_2_O) revealed two polymorphs, one of which gave a solvable structure (Supporting Information). This reveals this polymorph to have only one Et_2_O solvent molecule, which sandwiches the proton with the **[FPB]** ^**–**^ anion (see Figure S56).

The utility of **Na­[FPB]** as a supporting anion for SC-SC solid–gas reactivity is demonstrated by the synthesis of [Rh­(Cy_2_PCH_2_CH_2_PCy_2_)­(NBD)]­[FPB], **[1-NBD]­[FPB]** (NBD = norbornadiene), and its onward solid/gas reactivity with H_2_ to form the indefinitely stable σ-alkane complex [Rh­(Cy_2_PCH_2_CH_2_PCy_2_)­(*exo*-η^2^η^2^-NBA)]­[FPB], **[1-NBA]-[FPB]**, Figure 1A. Complex **[1-NBD]­[FPB]** is isolated as block-like red crystals by a straightforward route using **Na­[FPB]**, [Rh­(NBD)­Cl_2_]_2_ and Cy_2_PCH_2_CH_2_PCy_2_. The SCXRD structure shows an orthobifastigium arrangement of [FPB]^−^ anions surrounding two crystallographically equivalent [Rh­(Cy_2_PCH_2_CH_2_PCy_2_)­(NBD)]^+^ cations, in which the NBD ligands are directed toward each other (Figure S52). Solution and solid-state NMR (SSNMR) data support this formulation. Addition of H_2_ (1 bar) to crystals of **[1-NBD]­[FPB]** (50 mg scale) over 80 min results in the quantitative formation of **[1-NBA]­[FPB]** in a SC-SC transformation. The resulting ^31^P­{^1^H} SSNMR spectrum shows a characteristic^7^ downfield shift and increase in *J*(RhP) on formation of the σ-alkane complex (155 and 195 Hz), Figure 1B. In the ^13^C­{^1^H} SSNMR spectrum of **[1-NBA]­[FPB]** signals due to NBD are absent, with broad signals due to the anion observed at δ 121.8 (vbr), 86.4 (br).

**Figure 1:**
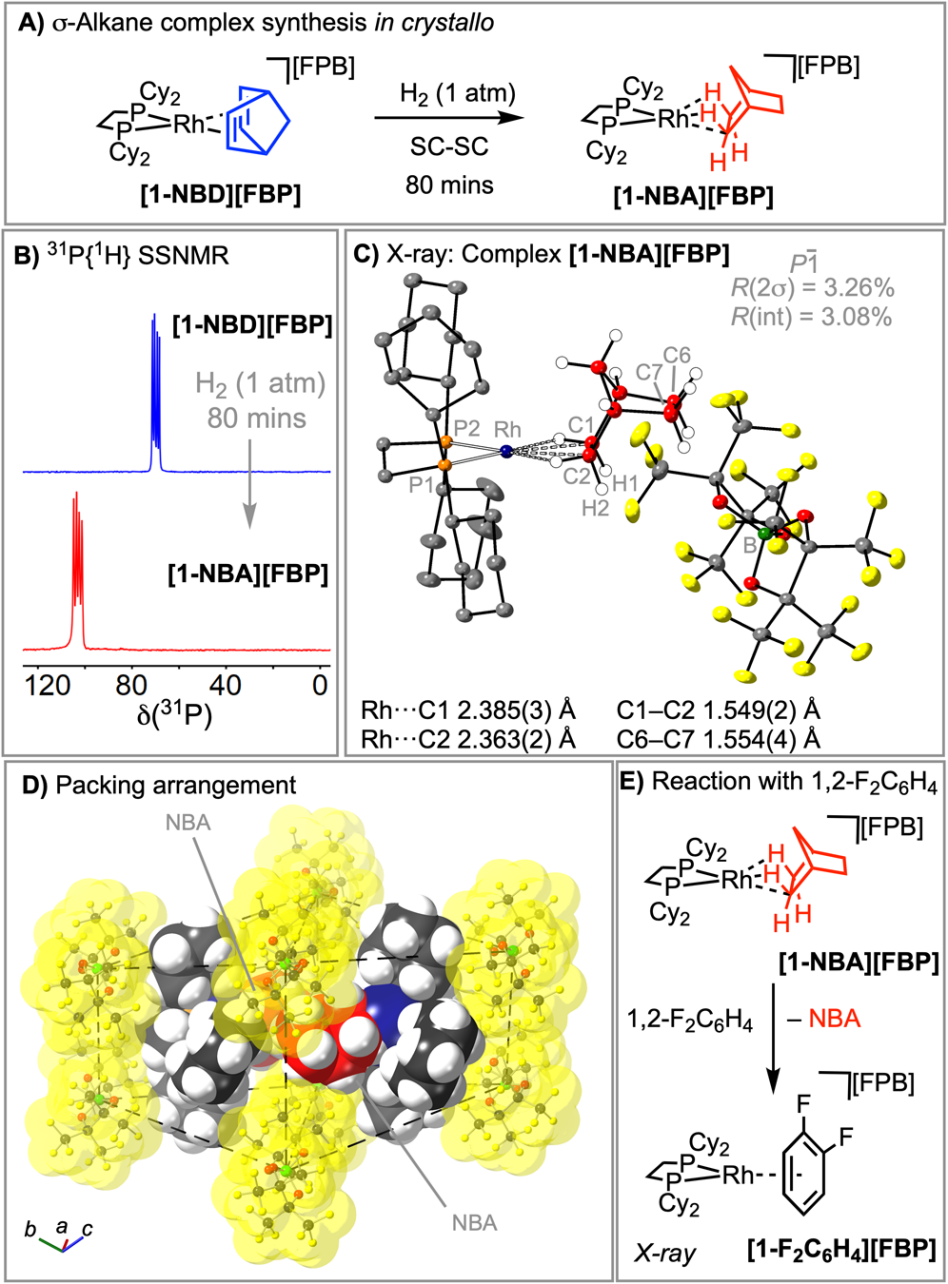
A) Synthesis of **[1-NBA]­[BAr** ^**F**^_**4**_ **]**. B) ^31^P­{^1^H} SSNMR of **[1-NBD]­[BAr** ^**F**^_**4**_ **]** and **[1-NBA]­[BAr** ^**F**^_**4**_ **]**. C) Molecular structure of the asymmetric unit in **[1-NBA]­[BAr** ^**F**^_**4**_ **]**. Displacement ellipsoids at the 40% level. D) Packing arrangement of anions, van der Waals surface. E) Reaction of **[1-NBA]­[BAr** ^**F**^_**4**_ **]** with 1,2-F_2_C_6_H_4_.

The SCXRD structure of **[1-NBA]­[FPB]** (Figure 1C) shows a NBA-alkane ligand binding through two 3c-2e Rh···H–C interactions [Rh···C 2.385(3) and 2.363(2) Å] to give a formally d_^8^_, 16-electron Rh­(I) center, similar to **[1-NBA]­[BAr** ^**F**^_**4**_ **]**.^7^ The H atoms associated with this interaction were located. However, in contrast with **[1-NBA]­[BAr** ^**F**^_**4**_ **]**, the chelating NBA ligand binds through two *exo*-C–H groups, the same as observed for the metastable **[1-NBA]­[B­(3,5-Cl**_**2**_ **-C**_**6**_ **H**_**3**_ **)**_**4**_ **]**.^15^ As for **[1-NBD]­[FPB]** the anions adopt an orthobifastigium arrangement (space group P-1, Figure 1D), and there is no significant change in the unit cell volume (3% difference). Uniquely for σ-alkane complexes synthesized using SMOM methods,^7–11^ this packing directs the alkane ligands in the crystallographically equivalent cations to face one another, and the metal centers to be relatively close (∼10 Å). As for **[1-NBA]­[BAr** ^**F**^_**4**_ **]**, complex **[1-NBA]­[FPB]** is stable at 298 K under an Ar atmosphere (1 month by ^31^P­{^1^H} SSNMR). It reacts rapidly with 1,2-F_2_C_6_H_4_ solvent to displace the alkane to form the arene-adduct, **[1-F**_**2**_ **C**_**6**_ **H**_**4**_ **]­[FPB]**,^11^ as characterized by SCXRD (Figure 1E, Figure S54).

**[1-NBA]­[FPB]** was characterized computationally using periodic-DFT calculations. Geometry optimization relaxing the H atom positions shows elongation of the C1–H1A and C2–H2B bonds to 1.17 Å, and NBO calculations indicate dominant C–H → Rh σ-donation (Figure 2A). These, and other computed metrics (Figures S33–S36), all signal a σ-alkane complex. The η^2^_C–H_ binding mode is also reflected in the computed Rh···H–C angles (102°) and the Independent Gradient Model plot based on Hirshfeld partitioning (IGMH, Figure 2B).

**Figure 2:**
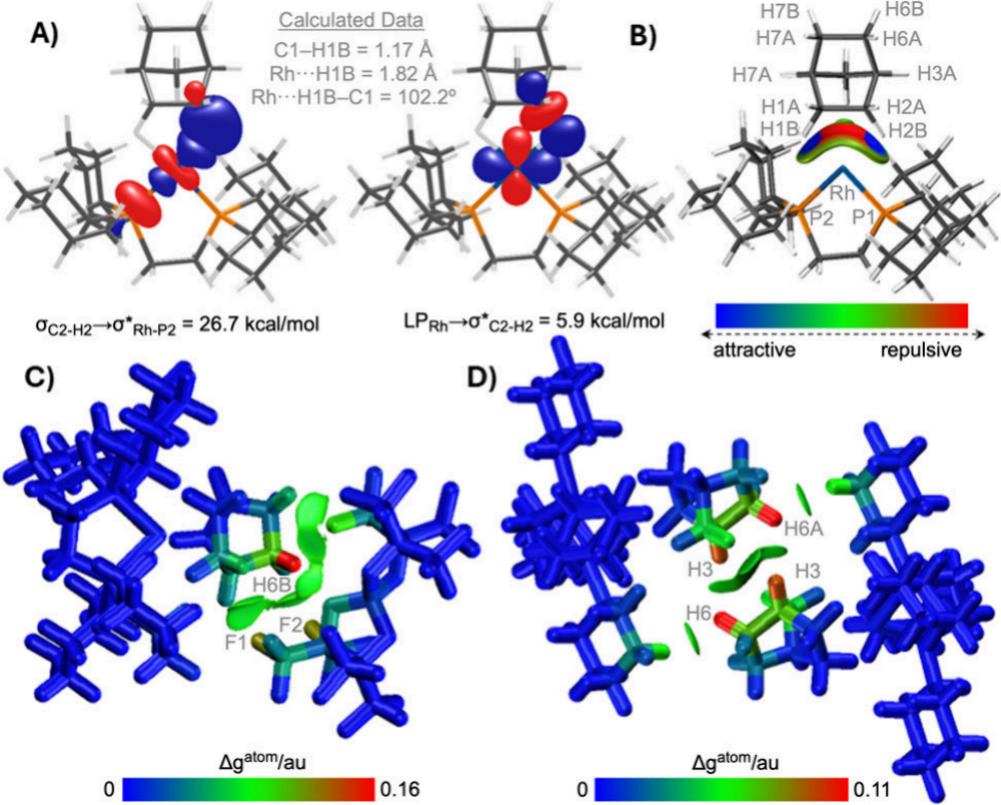
A) Key donor–acceptor interactions for the Rh-NBA interaction in the cation of **[1-NBA]­[FPB]** as quantified via second-order perturbation NBO analyses. B) IGMH plot for the cation highlighting the Rh-NBA interaction (sign­(λ_2_)­ρ-colored isosurfaces with δG_inter_ = 0.003 au) C) IGMH plot of the cation–anion pair and D) IGMH plot of cation–cation pair (atoms colored by %δG^atom^). See Supporting Information for IGMH plots of other nearest-neighbor ion pairs.

The NBA solid-state binding energy in **[1-NBA]­[FPB]** (i.e., the energy required to remove one NBA ligand from the unit cell) is computed to be 49.5 kcal/mol and compares with a molecular binding energy (for the isolated cation) of 36.5 kcal/mol. This gives a solid-state stabilization energy (SSSE) of 12.5 kcal/mol, similar to that computed for the *endo*-bound NBA in **[1-NBA]­[BAr** ^**F**^_**4**_ **]** (14.0 kcal/mol).^9^ For **[1-NBA]­[BAr** ^**Cl**^_**4**_ **]** (which also exhibits *exo*-NBA binding), the NBA solid-state binding energy is 50.7 kcal/mol, and the SSSE is 14.9 kcal/mol. Thus, the [BAr^F^_4_]^−^ and [FPB]^−^ lattices in **[1-NBA]­[BAr** ^**F**^_**4**_ **]** and **[1-NBA]­[FPB]** have similar influence on alkane binding across different topologies and NBA binding modes. In **[1-NBA]­[BAr** ^**Cl**^_**4**_ **]** the computed *exo* to *endo* rearrangement of one NBA ligand within the unit cell resulted in a destabilization of +2.3 kcal/mol. In **[1-NBA]­[FPB]** this is computed to be +8.4 kcal/mol, while in the absence of any solid-state environment this difference collapses to 0.3 kcal/mol. This emphasizes the role of the solid-state 2° microenvironment in controlling structure and stability.^26,27^

The role of the 2° microenvironment was further probed via IGMH plots of nearest neighbor ion pairs, in particular those highlighting interactions between the NBA ligand and the adjacent **[FPB]** ^–^ anion (Figure 2C) and between NBA ligands arising from the unusual back-to-back cation packing motif (Figure 2D). In both cases, green isosurfaces indicate regions of stabilizing dispersion interactions, while the atom color coding highlights the largest % atomic contributions in red. H6B···F1/F2 contacts contribute most to NBA···**[FPB]** ^**–**^ dispersion while the H3···H6A contacts are most prominent in the cation–cation pair. These interactions reflect correspondingly short computed nonbonded distances (H6A···F1 = 2.39 Å; H6A···F2 = 2.61 Å; H3···H7B = 2.49 Å). In total, forty-two short H···F contacts (i.e., <Σ_vdW radii_ ^28^ + 10%) are present around the cation in **[1-NBA]­[FPB]**. A further estimate of interion dispersion comes from the computed cation–anion interaction energies: with PBE+D3 these range from 32 to 68 kcal/mol and these drop by 5–15% when recomputed without the D3 correction (Figures S38–S45).

In conclusion, we show that the **[FPB]** ^**–**^ anion supports SC-SC solid/gas reactivity at reactive cationic metal centers. The ease of synthesis of a variety of synthetically useful salts, robustness, and low cost of **[FPB]** ^–^ mean that it perhaps should be considered more widely in the toolbox of organometallic and main-group chemistry more generally, when suitable precautions are taken for the safe handling of perfluoropinacol and its derivatives.

## Supplementary Material





## Notes

Full experimental details, characterization, NMR spectra, cost analysis, computational details, electronic structure analyses, and X-ray crystallographic figures (PDF)
Computed geometries (XYZ)
