# Supplementary material for: [B(O2C2(CF3)4)2]− ([FPB]−): Repurposing This Weakly Coordinating Anion for Solid-State Molecular Organometallic (SMOM) Chemistry
Source: Organometallics. 2025 May 4;44(10):1018–21. doi: 10.1021/acs.organomet.5c00112 (PMC12117562; doi:10.1021/acs.organomet.5c00112)
Supplement: Supplementary file 1 [file om5c00112_si_001.pdf]

Supporting Information

$[B(O_2C_2(CF_3)_4)_2]^-$ ($[FPB]^-$): Repurposing this Weakly Coordinating Anion for Solid-State Molecular OrganoMetallic (SMOM) Chemistry

Kristof M. Altus,[a] M. Arif Sajjad,[b] Stuart A. Macgregor[b]* and Andrew S. Weller[a]*

[a]Department of Chemistry, University of York, York, YO10 5DD, UK

[b]EaSTCHEM School of Chemistry, North Haugh, University of St. Andrews, St Andrews KY16 9ST, UK.

Email: andrew.weller@york.ac.uk

Email: sam38@st-andrews.ac.uk

Contents

S1.0 Experimental 3
S1.1 General considerations 3
S1.2 Synthesis of compounds 4
S1.3 NMR spectra 9
Na[FPB] 9
[H(OEt$_2$)$_2$][FPB] 11
[1-NBD][FPB] 13
[1-NBA][FPB] 18
[1-F$_2$C$_6$H$_4$][FPB] 19
S2.0 Additional NMR data 23
S2.1 Solid-state stability of complex **[1-NBA][FPB]** 23
S2.2 Solution-state NMR of [Na(THF)$_n$][FPB] after recrystallisation and post drying at 80 °C. 24
S2.3 Low temperature solution NMR analysis of the product of dissolving complex 3 in dichloromethane-d^2 followed by addition of 1,2-difluorobenzene. 25
S2.4 Reaction of [H(OEt$_2$)$_x$[FPB] with proton sponge (PS). 27
S3.0 Cost analysis of in-house synthesised Na[BAr$^F{}_4$] vs Na[FPB] 28
S4.0 Computational Details 30
S4.1 Electronic Structure Analyses. 31
S4.2 Cation-Anion Ion-pair Interactions 37
S4.3 Cation-Cation Ion-pair Interactions 45
S5.0 X-Ray Crystallography 50
S5.1 Crystallographic Table of Data 51
S5.2 X-Ray crystallographic Figures 52
S6.0 References 58

S1.0 Experimental

S1.1 General considerations

All manipulations, unless otherwise stated, were performed under an inert (argon or nitrogen, BOC, N4.8 purity) atmosphere using standard Schlenk line and glovebox (<0.1 ppm H_2O/O_2) techniques. Glassware was oven-dried at 140 °C overnight. All solvents were degassed by three successive freeze-pump-thaw cycles and stored over activated 3 Å molecular sieves under inert gas in resealable glass ampoules fitted with PTFE high vacuum stopcocks (J. Youngs or Rotaflo HP). CH_2Cl_2 and hexane were dried using a commercially available solvent system (MBraun) by passage through stainless steel columns containing activated alumina. CD_2Cl_2 was dried over CaH_2, before vacuum transfer and storage as above. THF was dried over sodium fluorenone, distilled and stored over molecular sieves prior to use. Hexafluoro-2,3-bis(trifluoromethyl)-2,3-butanediol (perfluoropinacol) was purchased from Fluorochem and degassed prior to use.

Solution state NMR data were collected on a Bruker AVIIIHD 500 MHz or AVIIIHD 600 MHz Widebore spectrometer at the temperatures specified. Multiplicity abbreviations: singlet (s), doublet (d), triplet (t), multiplet (m). Coupling constants are designated as $^nJ_{X-Y}$ for each respective nucleus. Solution ^1H and $^{13}C\{^1H\}$ NMR spectra were referenced to the residual solvent peaks. All NMR assignments for complexes were made based on 1D NMR data as well as crystallographic analysis where relevant. $^{31}P\{^1H\}$ solution spectra were referenced externally to 85% H_3PO_4 in D_2O. All dichloromethane-d^2 solution phase NMR were prepared on a greaseless high vacuum line (<5 x 10^{-2} mbar) by condensation of the solvent under static vacuum onto solid samples in 5 mm thin wall NMR tubes fitted with high vacuum PTFE (J. Youngs) valves.

Solid-state NMR samples were prepared in an argon or nitrogen-filled glovebox by pre-loading 10-20 mg (2.0 mm) of crushed material into zirconia solid-state NMR rotors and sealed with Kel-F, vespel or zirconia caps. Solid-state NMR data were obtained on Bruker Avance III HD spectrometers, operating at 100.63 MHz ($^{13}C\{^1H\}$), 100.56 MHz ($^{13}C\{^1H\}$), 162.04 MHz ($^{31}P\{^1H\}$), 161.99 MHz ($^{31}P\{^1H\}$) at the MAS rates and temperatures specified. All $^{13}C\{^1H\}$ CP MAS spectra were referenced to adamantane where the upfield methine resonance was taken to be δ_C = 29.5 ppm, secondarily referenced to $\delta_C SiMe_4$) = 0.0 ppm. $^{31}P\{^1H\}$ CP MAS spectra were referenced to triphenylphosphine (δ_P = -9.3 ppm relative to H_3PO_4) or calcium hydrogen phosphate (δ_P = 1.4 ppm relative to H_3PO_4). Elemental analysis was carried out by Ms Orla McCullough at London Metropolitan University.

S1.2 Synthesis of compounds

SAFETY NOTICE ON HANDLING PERFLUOROPINACOL.

Hexafluoro-2,3-bis(trifluoromethyl)-2,3-butanediol (perfluoropinacol) is extremely toxic and should be handled with extreme care. The LD_{50} Oral (mouse) is 600 mg/kg, the LD_{50} Dermal is 5 mg/kg (SDS Merck). Two pairs of nitriles gloves were worn when handling the liquid. All manipulations were carried out in a fume hood with the sash as low as possible. Perfluoropinacol was decanted from its commercial bottle into a pre-dried J-young's ampoule. The liquid is degassed via three sequential freeze pump thaw cycles and used in its entirety in the synthesis below, minimising the number of times the liquid is handled. We suggest that all contaminated glassware and syringes/cannulas that are used are washed with a KOH/isopropanol solution in a fume hood, subsequent waste should be disposed of as halogenated wate.

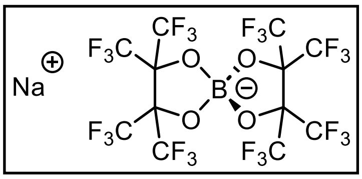

$Na[B(O_2C_2(CF_3)_4)_2]$, Na[FPB]: Synthesis was adapted from the literature for the synthesis of the Lithium derivative.[1] Under an atmosphere of argon, $Na[BH_4]$ (277 mg, 7.48 mmol) was added to a large round bottom flask fitted with a PTFE stopcock. Degassed Hexafluoro-2,3-bis(trifluoromethyl)-2,3-butanediol (5 g, 14.9 mmol) was then added as a solution in THF (15 mL) (Caution! hydrogen was released immediately), the reaction was stirred for one hour at room temperature prior to heating to reflux overnight (~18 hours) under a flow of N_2. The volatiles were removed under reduced pressure until a white solid was left. The solid was redissolved in THF (10 mL) and layered with hexane (50 mL) at room temperature. After several days large colourless poorly diffracting crystals formed which are assigned as $[Na(THF)_n][FPB]$. The crystals were decanted and dried overnight under reduced pressure (2 x 10^{-2} mbar) at 80 °C giving 3.06 g of the solvate free salt in 58% yield as a white crystalline solid.

Crystals of the solvate free salt suitable for X-ray diffraction were grown by suspending 1-2 mg of **Na[FPB]** in dry and degassed dichloroethane under argon, followed by heating at 80 °C for three days until small needles were deposited.

$^{13}C\{^1H\}$ NMR (THF-d8, MHz, 298 K): δ 123.19 (q, J_{C-F} = 294 Hz, C(**CF_3**)$_2$), 86.91 (br m, **C**$(CF_3)_2$).

^{11}B NMR (THF-d8, MHz, 298 K): δ 11.56 (s)

^{19}F NMR (THF-d8, MHz, 298K): δ –70.33 (s)

Elemental Analysis: Calculated for $C_{12}BF_{24}NaO_4$: C 20.65%; H 0%; Found: C 20.58%; H 0%.

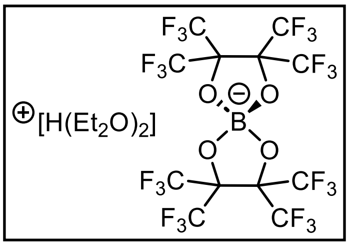

[H(Et_2O)$_2$][FPB]: To an ampoule fitted with a PTFE stopcock under an argon atmosphere was added Na[FPB] (500 mg, 0.71 mmol) and Diethyl ether (5 mL). To this solution was added HCl dropwise as a solution in diethyl ether (0.75 mL, 1.5 mmol, 2 M) at 0 °C. A white precipitate was immediately observed, the reaction was allowed to stir for 10 mins whilst warming to room temperature. The suspension was then filtered into a fresh flask washing the white solid with more diethyl ether (4 x 5 mL). The solvent was then removed under reduced pressure giving a white solid. The solid was dissolved in equal volumes of diethyl ether and dichloromethane and layered with 20 volumes of hexane. After several days large colourless crystals grew, the solvent was decanted and the crystals dried overnight under dynamic vacuum (2 x 10^{-2} mbar) giving 450 mg of a white solid in 85 % yield.

The acid is stable in dichloromethane and diethyl ether for at least three days. At temperatures below 0 °C the compound starts to precipitate in both dichloromethane and diethyl ether.

^{1}H NMR (CD_2Cl_2, 500 MHz, 243 K): δ 16.60 (broad s, **H**(OEt_2), 1H), 4.06 (br m, O(C**H**$_2$$CH_3$)$_2$), 1.41 (br m, O($CH_2$C**H**$_3$)$_2$).

^{19}F NMR (CD_2Cl_2, 476 MHz, 243 K): δ –70.15 (s)

^{11}B NMR (CD_2Cl_2, 161 MHz, 243 K): δ 11.25 (s)

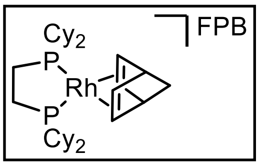

[($(Cy_2PCH_2CH_2PCy_2)Rh(NBD)$)][FPB], [1-NBD][FPB]: To a J-youngs ampoule under argon was added $[Rh(NBD)Cl]_2$ (37.5 mg, 0.081 mmol), which was subsequently dissolved in dichloromethane (5 mL). Norbornadiene (50 µL, 0.6 mmol) was added via syringe and the reaction stirred for 30 minutes at room temperature before addition of dicyclohexylphosphino ethane (63.0 mg, 0.15 mmol) as a solution in dichloromethane (1 mL). The reaction was stirred for another 30 minutes before addition of Na[FPB] (103 mg, 0.15 mmol) as a solution in THF (1 mL). The reaction was stirred overnight at room temperature (~18 hours). Removal of all volatiles under reduced pressure gave an orange powder which was washed with hexane (3 x 5 mL), dissolved in dichloromethane (1.5 mL) and cannula filtered into a recrystallisation ampoule and layered with hexane. After a week, red block crystals had formed. Decanting the solution and drying under vacuum gave 165 mg of red coloured crystals in 84 % yield.

^{1}H NMR (CD_2Cl_2, 500 MHz, 298 K): δ 5.55 (m, NBD alkene, 4H), 4.21 (m, NBD bridgehead, 2H), 2.07 – 1.96 (overlapping aliphatic resonances), 1.94 – 1.81 (overlapping aliphatic resonances), 1.82–1.00 (overlapping aliphatic resonances), 1.38 – 1.18 (overlapping aliphatic resonances), 1.16 – 1.03 (overlapping aliphatic resonances).

^{13}C{^{1}H} NMR (CD_2Cl_2, 125 MHz, 298 K): δ 122.25 (q, J_{F-C} = 294 Hz, C(***C***$F_3)_2$, 86.39 (ddd, *J* = 6.5, 4.9, 3.8 Hz, NBD alkene), 85.93 (br, **C**$(CF_3)_2$) 72.21 (m, NBD **C**H_2 bridge), 56.26 (m, **C**H NBD bridgehead), 36.01 (m, P-**C**H), 30.01 (s, cyclohexyl **C**H_2), 29.38 (s, cyclohexyl **C**H_2), 27.47, 27.10 (complex multiplets, cyclohexyl **C**H_2's), 26.30 (s, cyclohexyl **C**H_2, 21.54 (ddd, J = 21.1, 18.0, 3.4 Hz, P-**C**H_2).

^{31}P{^{1}H} NMR (CD_2Cl_2, 202MHz, 298 K): δ 69.79 (d, J_{Rh-P} = 154 Hz)

^{11}B NMR (CD_2Cl_2, 160 MHz, 298 K): δ 11.40 (s)

^{19}F NMR (CD_2Cl_2, 470 MHz, 298 K): δ –70.13 (s)

^{13}C{^{1}H} CPMAS SSNMR (100 MHz, 20 KHz, 298 K): δ 123.70 (very broad, FWH: ~820 Hz, C(**C**$F_3)_2$), 88.11 (NBD alkene), 87.80 (NBD alkene), 88.60 (br overlapping, **C**$(CF_3)_2$), 85.62 (NBD alkene), 84.06 (NBD alkene), 70.81 (NBD bridge), 56.29 (NBD bridgehead), 39.74, 37.18, 36.54, 33.23, 32.23, 31.38, 29.21, 28.28, 27.85, 26.95, 26.30, 25.85, 23.68, 21.43 (multiple aliphatic resonances).

^{31}P{^{1}H} CPMAS SSNMR (162 MHz, 20 KHz, 298 K): δ 70.85 (d, J_{Rh-P} = 155 Hz), 68.85 (d, J_{Rh-P} = 155 Hz)

Elemental Analysis: Calculated for $C_{45}H_{56}BF_{24}O_4P_2Rh$: C 41.82 %; H 4.37 %; Found: C 41.85 %; H 4.48 %.

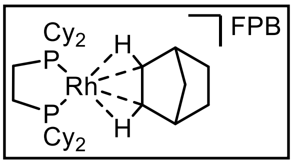

[(Cy$_2$PCH$_2$CH$_2$PCy$_2$)Rh(norbornane)][FPB], [1-NBA][FPB]: To a J-Youngs ampoule was added **[1-NBD][FPB]** (50 mg, 0.038 mmol). The ampoule was evacuated down to 2 x 10^{-2} mbar before being backfilled with hydrogen (2 bar). The reaction was left for 80 minutes before removing the hydrogen atmosphere and replacing with argon giving orange-red crystals. Dissolving some of the crystals in 1,2-difluorobenzene and recording the $^{31}P\{^1H\}$ NMR spectrum showed only the presence of complex **[1-F$_2$C$_6$H$_4$][FPB]** with no traces of complex **[1-NBD][FPB]** consistent with quantitative formation of complex **[1-NBA][FPB]**.

$^{13}C\{^1H\}$ CPMAS SSNMR (100 MHz, 20 KHz, 298 K): δ 121.77 (Br, C**C**F$_3$), 86.38 (**C**CF$_3$), 40.40, 38.96, 37.75, 32.34, 31.90, 30.58, 29.59, 28.60, 27.54, 26.45, 25.53, 23.00, 21.54 (Multiple aliphatic resonances.

$^{31}P\{^1H\}$ CPMAS SSNMR (162 MHz, 20 KHz, 298 K): δ 104.19 (d, $^1J_{Rh\text{-}P}$ = 195 Hz), 101.89 (d, $^1J_{Rh\text{-}P}$ = 195 Hz)

Elemental Analysis: Calculated for $C_{45}H_{60}BF_{24}O_4P_2Rh$: C 41.69 %; H 4.66 %; Found; C 40.30 %; H 4.70 %.

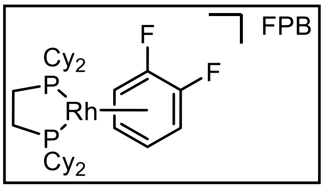

[($Cy_2PCH_2CH_2PCy_2$)Rh(η^6-1,2-$F_2C_6H_4$)].(1,2-$F_2C_6H_4$)[FPB], [1-$F_2C_6H_4$][FPB]: To a J-Young's ampoule was added complex **[1-NBA][FPB]** (34 mg, 0.026 mmol). The solid was dissolved in 1,2-$F_2C_6H_4$ (1 mL), which liberates norbornane instantaneously with the concomitant formation of complex **[1-$F_2C_6H_4$][FPB]** as measured by $^{31}P\{^1H\}$ NMR spectroscopy. The resulting solution was layered with hexane (10-15 mL) at room temperature. After several days yellow-orange crystals grew, the solvent was decanted, and the crystals dried under reduced pressure overnight at 2 x 10^{-2} mbar giving 31 mg of yellow-orange crystals in 83 % yield.

^{1}H NMR (CD_2Cl_2, 500 MHz, 298 K): δ 7.24 – 7.14 (m, 1,2-$F_2C_6H_4$ of recrystallisation, 2H), 7.14 – 7.09 (m, 1,2-$F_2C_6H_4$ of recrystallisation, 2H), 6.91 – 6.78 (m, 1 Rh bound 1,2-$F_2C_6H_4$, 2H), 6.31 – 6.20 (m, Rh bound 1,2-$F_2C_6H_4$, 2H), 2.00 – 1.81 (m, aliphatic resonances), 1.81 – 1.59 (m, aliphatic resonances), 1.43 – 1.11 (m, aliphatic resonances), 1.10 – 0.95 (m, aliphatic resonances).

$^{13}C\{^1H\}$ NMR (CD_2Cl_2, 125 MHz, 298 K): δ 151.93-149.85 (dd, $J_{C\text{-}F}$ = 248, 14 Hz, 2-difluorobenzene of recrystallisation, **C**-F), 136.55-134.33 (dd, Rh bound 1,2-$F_2C_6H_4$, $J_{C\text{-}F}$ = 278, 16Hz, **C**-F), 122.06 (q, $J_{F\text{-}C}$ = 294 Hz, C(**C**F_3)$_2$), 125.13 (apparent triplet, J = 5.2 Hz, 1,2-$F_2C_6H_4$ of recrystallisation, ***m*-1,2-$F_2C_6H_4$**), 117.70 (dd, $J_{C\text{-}F}$ = 11.9, 5.5 Hz, ***o*-1,2-$F_2C_6H_4$**), 94.00 (m, Rh bound **1,2-$F_2C_6H_4$**), 90.99 (m, Rh bound **1,2-$F_2C_6H_4$**), 38.29 (m, P-**C**H), 29.36 (s, cyclohexyl **C**H_2 resonance), 29.26 (s, cyclohexyl **C**H_2 resonance), 27.19, 26.98 (complex multiplet, cyclohexyl **C**H_2's), 26.34 (s, cyclohexyl **C**H_2 resonance), 23.01 (ddd, $J_{P\text{-}C}$ = 24.7, 18.6, 5.4 Hz, P-**C**H_2).

$^{31}P\{^1H\}$ NMR (CD_2Cl_2, 202MHz, 298 K): δ 98.11 (d, $^1J_{Rh\text{-}P}$ = 199 Hz)

^{11}B NMR (CD_2Cl_2, 160 MHz, 298 K): δ 11.37 (s)

^{19}F NMR (CD_2Cl_2, 470 MHz, 298 K): δ –70.11 (s), -139.4 (1,2-$F_2C_6H_4$ of recrystallisation), -145.7 ($J_{Rh\text{-}F}$ = 4 Hz, Rh bound 1,2-$F_2C_6H_4$)

Elemental Analysis: Calculated for $C_{50}H_{56}BF_{28}O_4P_2Rh$: C, 42.04 %; H 3.95 %; Found: C 41.55 %; H 4.00 %.

S1.3 NMR spectra

Na[FPB]

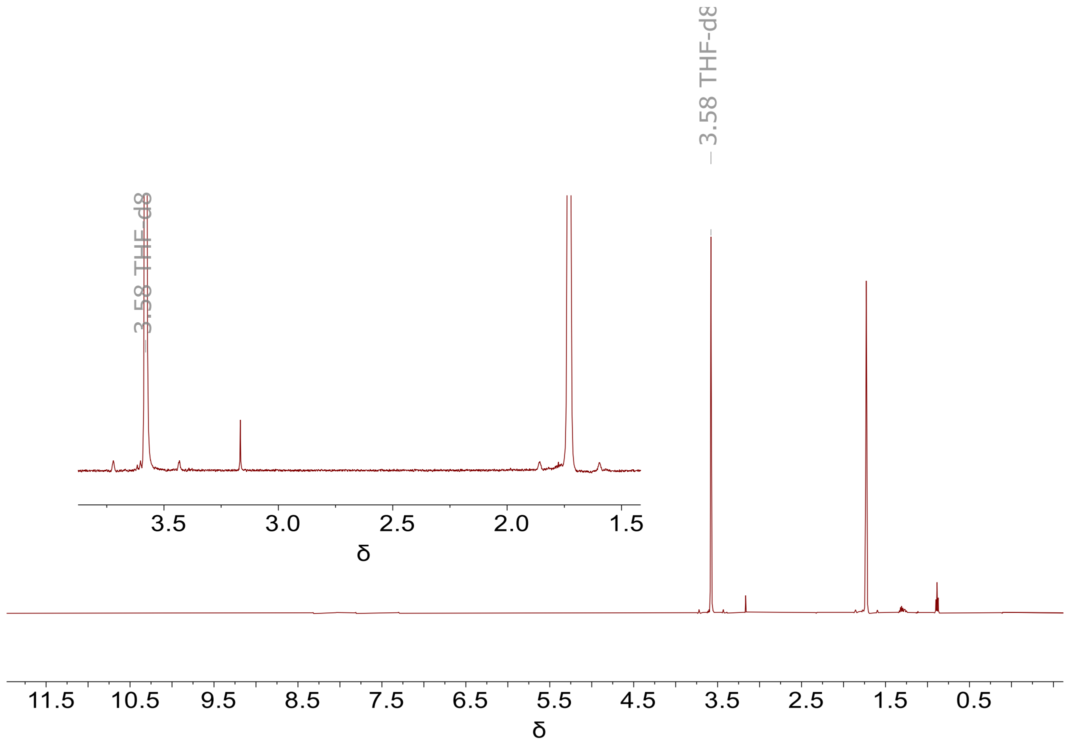

Figure S1. ^{1}H NMR spectrum (THF-d^{8}, 500 MHz, 298 K) of **Na[FPB]**.

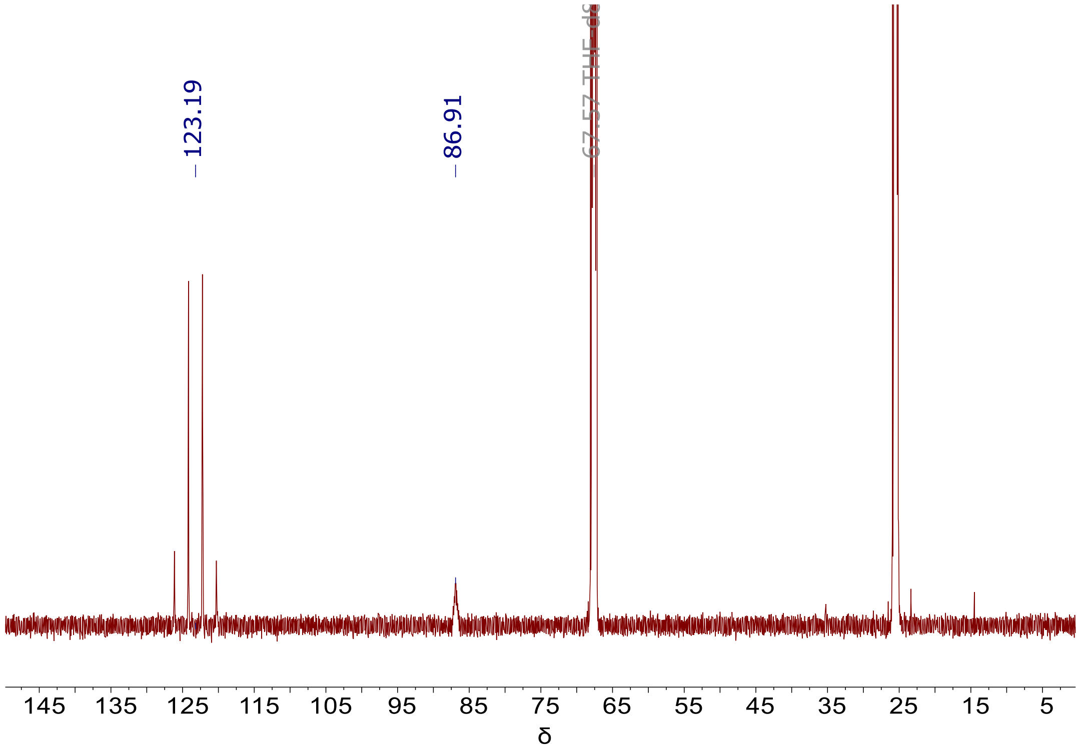

Figure S2. $^{13}C\{^{1}H\}$ NMR spectrum (THF-d^{8}, 150 MHz, 298 K) of **Na[FPB]**.

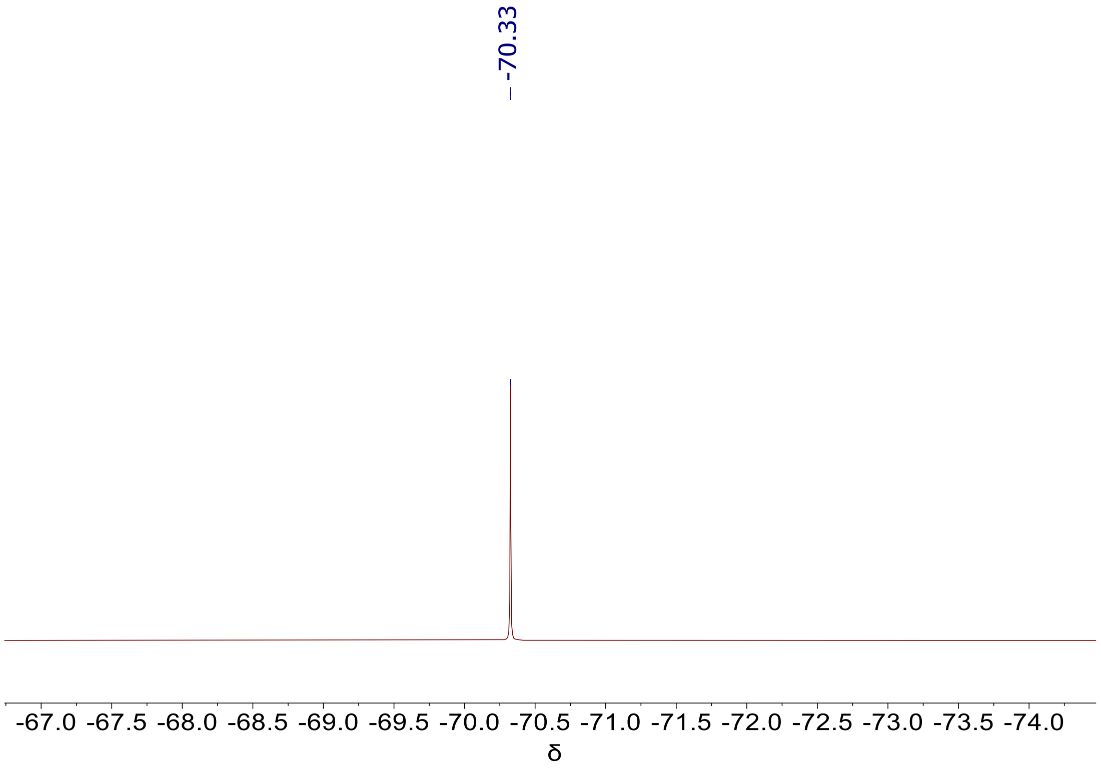

Figure S3. ^{19}F NMR spectrum (THF-d[8], 470 MHz, 298 K) of **Na[FPB]**

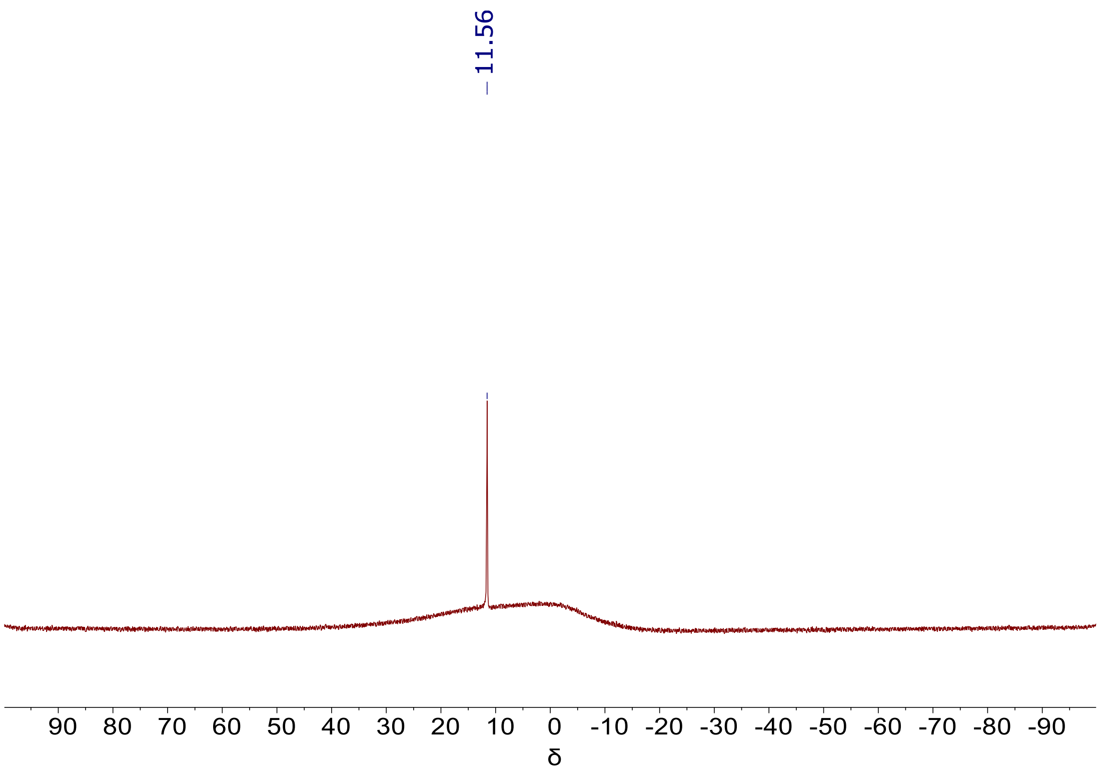

Figure S4. ^{11}B NMR spectrum (THF-d[8], 192 MHz, 298 K) of **Na[FPB]**

[H(OEt$_2$)$_2$][FPB]

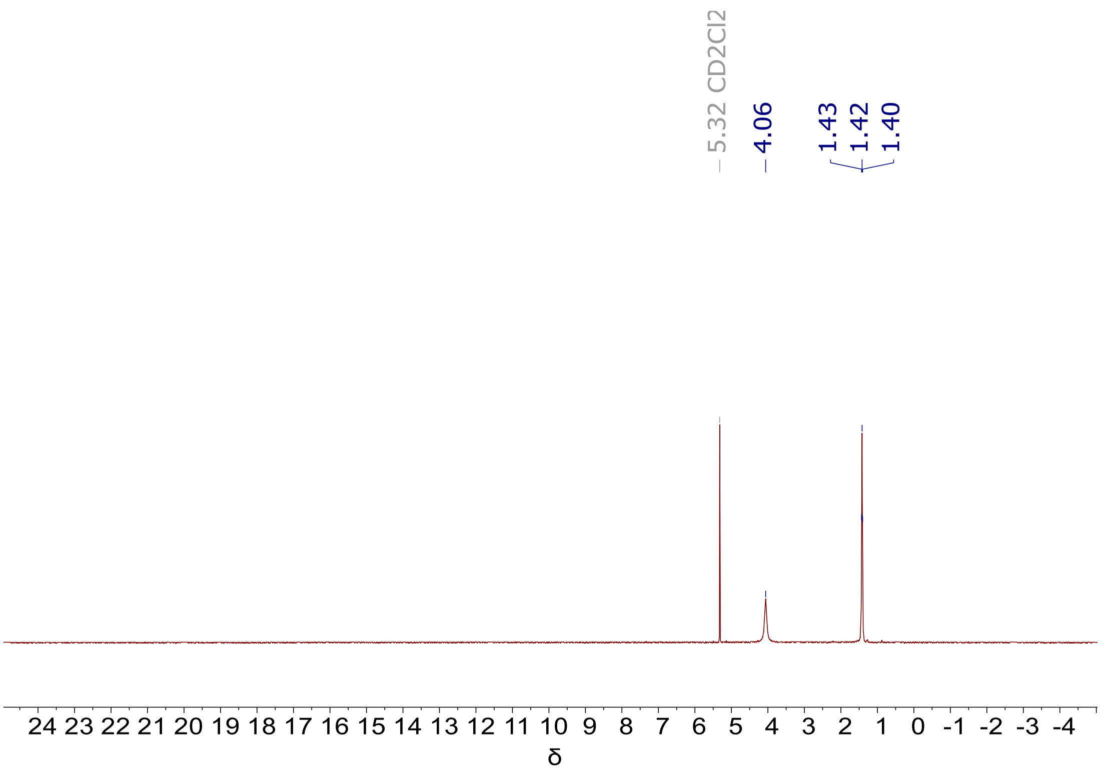

Figure S5. ^{1}H NMR spectrum (CD_2Cl_2, 500 MHz, 298 K) of **[H(OEt$_2$)$_2$][FPB]**.

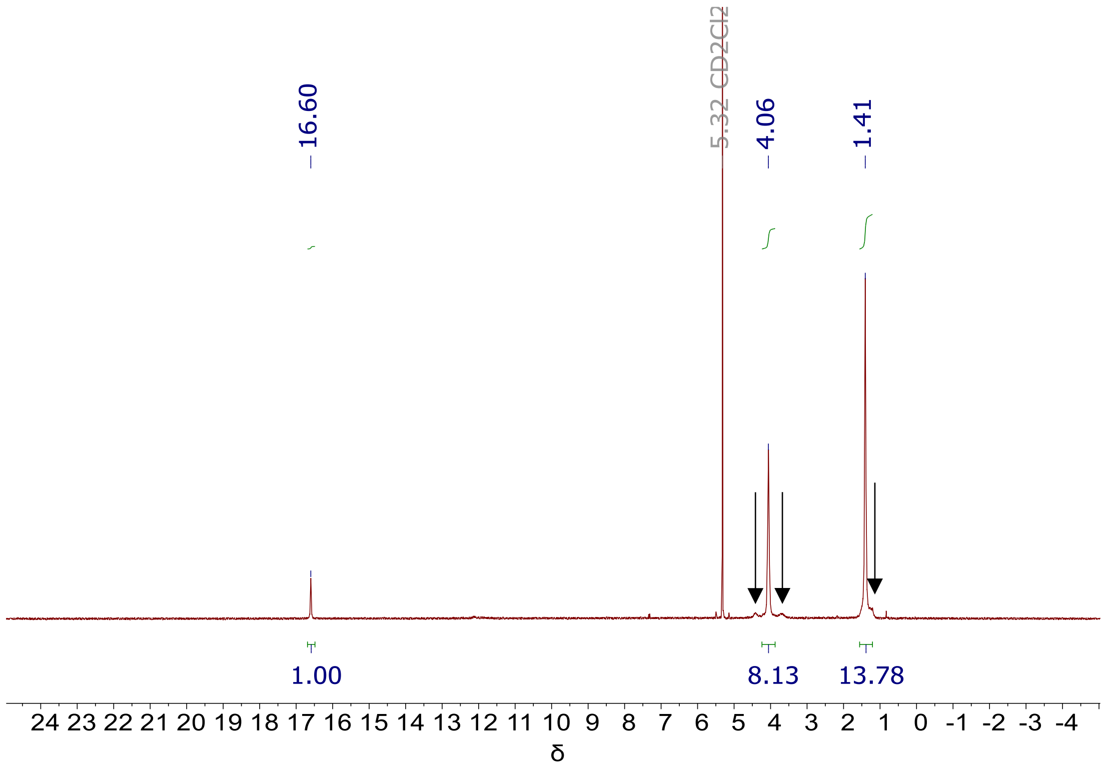

Figure S6. ^{1}H NMR spectrum (CD_2Cl_2, 500 MHz, 243 K) of **[H(OEt$_2$)$_2$][FPB].** Arrows denote signals that appear at low temperature, which we assign due to precipitation of complex (precipitate observed in the NMR tube). Integrals approximate to two diethyl ether molecules per proton. The disappearance of the proton signal at room temperature is consistent with that also observed for Brookhart's acid and suggests that rapid exchange is occurring. [2]

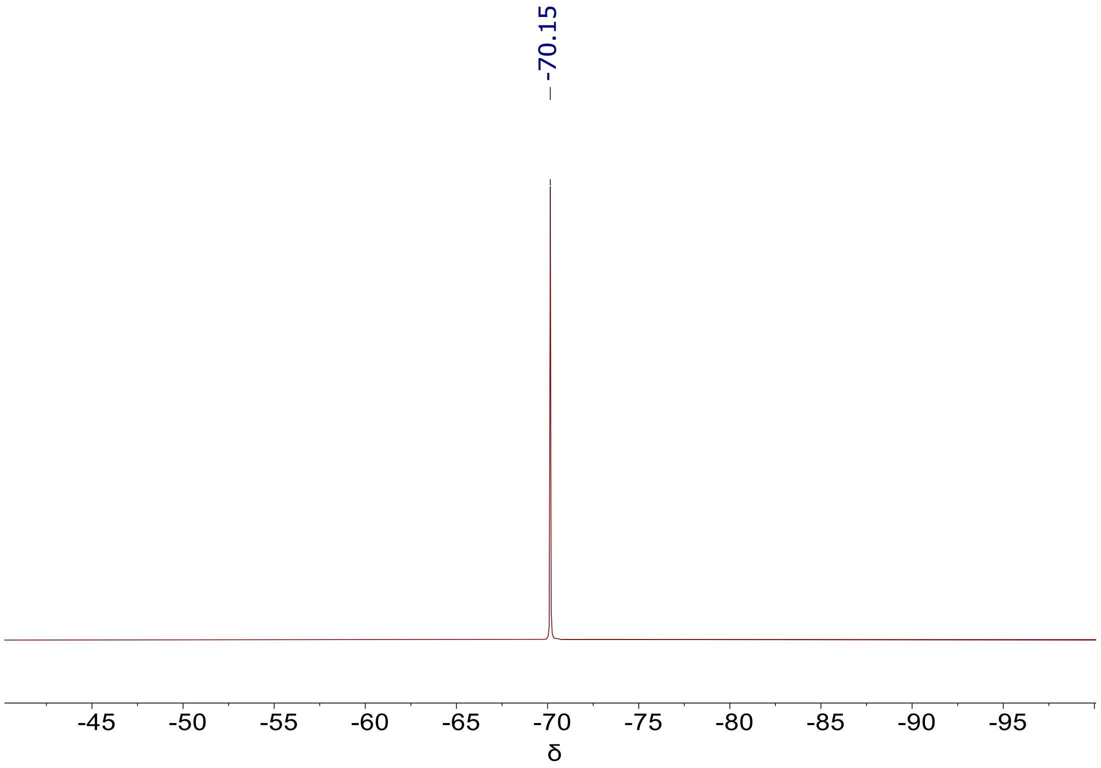

Figure S7. ^{19}F NMR spectrum (CD_2Cl_2, 470 MHz, 243 K) of **[H(OEt$_2$)$_2$][FPB]**.

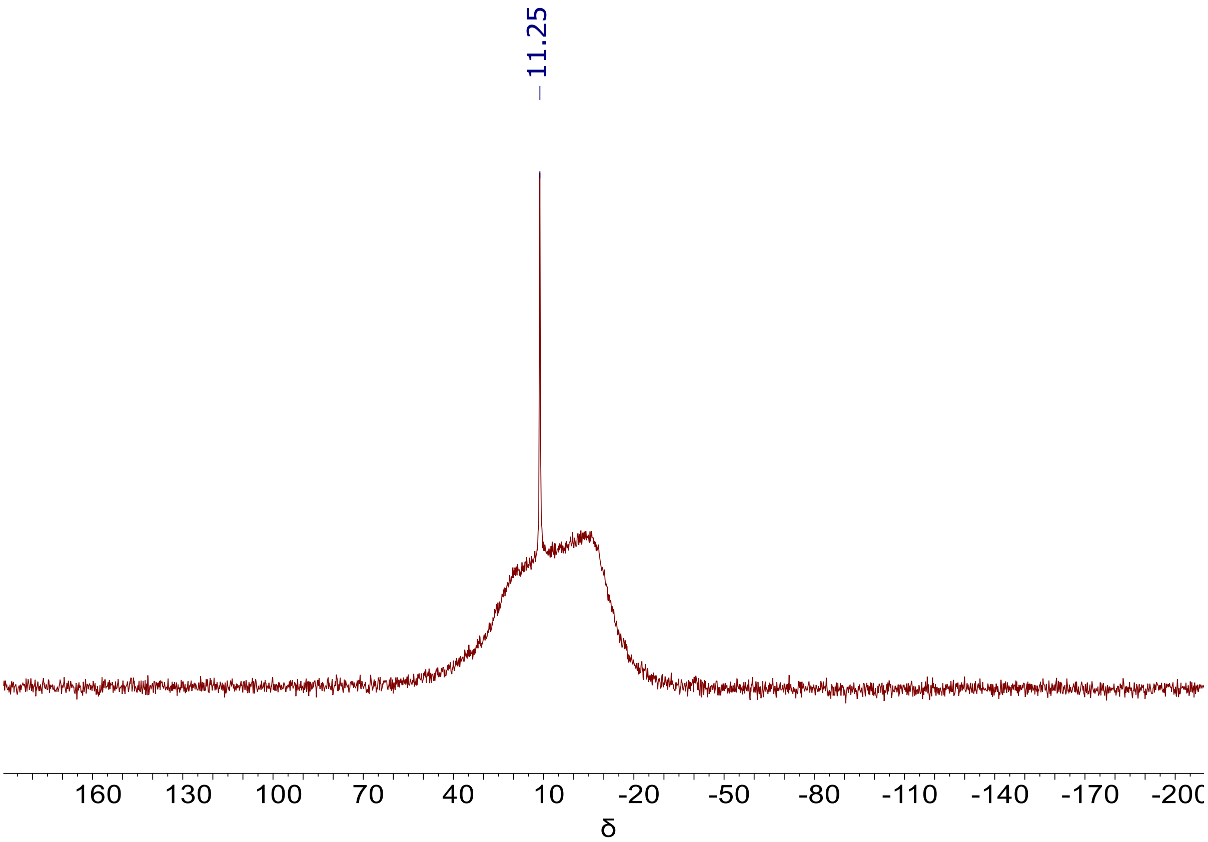

Figure S8. ^{11}B NMR spectrum (CD_2Cl_2, 160 MHz, 243 K) of **[H(OEt$_2$)$_2$][FPB]**.

[1-NBD][FPB]

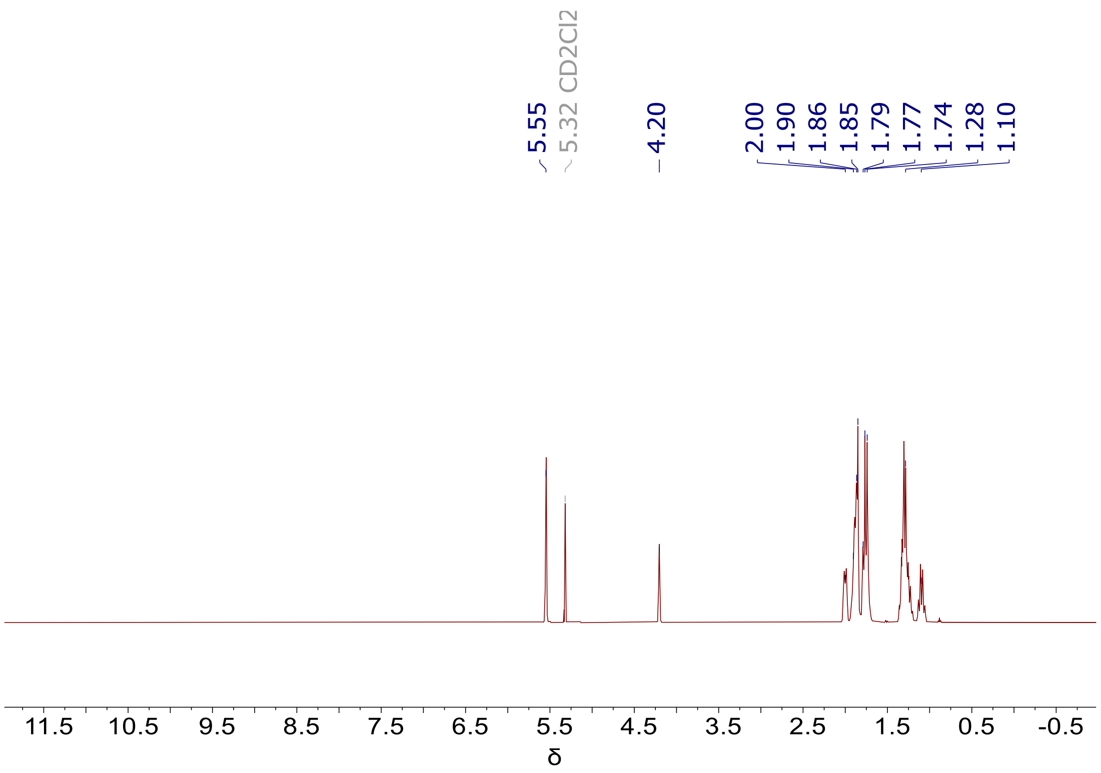

Figure S9. ^{1}H NMR spectrum (CD_2Cl_2, 500 MHz, 298 K) of **[1-NBD][FPB]**.

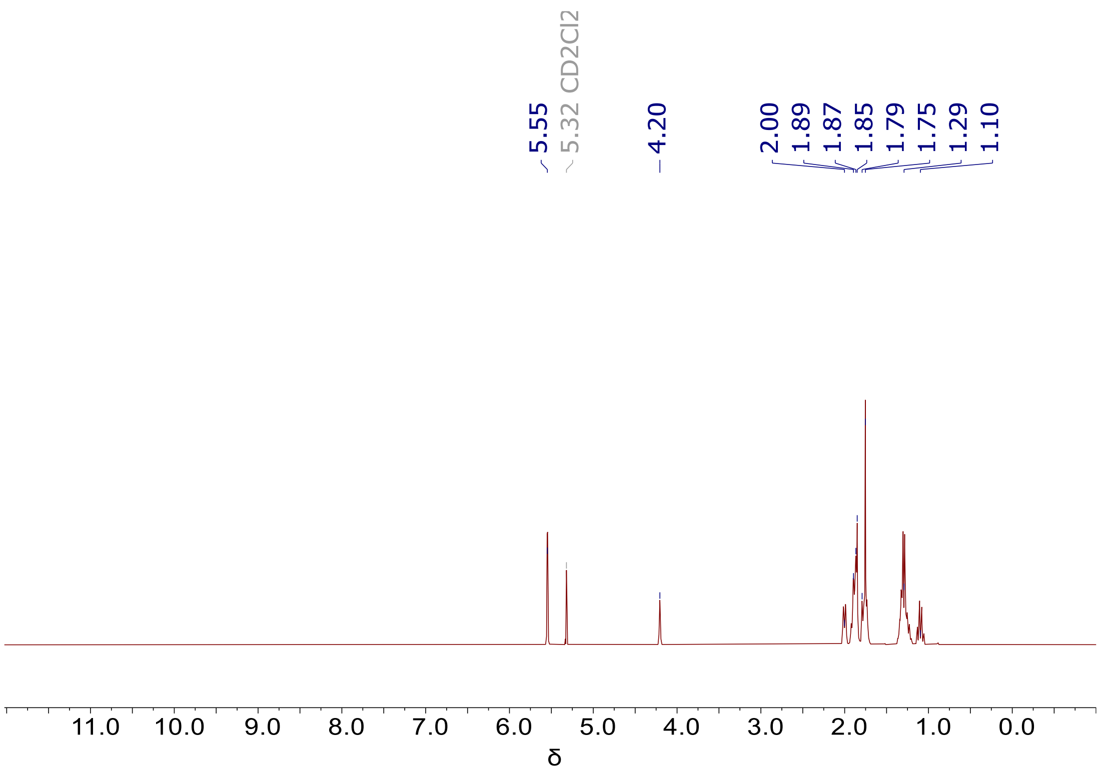

Figure S10. ^{1}H{^{31}P} NMR spectrum (CD_2Cl_2, 500 MHz, 298 K) of **[1-NBD][FPB].**

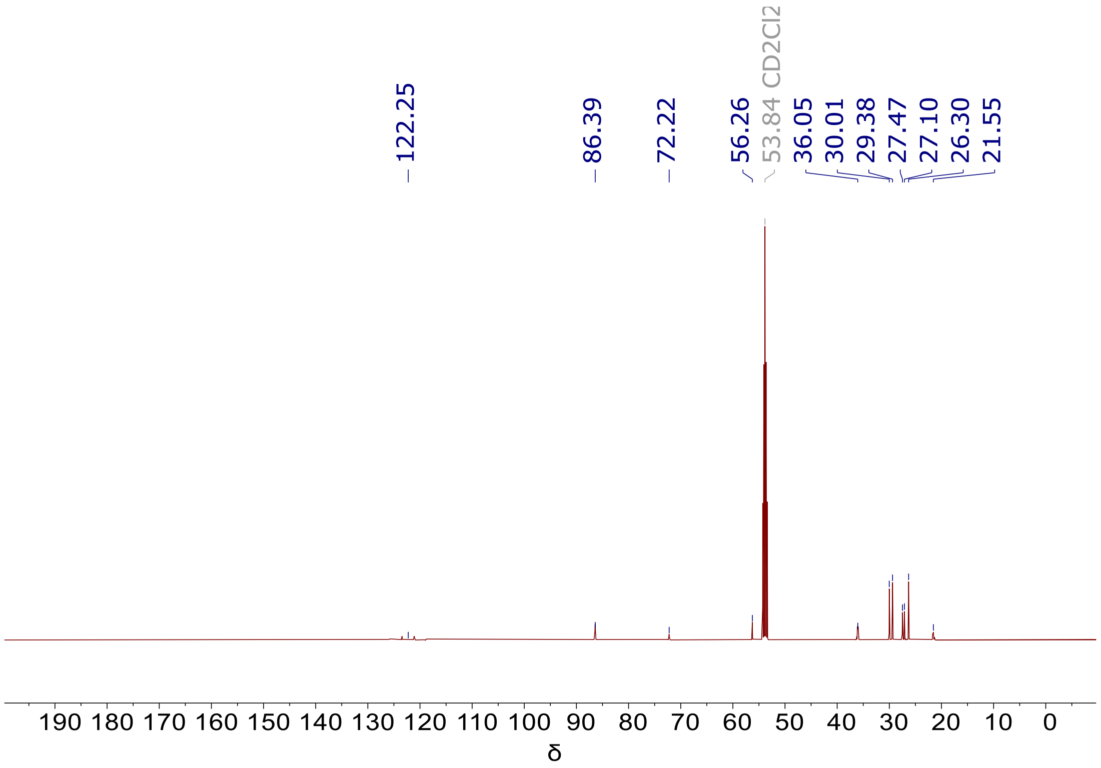

Figure S11. $^{13}C\{^1H\}$ NMR spectrum (CD_2Cl_2, 121 MHz, 298 K) of **[1-NBD][FPB].**

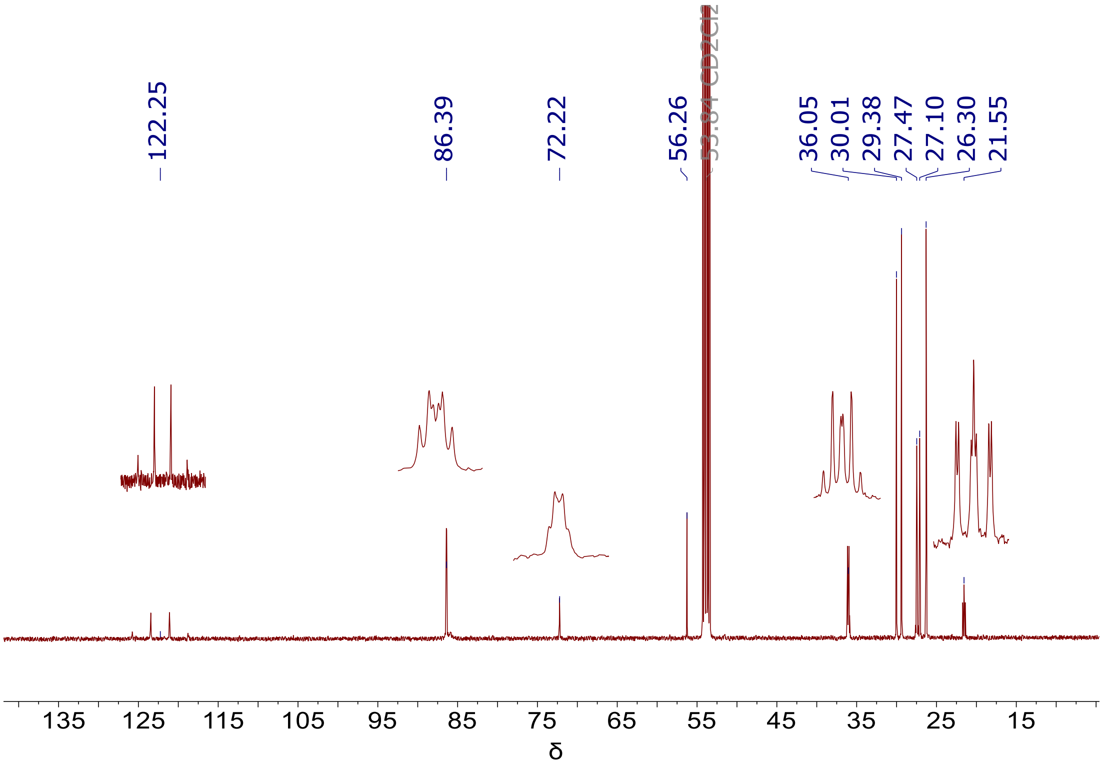

Figure S12. Expanded $^{13}C\{^1H\}$ NMR spectrum (CD_2Cl_2, 121 MHz, 298 K) of **[1-NBD][FPB].**

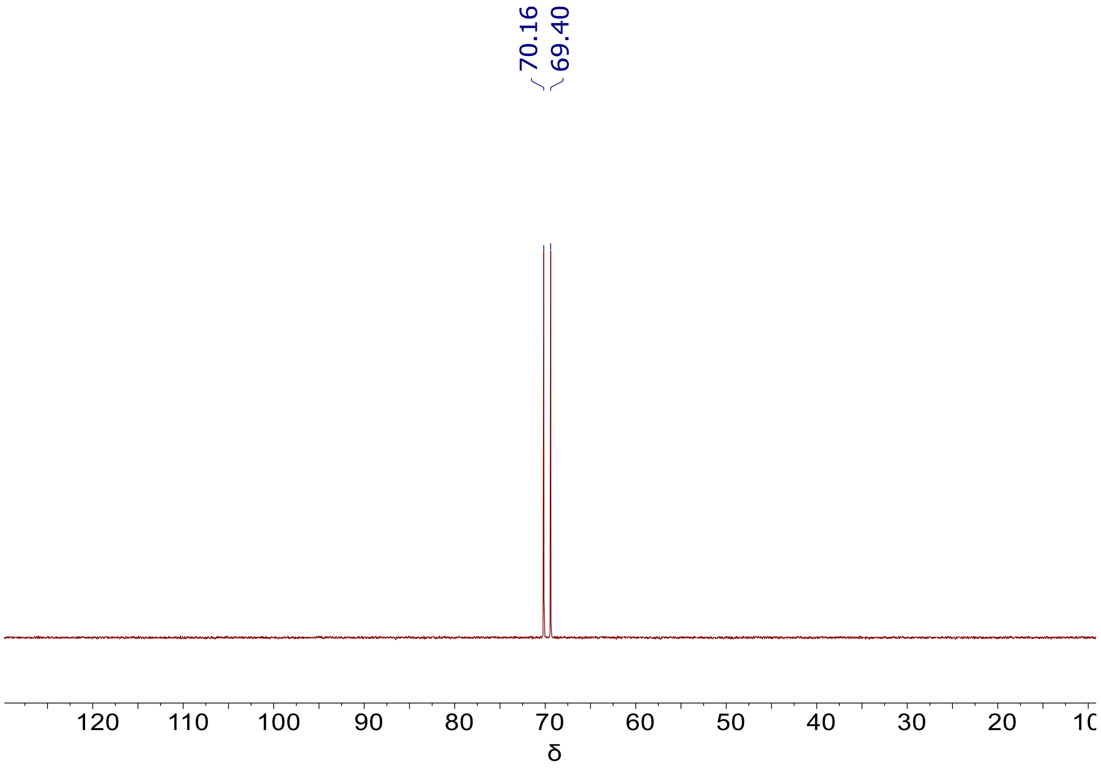

Figure S13. $^{31}P\{^{1}H\}$ NMR spectrum (CD_2Cl_2, 202 MHz, 298 K) of **[1-NBD][FPB]**.

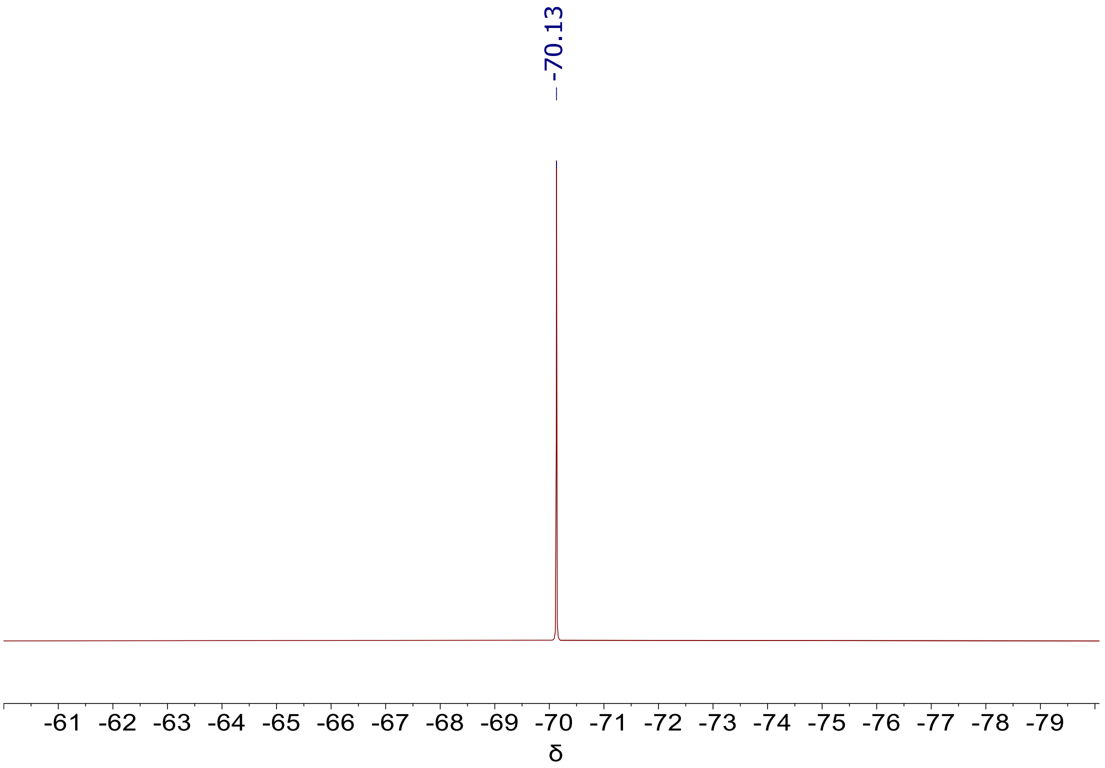

Figure S14. ^{19}F NMR spectrum (CD_2Cl_2, 470 MHz, 198 K) of **[1-NBD][FPB]**.

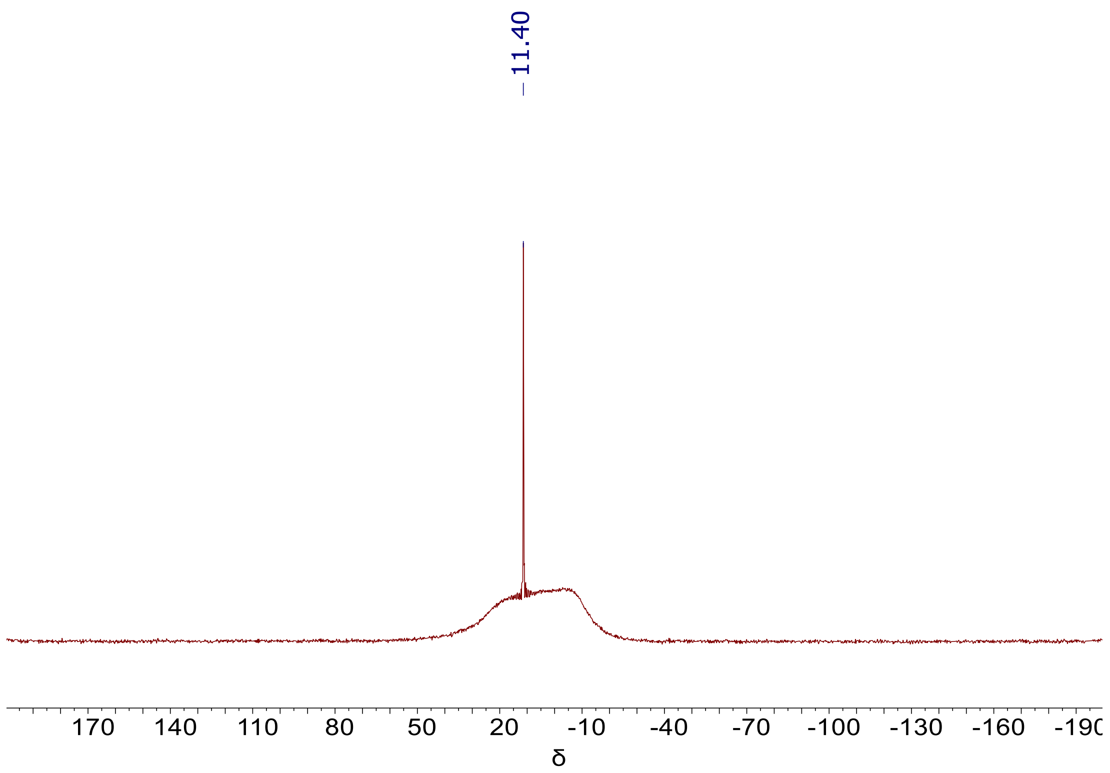

Figure S15. ^{11}B NMR spectrum (CD_2Cl_2, 160 MHz, 298 K) of **[1-NBD][FPB]**.

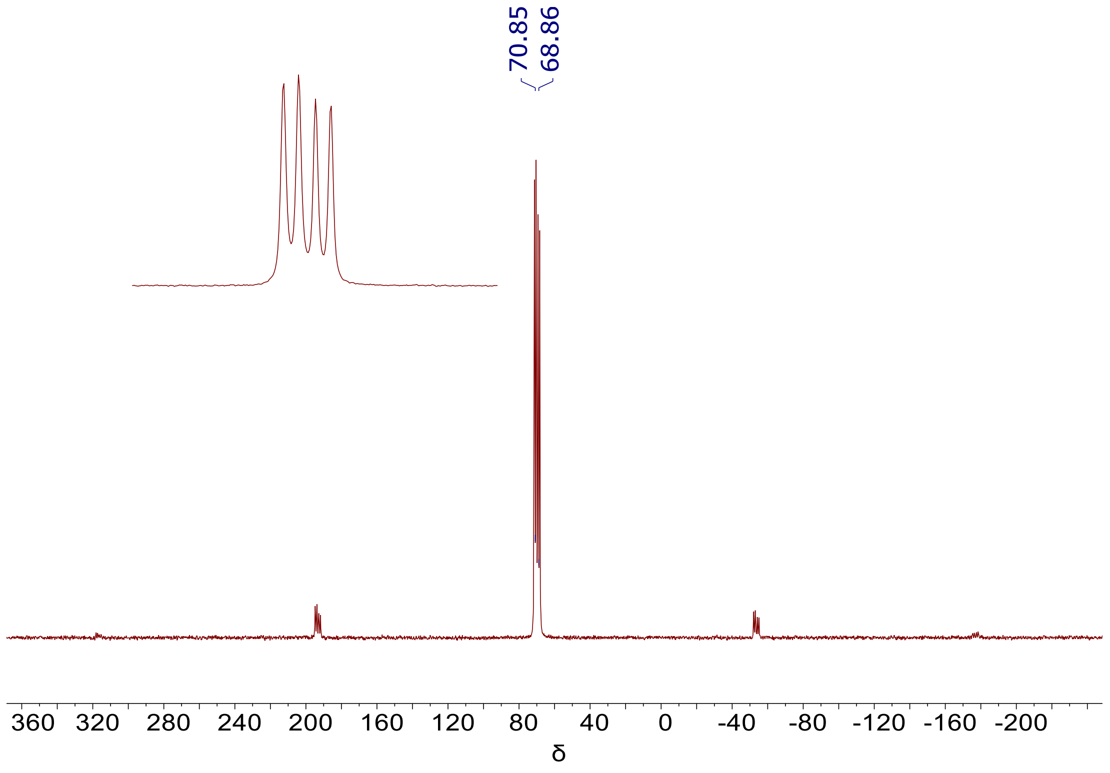

Figure S16. ^{31}P{^{1}H} CPMAS SSNMR spectrum (162 MHz, 20 KHz, 298 K) of **[1-NBD][FPB]**.

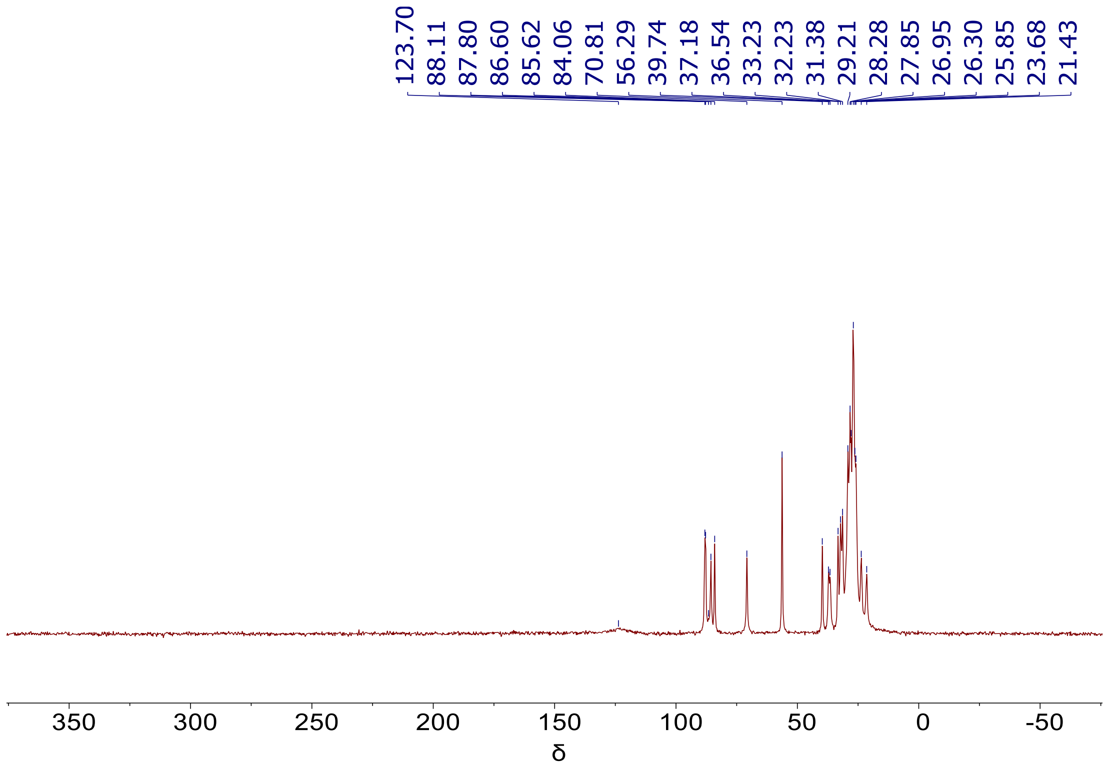

Figure S17. $^{13}C\{^{1}H\}$ CPMAS SSNMR spectrum (100 MHz, 20 KHz, 298 K) of **[1-NBD][FPB]**.

[1-NBA][FPB]

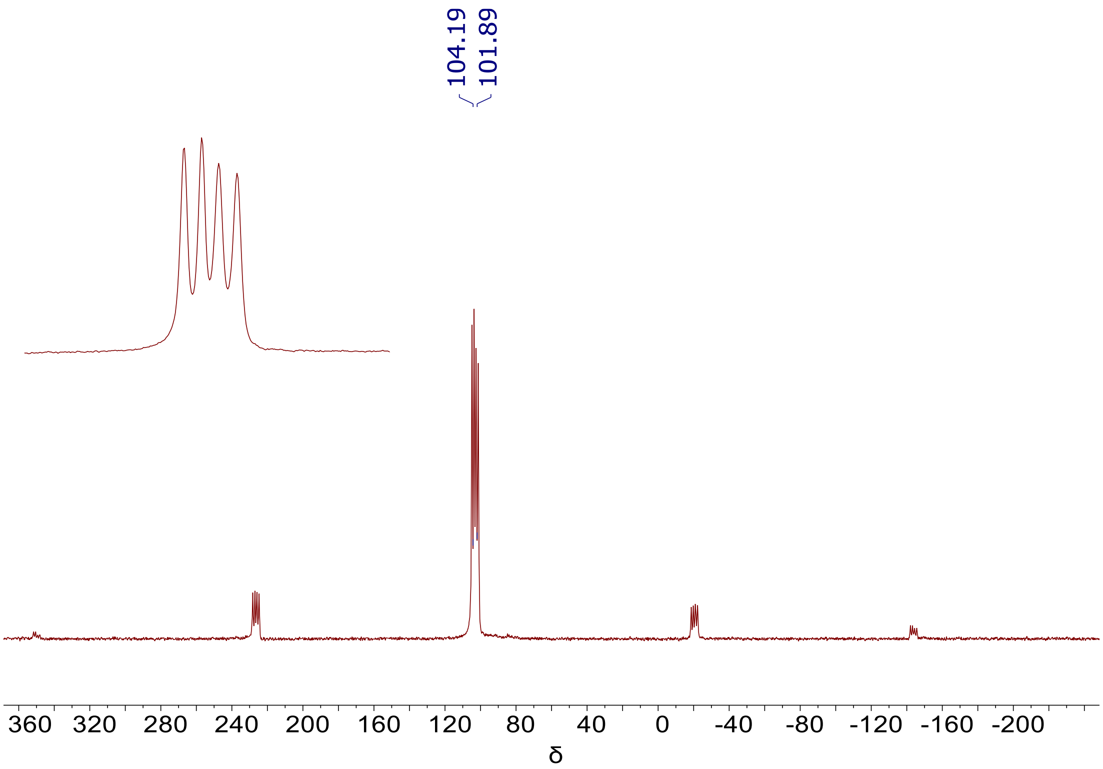

Figure S18. $^{31}P\{^{1}H\}$ CPMAS SSNMR spectrum (162 MHz, 20 KHz, 298 K) of **[1-NBA][FPB]**.

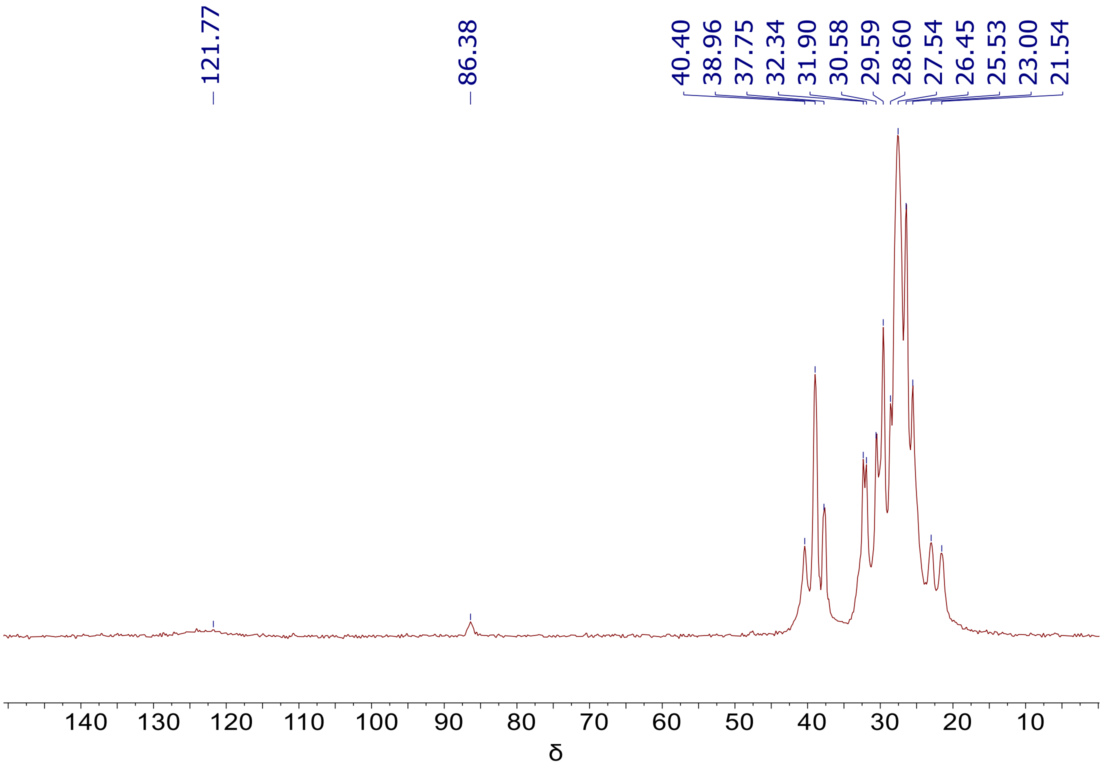

Figure S19. $^{13}C\{^{1}H\}$ CPMAS SSNMR spectrum (162 MHz, 20 KHz, 298 K) of **[1-NBA][FPB]**.

[1-F$_2$C$_6$H$_4$][FPB]

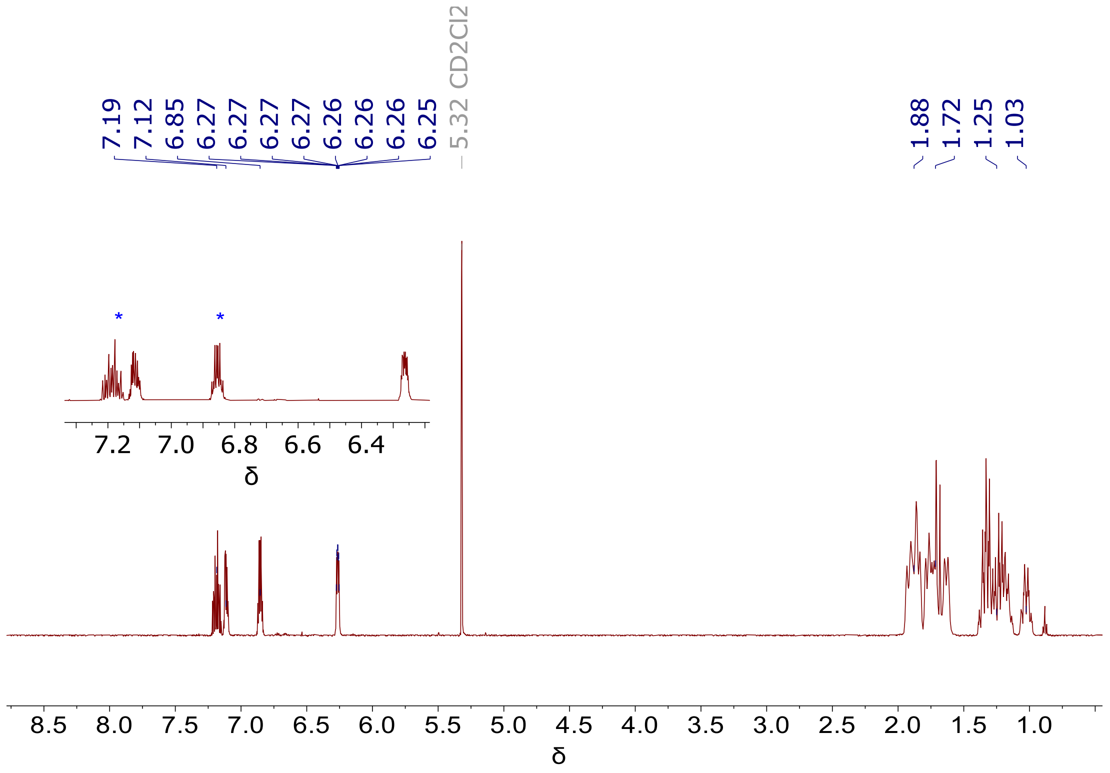

Figure S20. ^{1}H NMR spectrum (CD_2Cl_2, 500 MHz, 298 K) of **[1-F$_2$C$_6$H$_4$][FPB]**. * Denotes one equivalent of 1,2-difluorobenzene solvent of recrystallisation.

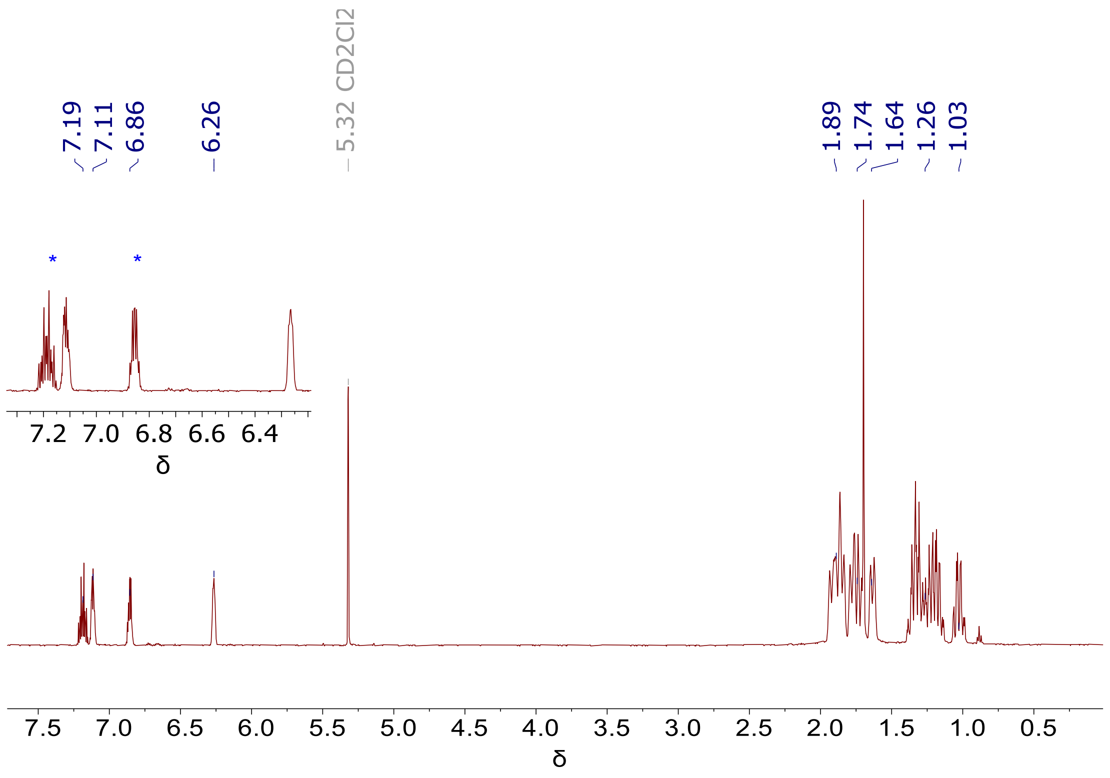

Figure S21. ^{1}H{^{31}P} NMR spectrum (CD_2Cl_2, 500 MHz, 298 K) of **[1-F$_2$C$_6$H$_4$][FPB]**. * Denotes one equivalent of 1,2-difluorobenzene solvent of recrystallisation.

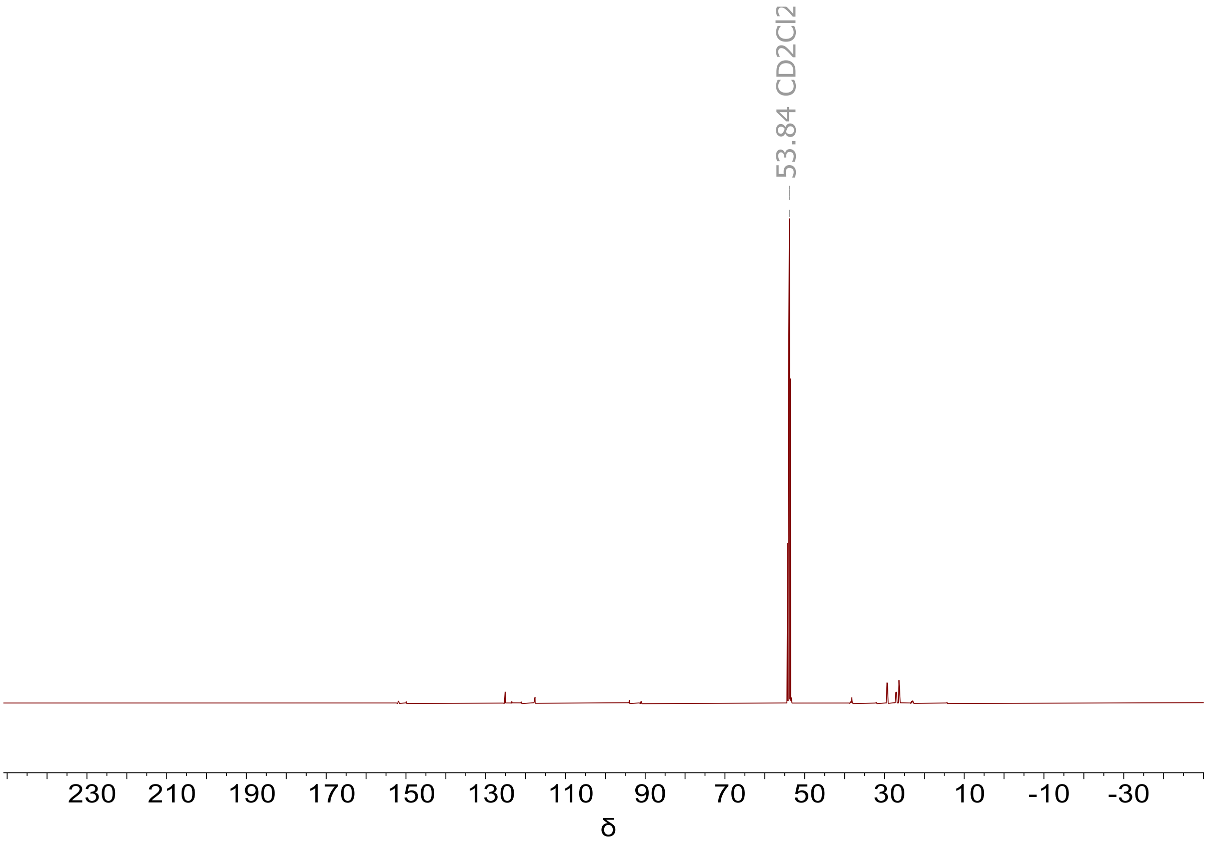

Figure S22. $^{13}C\{^{1}H\}$ NMR spectrum (CD_2Cl_2, 121 MHz, 298 K) of **[1-$F_2C_6H_4$][FPB]**.

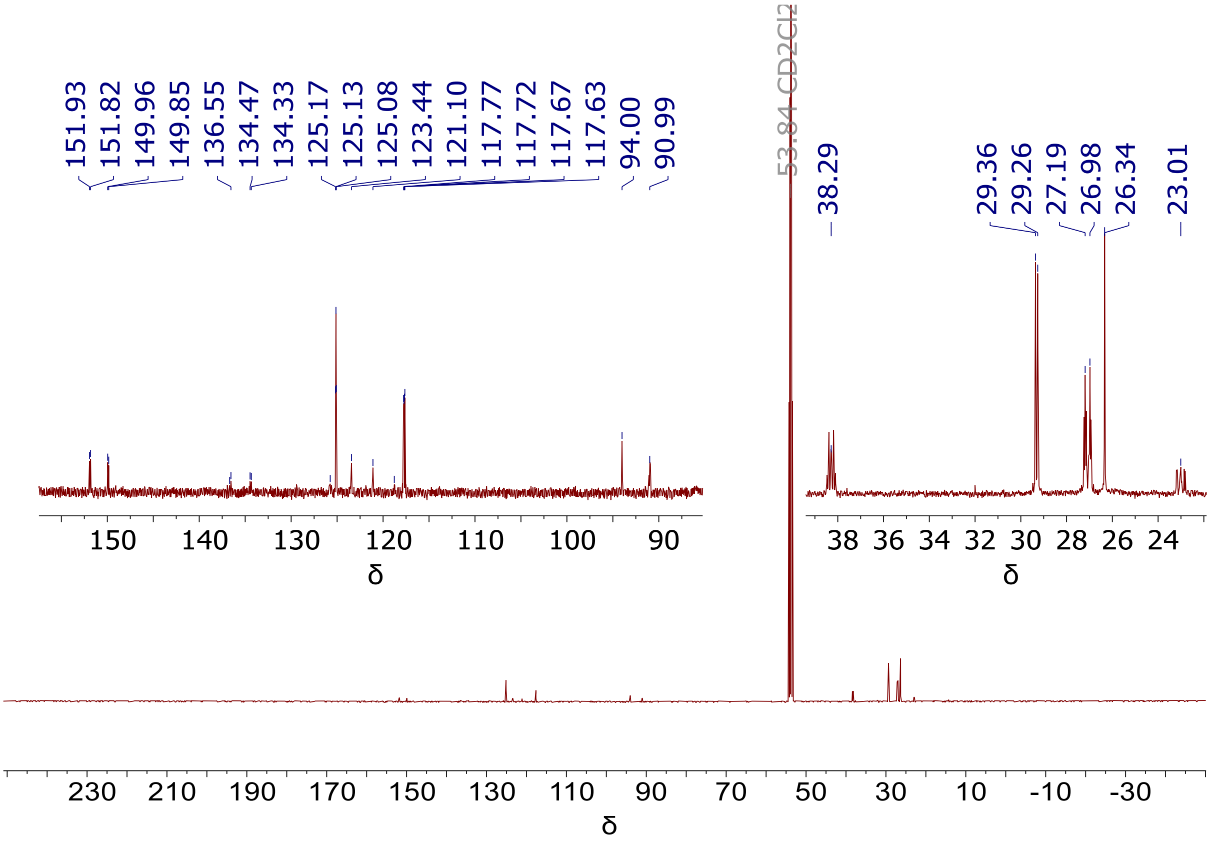

Figure S23. Expanded $^{13}C\{^{1}H\}$ NMR spectrum (CD_2Cl_2, 121 MHz, 298 K) of **[1-$F_2C_6H_4$][FPB]**.

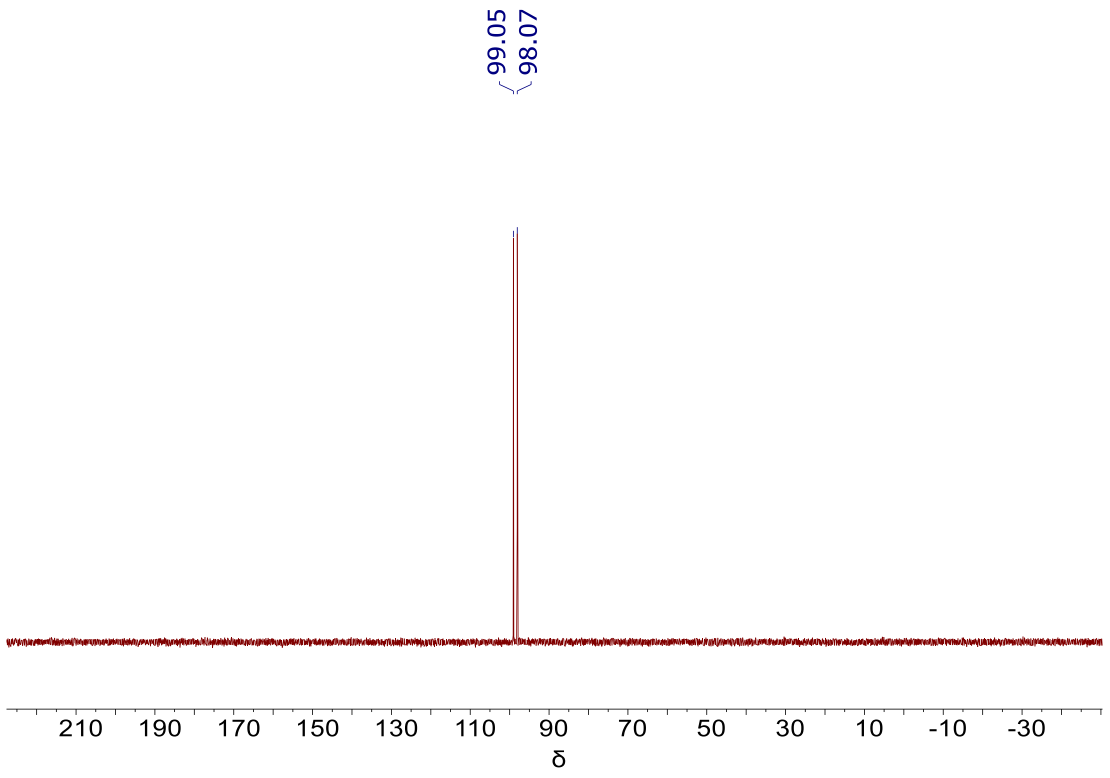

Figure S24. $^{31}P\{^{1}H\}$ NMR spectrum (CD_2Cl_2, 202 MHz, 298 K) of **[1-$F_2C_6H_4$][FPB].**

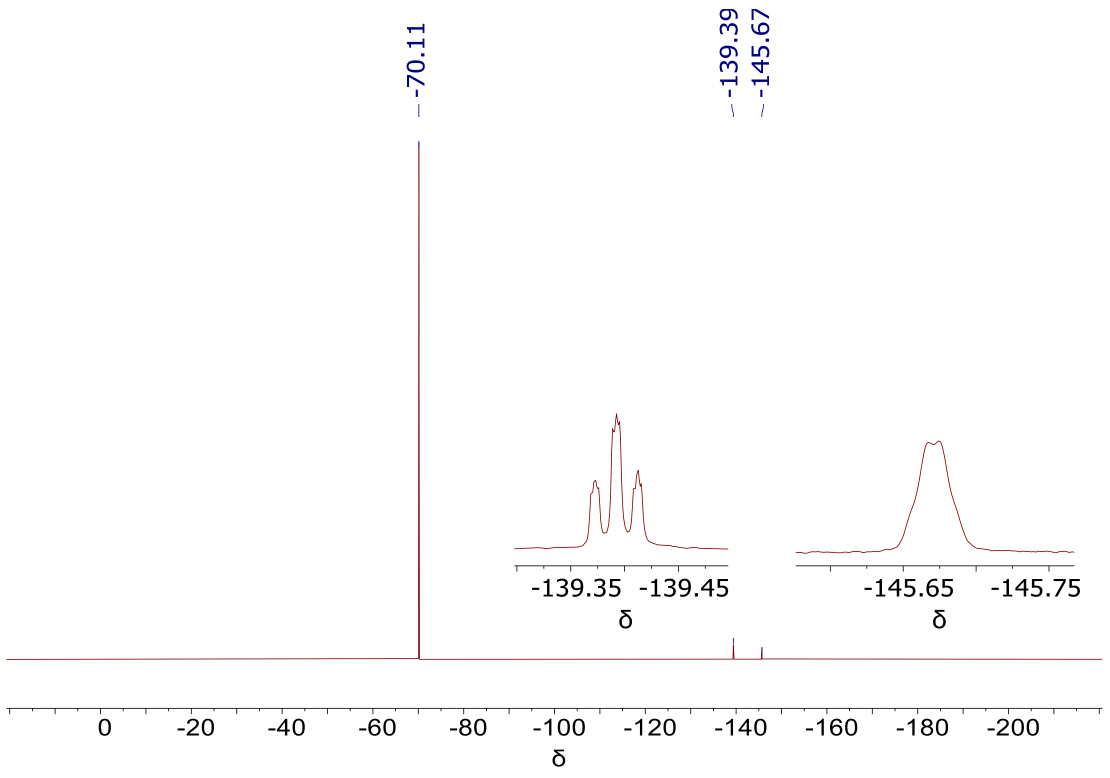

Figure S25. ^{19}F NMR spectrum (CD_2Cl_2, 470 MHz, 198 K) of **[1-$F_2C_6H_4$][FPB]**.

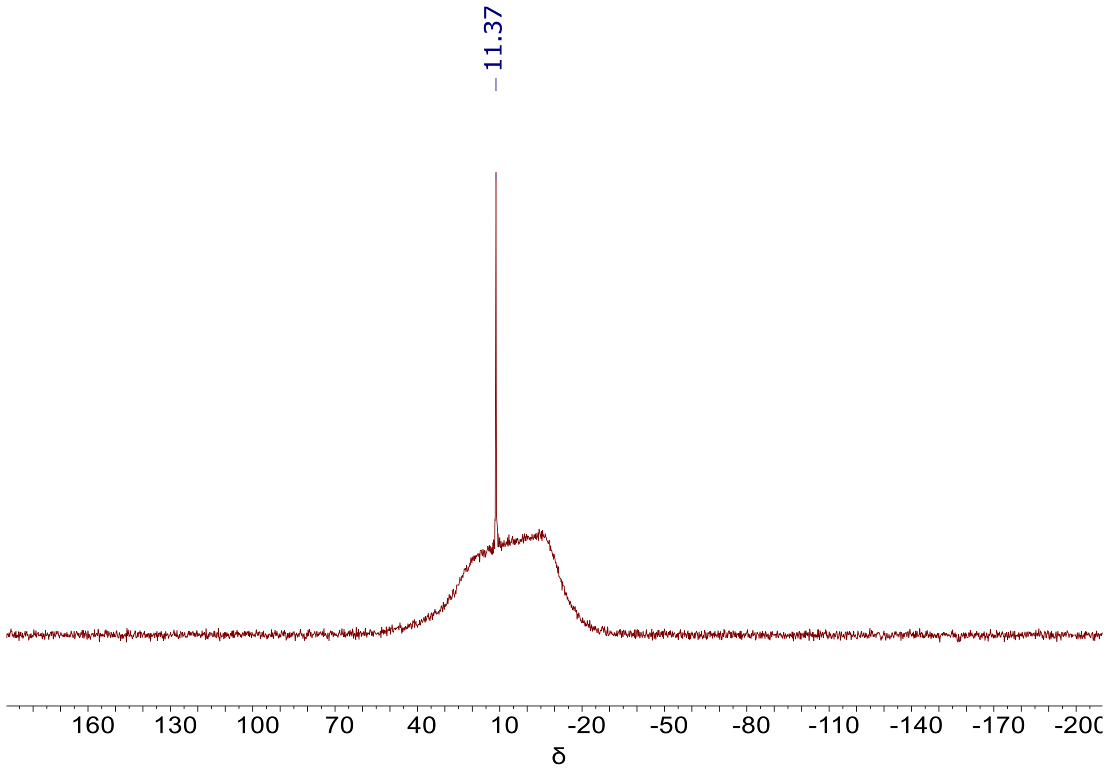

Figure S26. ^{11}B NMR spectrum (CD_2Cl_2, 160 MHz, 298 K) of **[1-$F_2C_6H_4$][FPB].**

S2.0 Additional NMR data

S2.1 Solid-state stability of complex **[1-NBA][FPB]**

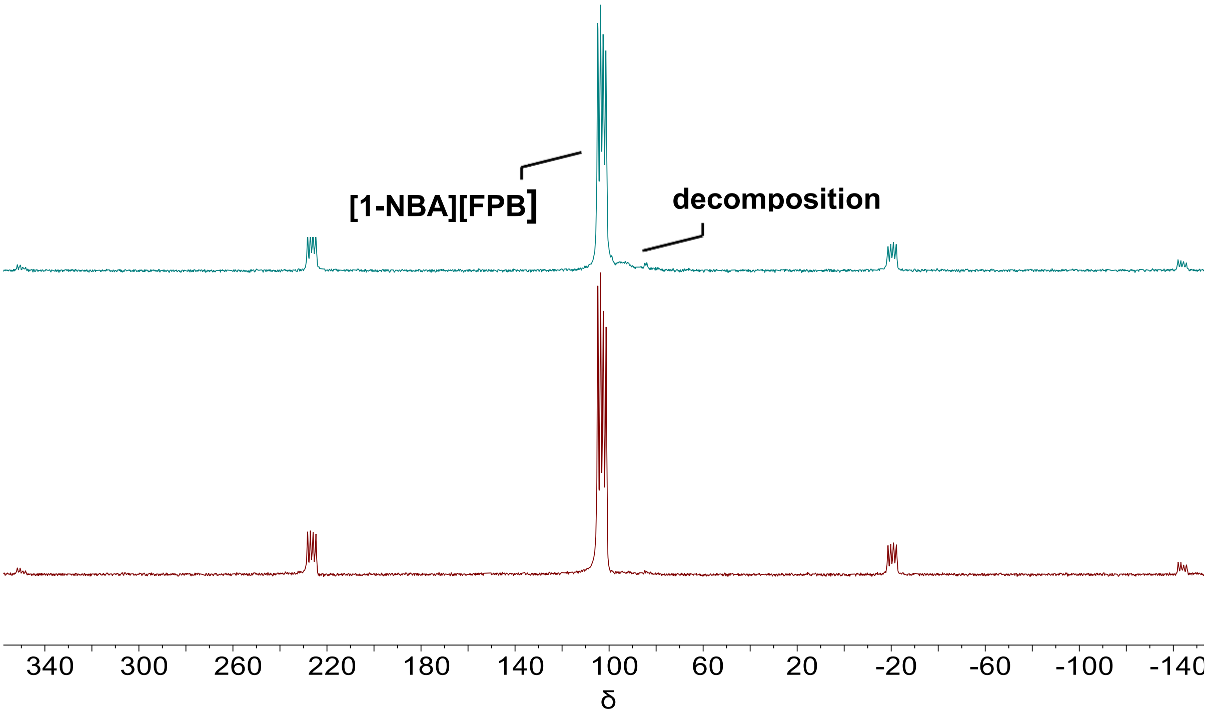

Figure S27. Stacked $^{31}P\{^1H\}$ CPMAS SSNMR spectrum (162 MHz, 20 KHz, 298 K) showing freshly prepared complex **[1-NBA][FPB]** (bottom), and the same sample after one month under argon kept in the same SSNMR rotor (top).

S2.2 Solution-state NMR of [Na(THF)$_n$][FPB] after recrystallisation and post drying at 80 °C.

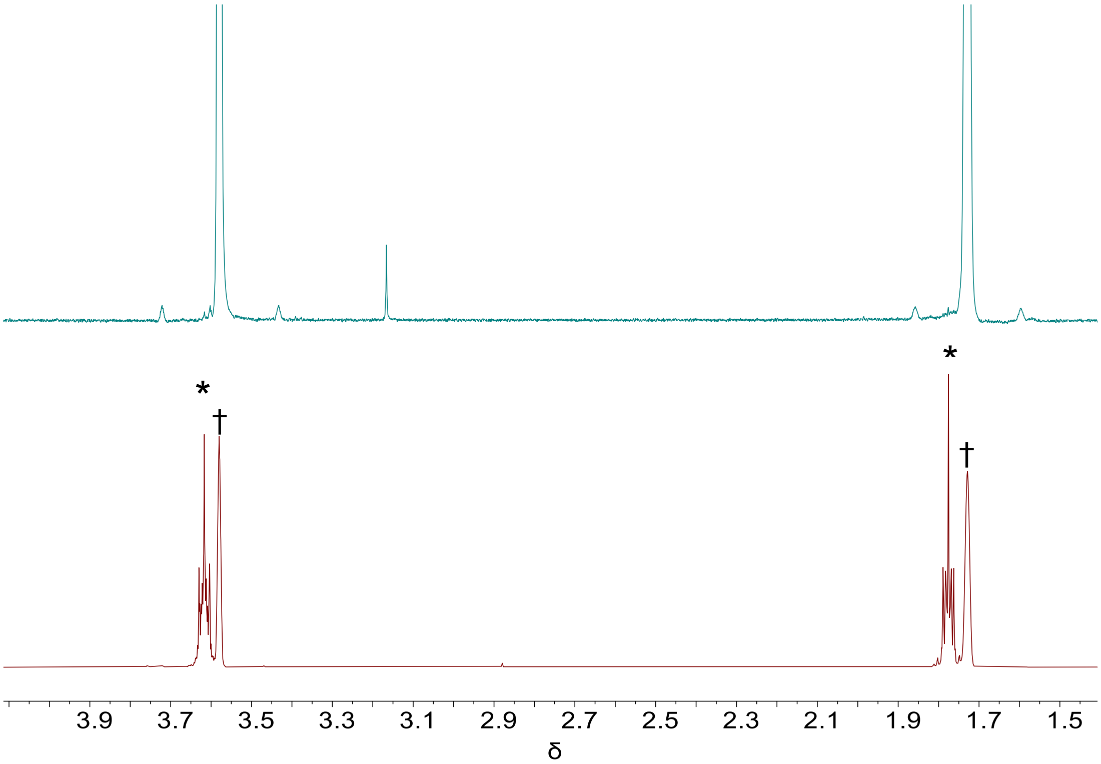

Figure S28. Stacked ^{1}H NMR spectrum (THF-d^8, 500 MHz, 298 K) showing **[Na(THF)$_n$][FPB]** after recrystallisation (bottom) and after drying (top) at 80 °C for 18 hours under vacuum (2 x 10^{-2} mbar) to form the solvate free salt. * Denotes THF-h^8 after displacement by THF-d^8 from **[Na(THF)$_n$][FPB]**. † Denotes THF-d^8 solvent residual peak.

S2.3 Low temperature solution NMR analysis of the product of dissolving complex 3 in dichloromethane-d^2 followed by addition of 1,2-difluorobenzene.

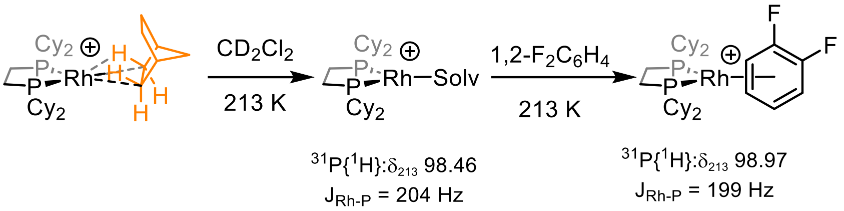

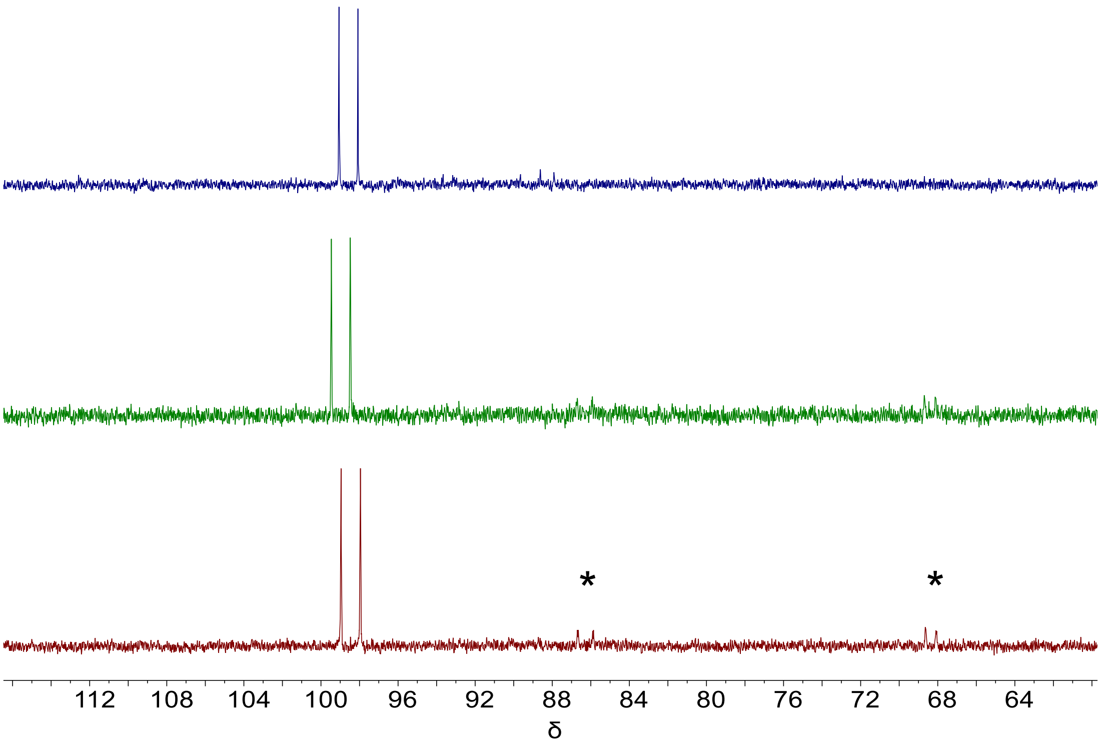

Figure S29. Stacked $^{31}P\{^1H\}$ NMR spectrum (CD_2Cl_2, 202 MHz, 213 – 298 K) of complex **[1-NBA][FPB]** dissolved in CD_2Cl_2 then 1,2,$F_2C_6H_4$ added. Initial $^{31}P\{^1H\}$ NMR spectrum after vacuum transfer of CD_2Cl_2 onto a crystalline sample of complex **3** (bottom, 213 K). $^{31}P\{^1H\}$ NMR spectrum after addition of ~50 μL of 1,2-$F_2C_6H_4$ at 213 K (middle, 213 K) that identifies the formation of complex **[1-F$_2$C$_6$H$_4$][FPB]**. $^{31}P\{^1H\}$ NMR spectrum after warming the sample to 298 K (top, 298K). * Denotes unidentified signals. The identify of the CD_2Cl_2-dissolved complex remains unknown but is likely either a solvent bound complex or anion-bound complex – possibly in fast exchange.

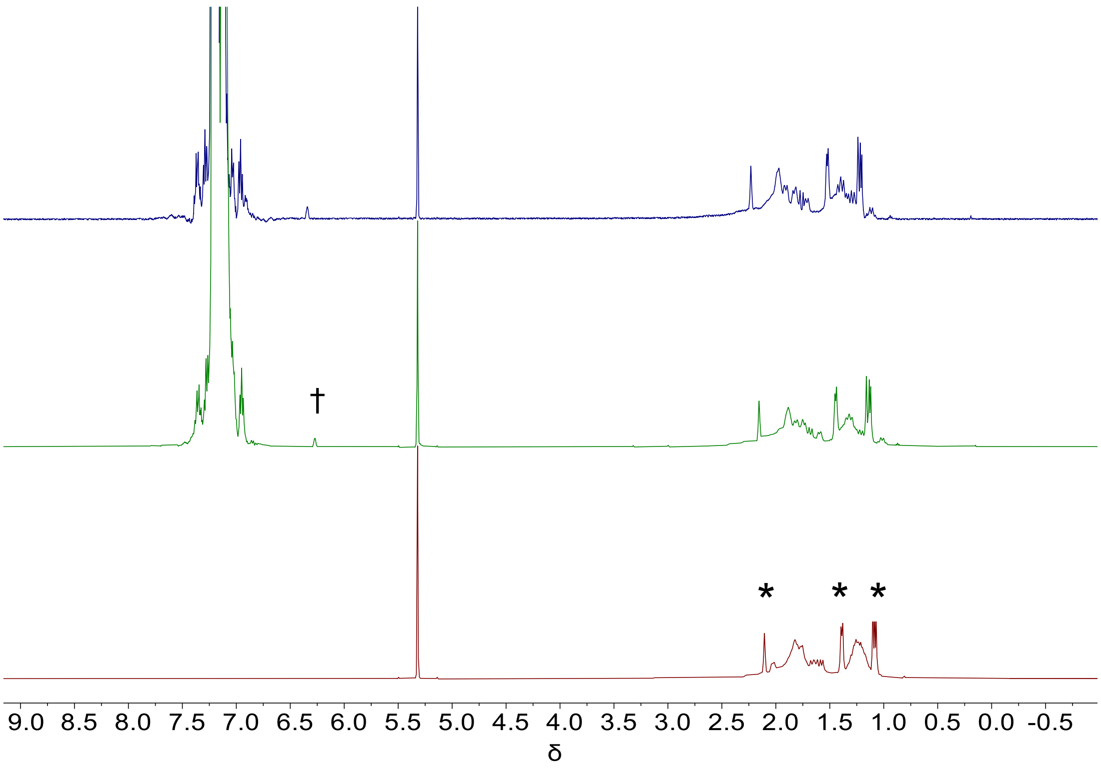

Figure S30. Stacked ^{1}H NMR spectrum (CD_2Cl_2, 500 MHz, 213 – 298 K) of **3** in CD_2Cl_2. Initial ^{1}H NMR spectrum after vacuum transfer of CD_2Cl_2 onto a crystalline sample of **[1-NBA][FPB]** (bottom, 213 K). ^{1}H NMR spectrum after addition of ~50 µL of 1,2-$F_2C_6H_4$ at 213 K (middle, 213 K). ^{1}H NMR spectrum after warming the sample to 298 K (top, 298K). * Denotes norbornane signals, as characterised previously. [3] † Denotes Rh bound 1,2-$F_2C_6H_4$ resonance as found for a pure sample characterised in CD_2Cl_2 (Figure S15). Additional 1,2-$F_2C_6H_4$ resonances are observed between δ 6.84-7.41.

S2.4 Reaction of [H(OEt_2)$_x$][FPB] with proton sponge (PS).

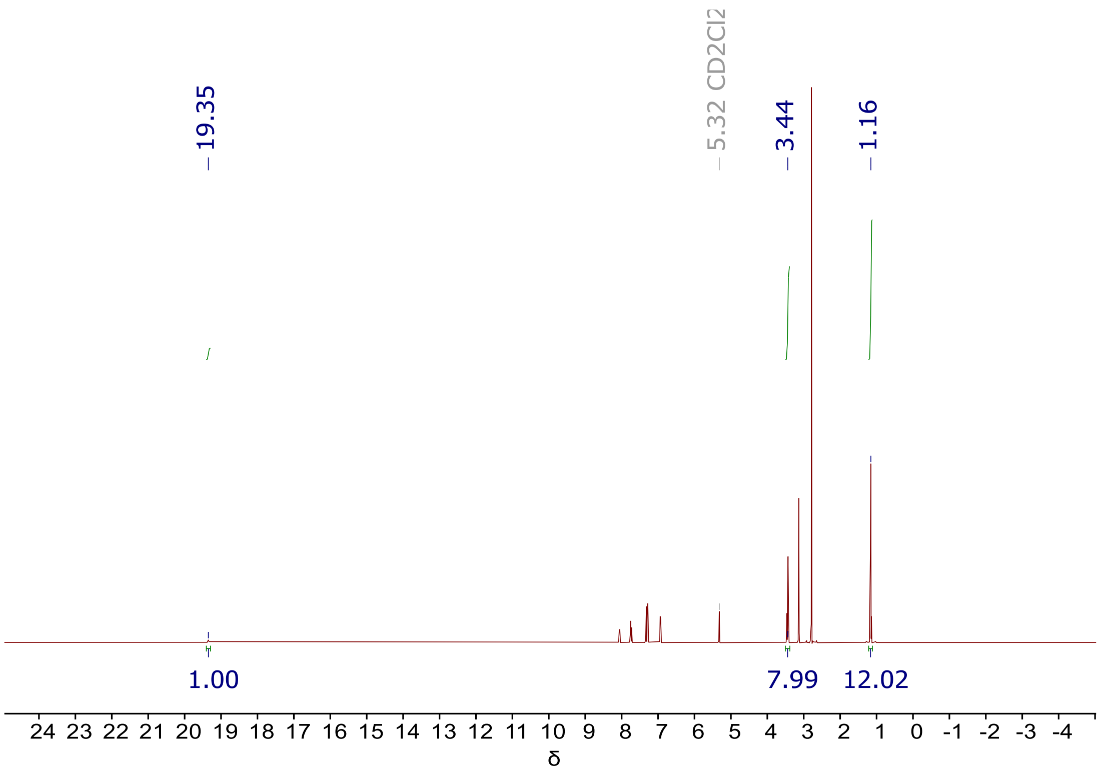

Figure S31. ^{1}H NMR spectrum (CD_2Cl_2, 500 MHz, 298 K) after addition of, as prepared **[H(OEt_2)$_x$][FPB]** to approximately two equivalents of proton sponge (PS) showing formation of [HPS][FPB] by the downfield chemical shift of δ 19.35. Integration of the acidic proton at δ 19.35 with respect to the diethyl ether resonances (δ 3.44 and 1.16) shows a 2:1 ratio of diethyl ether to proton (d1 = 45 s).

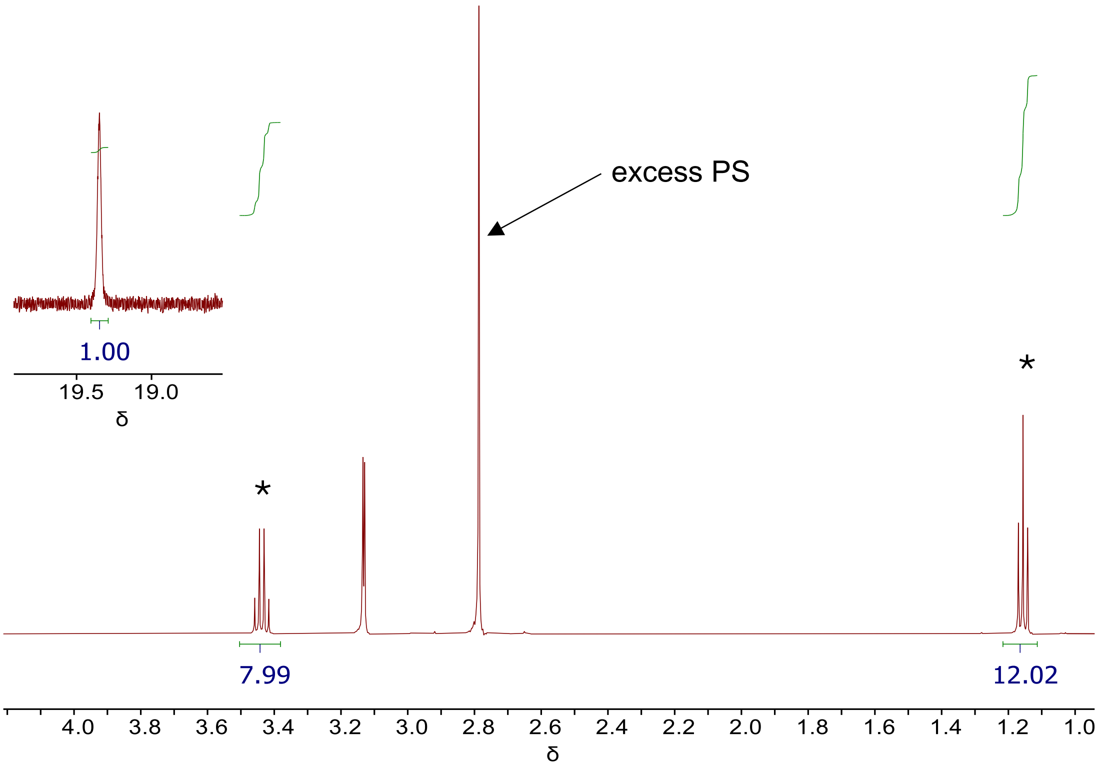

Figure S32. Expanded ^{1}H NMR spectrum (CD_2Cl_2, 500 MHz, 298 K) of the aliphatic region, after addition of, as prepared **[H(OEt_2)$_x$][FPB]** to approximately two equivalents of proton sponge (PS). Inset shows formation of [HPS][FPB] by the downfield chemical shift of δ 19.35. * Indicates diethyl ether.

S3.0 Cost analysis of in-house synthesised Na[BAr^F$_4$] vs Na[FPB]

Table S1. Cost analysis for the in-house synthesis of **Na[FPB]**. PMI = Process Mass Intensity

Na[FPB] scale (mmol):	7.5	Yield (g):	3.06	PMI:	22.6

Reagents (purchased through commercial sources)	Purchase Mass(g)	Purchase Volume (mL)	molecular weight (g/mol)	density (g/ml)	moles	Price (£)	volume used (mL)	mass used (g)	moles used	Price (g^{-1})	Price (mol^{-1})	price per actual amount used	Vendor (Links)	Accessed
Hexafluoro-2,3-bis(trifluoromethyl)-2,3-butanediol	5	-	334.06	1.87	0.015	£56.00		5.000	0.0150	£11.20	£3,741.47	£56.00	Fluorochem	Jan-25
NaBH4	25	-	37.83	-	0.661	£51.10	-	0.1390	0.0037	£2.04	£77.32	£0.28	Sigmaaldrich	Jan-25
Tetrahydrofuran	890	1000	72.11	0.89	12.342	£124.00	35.00	31.15	0.4320	£0.14	£10.05	£4.34	Sigmaaldrich	Jan-25
Hexane	659	1000	86.18	0.659	7.647	£124.00	50.00	32.95	0.3823	£0.19	£16.22	£6.20	Sigmaaldrich	Jan-25
Total								69.24				£66.82		
Total (1 mmol)												£8.91		

Table S2. Cost analysis for the in-house synthesis of Na[BAr$^F{}_4$]. PMI = Process Mass Intensity

NaBAr$^F{}_4$ (mmol)	*23*	*Yield(g):*	*19.6*	*PMI*	*52.9*

Reagents (purchased through commercial sources)	*Purchase Mass(g)*	*Purchase Volume (mL)*	*molecular weight (g/mol)*	*density (g/L)*	*moles*	*Price (£)*	*volume used (mL)*	*mass used (g)*	*moles used*	*Price (g^{-1})*	*Price (mol^{-1})*	*price per actual amount used*	*Vendor (Links)*	*Accessed*
Magnesium turnings	*100*	*-*	*24.31*	*-*	*4.114*	*£27.50*	*-*	*10.00*	*0.411*	*£0.28*	*£6.69*	*£2.75*	*sigmaaldrich*	*Jan-25*
1,3-bis(trifluoromethyl)-5-bromobenzene	*25*	*-*	*293.00*	*1.699*	*0.085*	*£14.00*		*47.57*	*0.162*	*£0.56*	*£164.08*	*£26.64*	*Fluorochem*	*Jan-25*
iodine	*100*	*-*	*253.81*	*-*	*0.394*	*£84.00*	*-*	*1.000*	*0.004*	*£0.84*	*£213.20*	*£0.84*	*Fluorochem*	*Jan-25*
boron trifluoride.diethyletherate	*5.75*	*5*	*141.93*	*1.15*	*0.041*	*£28.90*		*4.600*	*0.032*	*£5.03*	*£713.35*	*£23.12*	*sigmaaldrich*	*Jan-25*
sodium carbonate	*500*	*-*	*105.99*	*-*	*4.717*	*£50.00*	*-*	*80.00*	*0.755*	*£0.10*	*£10.60*	*£8.00*	*sigmaaldrich*	*Jan-25*
dichloromethane	*1325*	*1000*	*84.93*	*1.325*	*15.601*	*£33.10*		*39.75*	*0.468*	*£0.02*	*£2.12*	*£0.99*	*sigmaaldrich*	*Jan-25*
diethyl ether	*706*	*1000*	*74.12*	*0.706*	*9.525*	*£127.00*		*247.1*	*3.334*	*£0.18*	*£13.33*	*£44.45*	*sigmaaldrich*	*Jan-25*
tetrahydrofuran	*890*	*1000*	*72.11*	*0.89*	*12.342*	*£124.00*		*26.70*	*0.370*	*£0.14*	*£10.05*	*£3.72*	*Sigmaaldrich*	*Jan-25*
Water	*-*	*-*	*18*	*1*	*-*	*-*	*500*	*500.0*	*27.778*	*-*	*-*	*-*	*-*	*-*
Magnesium sulfate	*500*	*-*	*120.37*	*-*	*4.154*	*£71.30*	*-*	*80.00*	*0.665*	*£0.14*	*£17.16*	*£11.41*	*sigmaaldrich*	*Jan-25*
Total								*1036.72*			*£1,133.42*	*£121.92*		
Total (1 mmol)												*£5.30*		

S4.0 Computational Details

All static Kohn-Sham DFT calculations were performed on a periodic model of **[(1-NBA)][FPB]** employing the Gaussian Plane Wave (GPW) formalism as implemented in the QUICKSTEP[S4] module within the CP2K program suite (Version 2023.1).[S5] Molecularly optimised basis sets of double-ζ quality plus polarization in their short-range variant (DZVP-MOLOPT-SR-GTH)[S6] were used for all atomic species. The interaction between the core electrons and the valence shell (Rh: 17, B: 3, C: 4 O: 6, P: 5, F: 7, H: 1 electrons) was described by Goedecker-Teter-Hutter (GTH) pseudo potentials.[S7-S9] The generalized gradient approximation (GGA) to the exchange-correlation functional according to Perdew-Burke-Ernzerhof (PBE)[S10] was used in combination with Grimme's D3 correction for dispersion interactions.[S11] The auxiliary plane wave basis set was truncated at a cutoff of 500 Ry. The maximum force convergence criterion was set to 10^{-4} Eh·Bohr^{-1}, whilst default values were used for the remaining criteria. The convergence criterion for the self-consistent field (SCF) accuracy was set to 10^{-7} Eh and 10^{-8} Eh for geometry optimizations. The Brillouin zone was sampled using the Γ-point.

Initial coordinates for **[(1-NBA)][FPB]** were obtained from the experimental crystallographic data from which the positions of the H and F atoms were optimised with all other atomic positions fixed. These partially optimised structures were used in the analysis; calculation of the fully optimised structure of **[(1-NBA)][FPB]** gave only minor differences. Periodic boundary conditions were applied throughout in combination with fixed unit cell parameters obtained from experiment. The endo-bound isomer was modelled with **[1-*endo*-NBA)]$^+$@[(1-NBA)][FPB]**, i.e. where one Rh center is optimized as **[1-*endo*-NBA)]$^+$** within the until cell of **[(1-NBA)][FPB]**. Ion-pair energies were computed with the PBE functional both with and without the D3 correction. The difference gives an estimate of the contribution of dispersion to the ion-pair binding energy.

Independent gradient model calculations were performed with Multiwfn[S12] with the Hirshfeld partitioning scheme (IGMH method).[S13] Surfaces were visualized with VMD[S14] QTAIM (Quantum Theory of Atoms in Molecules)[S15] calculations employed the AIMALL package.[S16] NCI calculations were performed using the NCIPLOT program[S17, S18] and employed promolecular electron densities. Wiberg bond indices and natural atomic charges were computed with the NBO 6.0 program.[S19]

S4.1 Electronic Structure Analyses.

[1-NBA]$^{+}$ Cation

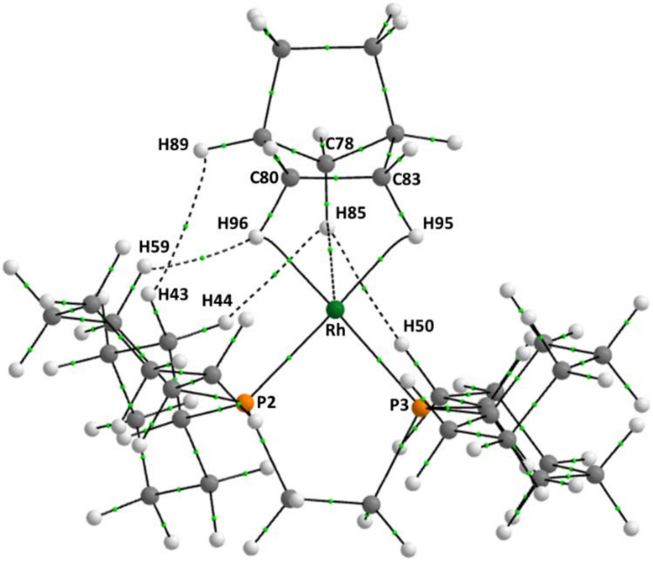

BCP	Distance	ρ	E	$\nabla^2\rho$	H	DI	Atom	q (e)
Rh−H95	1.851	0.0644	1.1088	0.2545	−0.0102	0.2789	Rh	−0.0849
Rh−H96	1.823	0.0689	0.9290	0.2642	−0.0103	0.2871	H95	−0.0255
Rh−P2	2.211	0.1178	0.0037	0.0814	−0.0576	1.0960	H96	−0.0224
Rh−P3	2.209	0.1182	0.0032	0.0834	−0.0580	1.0951	P2	+1.6487
Rh−H85	2.810	0.0125	1.2114	0.0358	0.0006	0.0544	P3	+1.6670
C83−H95	1.166	0.2253	0.0133	−0.5668	−0.1901	0.7567	H85	−0.0040
C80−H96	1.167	0.2249	0.0167	−0.5608	−0.1895	0.7402	C83	−0.0416
C80-C83	1.548	0.2333	0.0247	−0.4765	−0.1794	0.9622	C80	−0.0474
C78−H85	1.100	0.2716	0.0024	−0.9037	−0.2706	0.9039	C78	+0.0116
H96···H59	2.218	0.0078	0.3673	0.0251	0.0013	0.0199	H59	−0.0223
H89···H43	2.594	0.0038	0.2449	0.0124	0.0008	0.0100	H89	+0.0076
H85···H44	2.352	0.0056	0.3620	0.0185	0.0011	0.0154	H43	−0.0180
H85···H50	2.368	0.0052	1.4048	0.0161	0.0010	0.0153	H44	−0.0058
–		–	–	–	–	–	H50	−0.0037

Figure S33. QTAIM molecular graph for **[(1-NBA)]**$^{+}$ with bond critical points (BCP) shown as green spheres. Table shows selected associated BCP parameters (au), distances (Å) and atomic charges. Note the atom labelling used here differs from that in the main text.

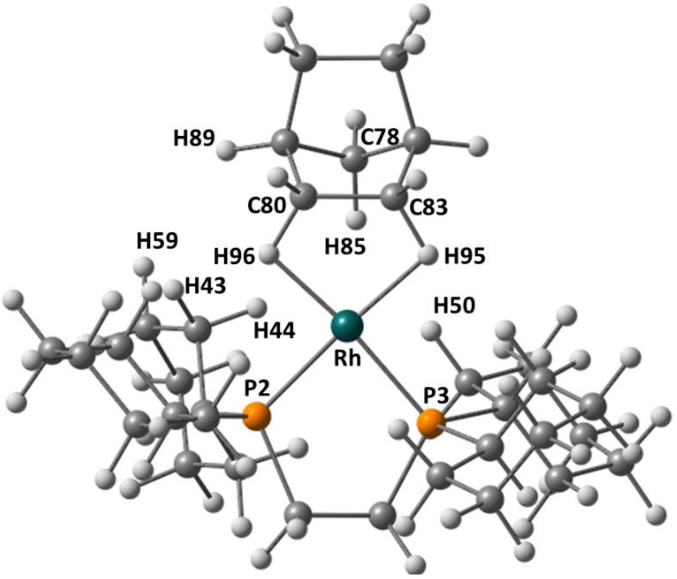

Wiberg bond indices		Natural atomic charges	
Rh−H95	0.2787	Rh	−0.4454
Rh−H96	0.2849	H95	0.2414
Rh−P2	0.6951	H96	0.2399
Rh−P3	0.6953	P2	1.4346
Rh···H85	0.0076	P3	1.4348
C83−H95	0.7786	H85	0.2542
C80−H96	0.7680	C83	−0.4796
C80−C83	1.0102	C80	−0.4826
C78−C85	0.8952	C78	−0.4963
C96···H59	0.0020	H59	0.2391
C89···H43	0.0005	H89	0.2811
C85···H44	0.0015	H43	0.2520
C85···H50	0.0019	H44	0.2525
		H50	0.2612

Figure S34. Structure and tabulated selected Wiberg bond indices and natural atomic charges for **[(1-NBA)]$^+$**.

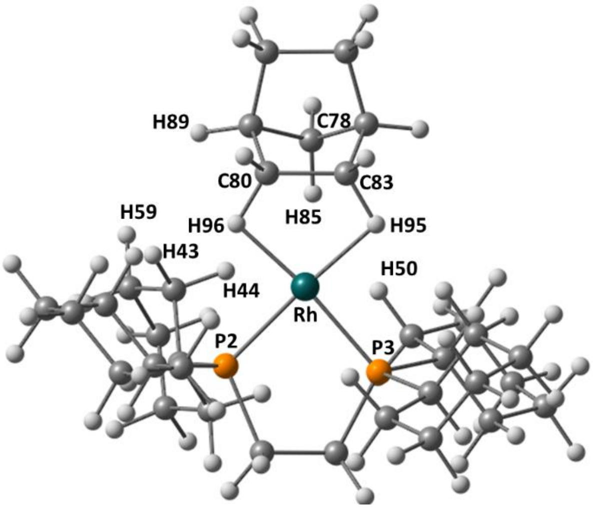

Donor	Acceptor	E(2) kcal/mol
LP(1)Rh	BD*(1)C78-H85	0.67
LP(2)Rh	BD*(1)C83-H95	0.06
LP(3)Rh	BD*(1)C78-H85	0.23
LP(4)Rh	BD*(1)C80-H96	5.85
LP(4)Rh	BD*(1)C83-H95	4.89
BD(1)Rh1-P2	BD*(1)C78-H85	0.36
BD(1)Rh1-P2	BD*(1)C80-H96	4.18
BD(1)Rh1-P2	BD*(1)C83-H95	1.27
BD(1)Rh1-P3	BD*(1)C78-H85	0.36
BD(1)Rh1-P3	BD*(1)C80-H96	0.97
BD(1)Rh1-P3	BD*(1)C83-H95	4.25
BD(1)C78-H85	BD*(1)Rh1-P2	0.26
BD(1)C78-H85	BD*(1)Rh1-P3	0.26
BD(1)C80-H83	BD*(1)Rh1-P2	0.08
BD(1)C80-H83	BD*(1)Rh1-P3	0.14
BD(1)C80-H96	BD*(1)Rh1-P2	0.17
BD(1)C80-H96	BD*(1)Rh1-P3	26.70
BD(1)C83-H95	BD*(1)Rh1-P2	25.87
BD(1)C83-H95	BD*(1)Rh1-P3	0.38

Table S3. Structure and Rh ↔ alkane NBO donor-acceptor interactions in **[(1-NBA)]$^+$** quantified via an NBO 2nd order perturbation analysis.

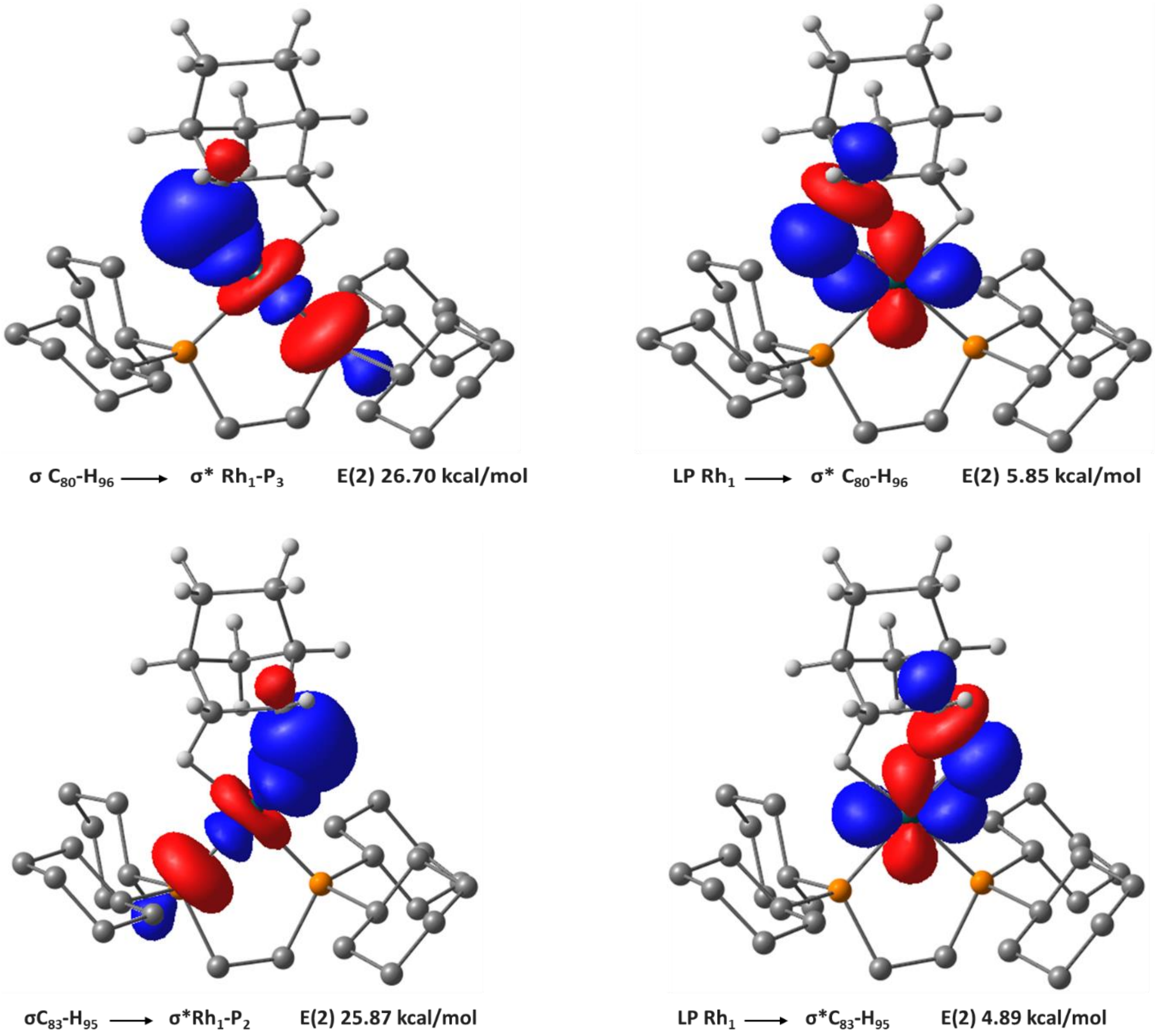

Figure S35. Key Rh ↔ alkane NBO donor-acceptor interactions in **[(1-NBA)]⁺** calculated via the NBO 2nd order perturbation analysis (isosurface value= 0.06 a.u.; cyclohexyl hydrogens omitted for clarity).

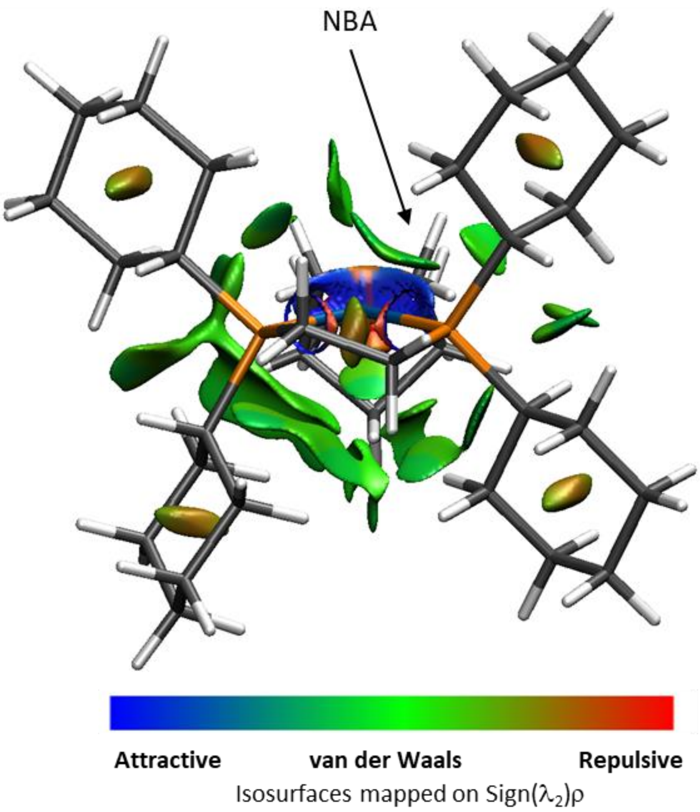

Figure S36. NCIPLOT for **[1-(NBA)]$^+$**. Sign(λ_2)ρ–coloured isosurfaces are plotted with s = 0.30 a.u and −0.07 < ρ < 0.07 a.u.

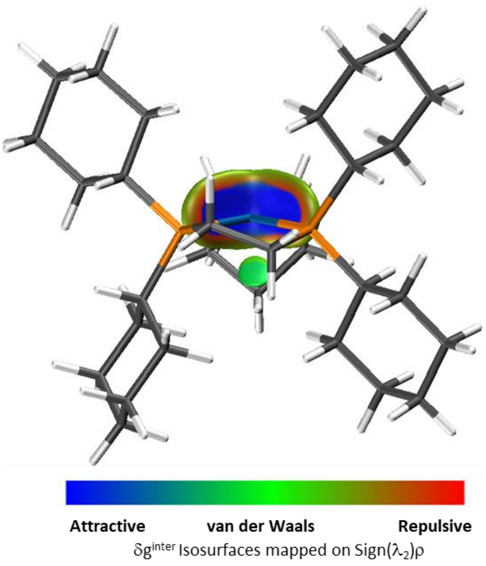

Rh···Atom(B)	δg^{inter}	Distance (Å)
Rh···C80	0.2950	2.363
Rh···C80	0.2811	2.384
Rh···H96	0.2304	1.823
Rh···H95	0.2116	1.852
Rh···C78	0.0643	3.372
Rh···H85	0.0615	2.810

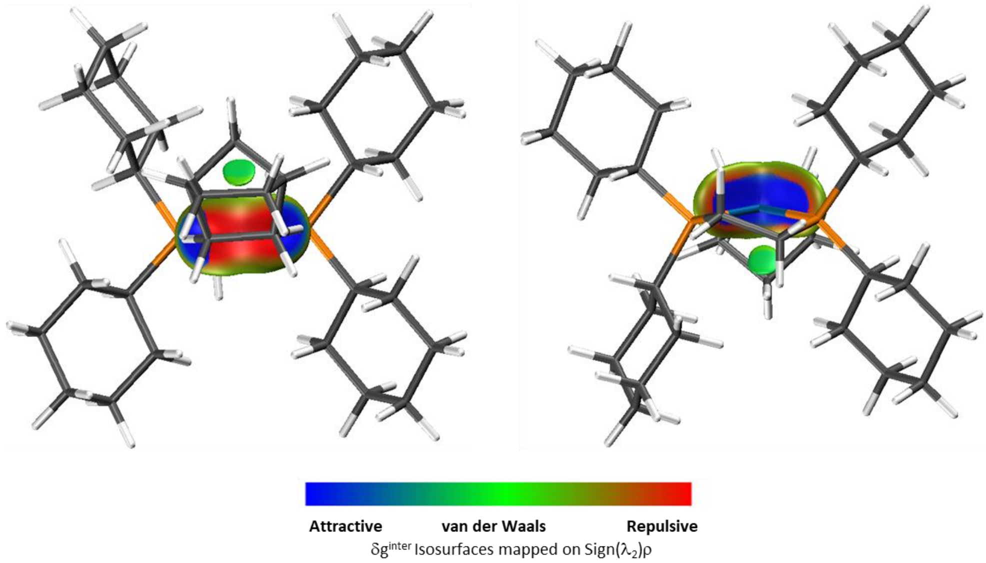

Figure S37. IGMH plots for **[1-NBA]$^+$**. Sign(λ_2)ρ–coloured isosurfaces are plotted with δG^{inter} = 0.01 a.u and 0.003 a.u. respectively. Fragment 1 = Rh ; Fragment 2 = NBA. Table provides δG^{inter} values for specific atom pairs with the equivalent distances.

S4.2 Cation-Anion Ion-pair Interactions

Cation-Anion Ion-pair 1:

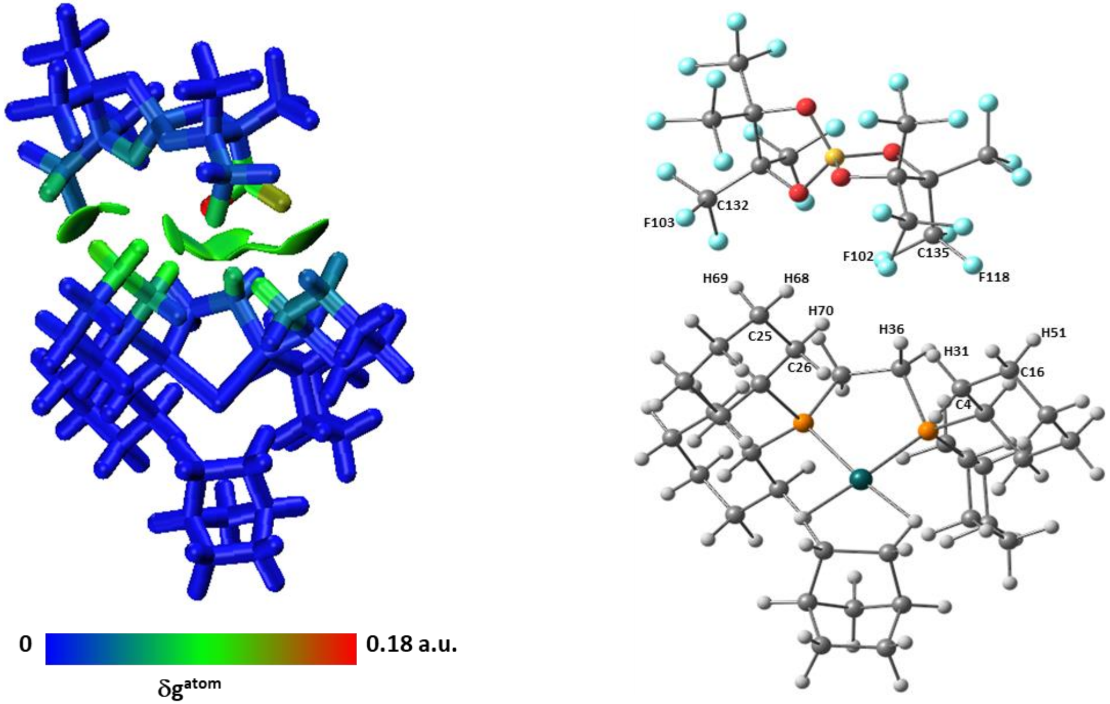

ΔE_{int} = 61.0 kcal/mol
$\Delta E_{dispersion}$ = 8.8 kcal/mol

**Atom(A)···Atom(B)**	**δg^{inter}**	**Distance (Å)**
H70···F102	0.0445	2.239
H69···F103	0.0306	2.388
H31···F118	0.0278	2.523
H36 ···F102	0.0269	2.59
H51···F118	0.0235	2.479
H68···F120	0.0224	2.579
C16···F118	0.0224	3.045
H31···F102	0.0180	2.787
H31···C135	0.0137	3.177
H69···C132	0.0131	3.198
H36···C135	0.0122	3.188

Figure S38. IGMH plot showing interactions between **[1-NBA]⁺** and a neighbouring [FPB]⁻ anion. Cation and anion are defined as separate fragments; sign(λ_2)ρ–coloured isosurfaces are plotted with δG^{inter} = 0.003 a.u; relative atomic contributions coloured by %δg^{atom}. The molecular structure with selected atom labelling is also shown along with tabulated dg^{inter} data and distances for selected atom pairs, ion pair interaction energies (DE_{int}, PBE-D3) and an estimate of the contribution of dispersion (DE dispersion).

Cation-Anion Ion-pair 2:

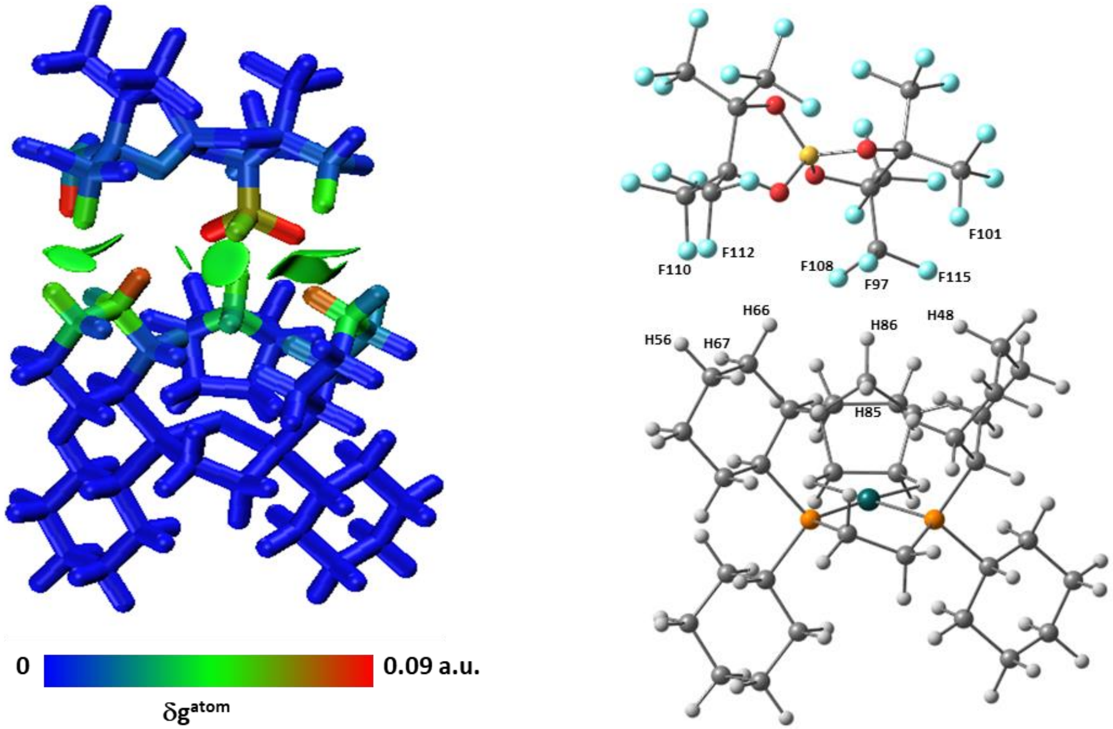

ΔE_{int} = 56.3 kcal/mol
$\Delta E_{dispersion}$ = 7.0 kcal/mol

Atom(A)···Atom(B)	δg^{inter}	Distance (Å)
H86···F108	0.0213	2.668
H56···F112	0.0204	2.638
H48···F101	0.0202	2.704
H66···F110	0.0186	2.704
H56···F110	0.0185	2.715
H48···F115	0.0174	2.776
H48···F97	0.0150	2.786
H48···F97	0.0131	2.916
H67···F110	0.0114	2.895
H65···F115	0.0114	2.854
H66···F97	0.0106	2.863

Figure S39. IGMH plot showing interactions between **[1-NBA]$^+$** and a neighbouring [FPB]$^-$ anion. Cation and anion are defined as separate fragments; sign(λ_2)ρ–coloured isosurfaces are plotted with δG^{inter} = 0.003 a.u; relative atomic contributions coloured by %δg^{atom}. The molecular structure with selected atom labelling is also shown along with tabulated dg^{inter} data and distances for selected atom pairs, ion pair interaction energies (DE_{int}, PBE-D3) and an estimate of the contribution of dispersion (DE dispersion).

Cation-Anion Ion-pair 3:

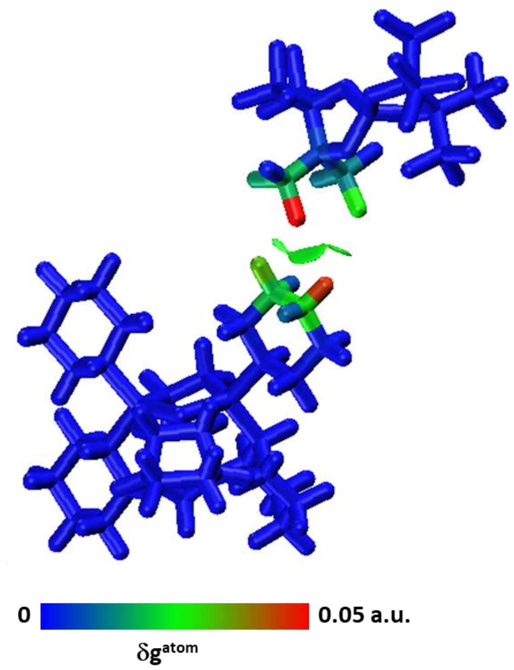

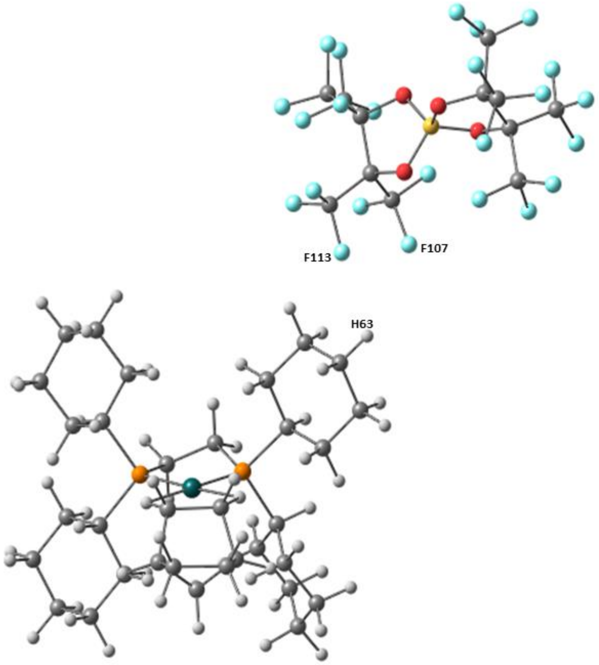

ΔE_{int} = 32.9 kcal/mol
$\Delta E_{dispersion}$ = 1.9 kcal/mol

Atom(A)···Atom(B)	δg^{inter}	Distance (Å)
H63···F113	0.0156	2.773
H63···F107	0.0123	2.798

Figure S40. IGMH plot showing interactions between **[1-NBA]$^+$** and a neighbouring $[FPB]^-$ anion. Cation and anion are defined as separate fragments; sign(λ_2)ρ–coloured isosurfaces are plotted with δG^{inter} = 0.003 a.u; relative atomic contributions coloured by %δg^{atom}. The molecular structure with selected atom labelling is also shown along with tabulated dg^{inter} data and distances for selected atom pairs, ion pair interaction energies (DE_{int}, PBE-D3) and an estimate of the contribution of dispersion (DE dispersion).

Cation-Anion Ion-pair 4:

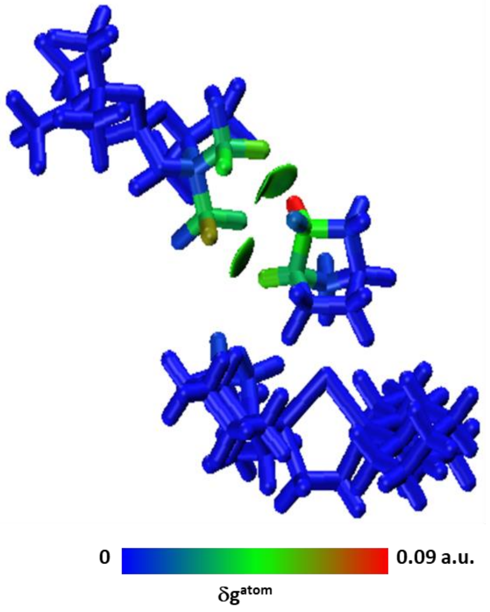

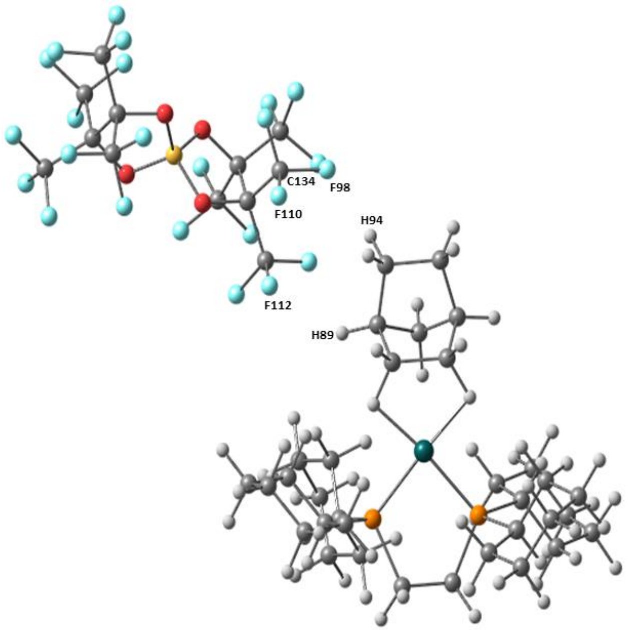

ΔE_{int} = 41.7 kcal/mol
$\Delta E_{dispersion}$ = 3.2 kcal/mol

**Atom(A)···Atom(B)**	**δg^{inter}**	**Distance (Å)**
H94···F98	0.0295	2.418
H89···F112	0.0256	2.493
H94···F110	0.0167	2.780
H94···C134	0.0126	3.162

Figure S41. IGMH plot showing interactions between **[1-NBA]⁺** and a neighbouring [FPB]⁻ anion. Cation and anion are defined as separate fragments; sign(λ_2)ρ–coloured isosurfaces are plotted with δG^{inter} = 0.003 a.u; relative atomic contributions coloured by %δg^{atom}. The molecular structure with selected atom labelling is also shown along with tabulated dg^{inter} data and distances for selected atom pairs, ion pair interaction energies (DE_{int}, PBE-D3) and an estimate of the contribution of dispersion (DE dispersion).

Cation-Anion Ion-pair 5:

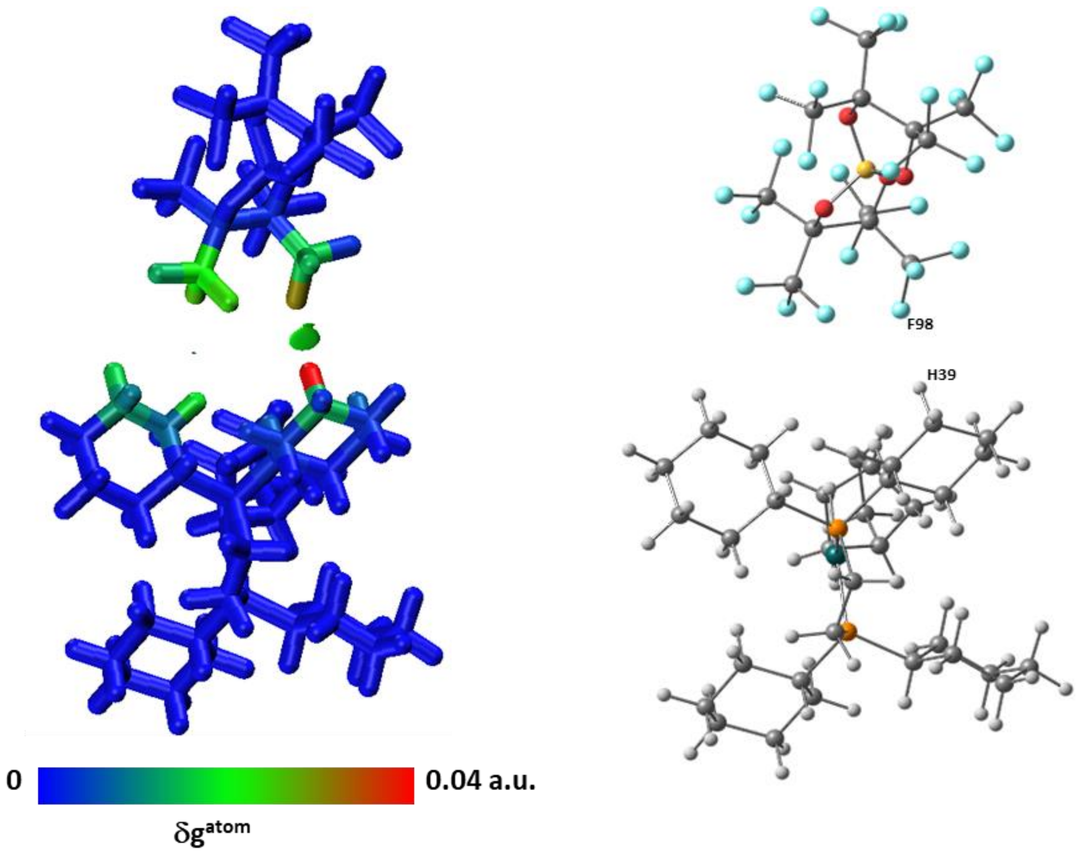

ΔE_{int} = 40.4 kcal/mol
$\Delta E_{dispersion}$ = 3.0 kcal/mol

Atom(A)···Atom(B)	δg^{inter}	Distance (Å)
H39···F98	0.0121	2.828

Figure S42. IGMH plot showing interactions between **[1-NBA]⁺** and a neighbouring [FPB]⁻ anion. Cation and anion are defined as separate fragments; sign(λ_2)ρ–coloured isosurfaces are plotted with δG^{inter} = 0.003 a.u; relative atomic contributions coloured by %δg^{atom}. The molecular structure with selected atom labelling is also shown along with tabulated dg^{inter} data and distances for selected atom pairs, ion pair interaction energies (DE_{int}, PBE-D3) and an estimate of the contribution of dispersion (DE dispersion).

Cation-Anion Ion-pair 6:

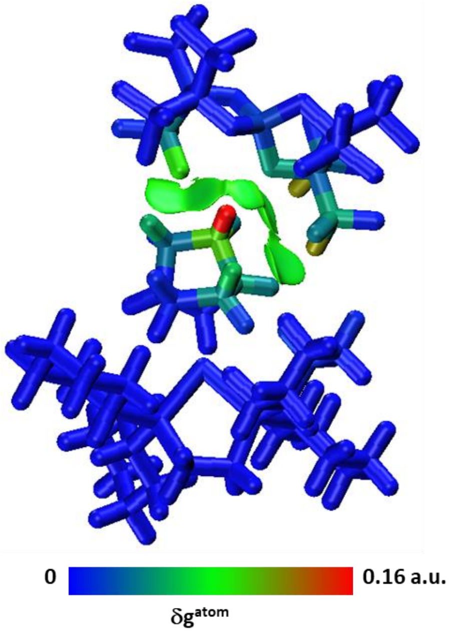

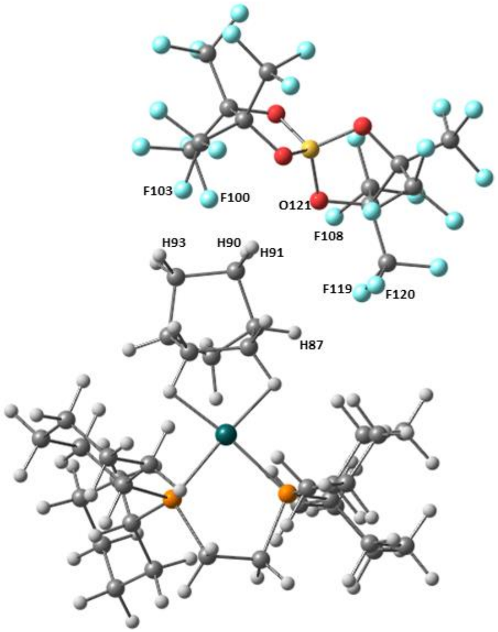

ΔE_{int} = 57.0 kcal/mol
$\Delta E_{dispersion}$ = 6.6 kcal/mol

Atom(A)···Atom(B)	δg^{inter}	Distance (Å)
H91···F108	0.0358	2.385
H91···F100	0.0250	2.606
H93···F100	0.0206	2.681
H90···F108	0.0203	2.631
H91···O121	0.0198	2.819
H87···F119	0.0192	2.669
H92···F119	0.0188	2.720

Figure S43. IGMH plot showing interactions between **[1-NBA]⁺** and a neighbouring [FPB]⁻ anion. Cation and anion are defined as separate fragments; sign(λ_2)ρ–coloured isosurfaces are plotted with δG^{inter} = 0.003 a.u; relative atomic contributions coloured by %δg^{atom}. The molecular structure with selected atom labelling is also shown along with tabulated dg^{inter} data and distances for selected atom pairs, ion pair interaction energies (DE_{int}, PBE-D3) and an estimate of the contribution of dispersion (DE dispersion).

Cation-Anion Ion-pair 7:

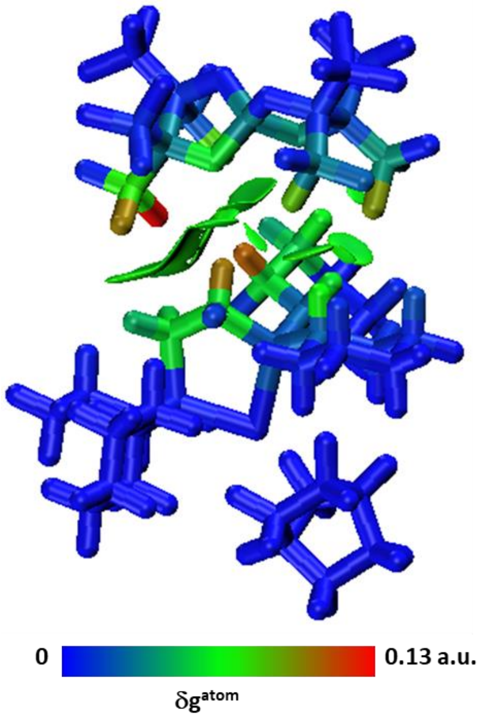

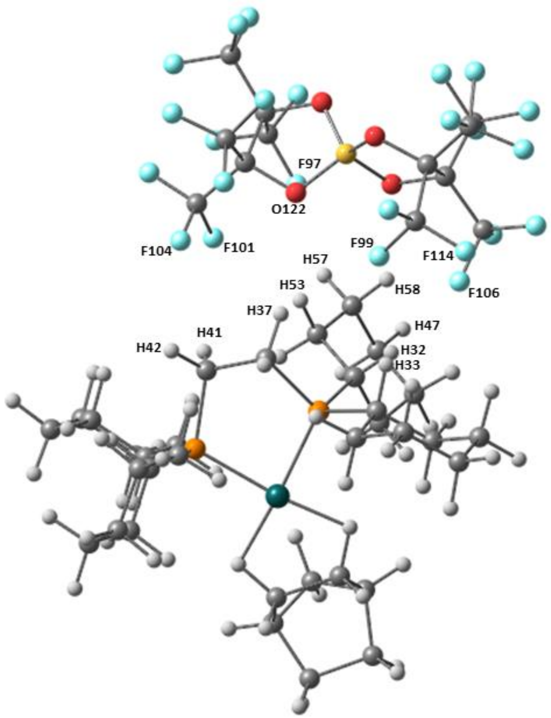

ΔE_{int} = 68.4 kcal/mol
$\Delta E_{dispersion}$ = 9.8 kcal/mol

Atom(A)···Atom(B)	δg^{inter}	Distance (Å)
H53···F101	0.0281	2.485
H33···F99	0.0261	2.525
H32···F106	0.0256	2.534
H42···F104	0.0224	2.560
H41···F101	0.0213	2.677
H57···F97	0.0202	2.599
H37···O122	0.0177	2.835
H53···F97	0.0156	2.853
H37···F104	0.0143	2.877
H33···F106	0.0110	2.919
H37···F99	0.0103	2.891

Figure S44. IGMH plot showing interactions between **[1-NBA]⁺** and a neighbouring [FPB]⁻ anion. Cation and anion are defined as separate fragments; sign(λ_2)ρ–coloured isosurfaces are plotted with δG^{inter} = 0.003 a.u; relative atomic contributions coloured by %δg^{atom}. The molecular structure with selected atom labelling is also shown along with tabulated dg^{inter} data and distances for selected atom pairs, ion pair interaction energies (DE_{int}, PBE-D3) and an estimate of the contribution of dispersion (DE dispersion).

Cation-Anion Ion-pair 8:

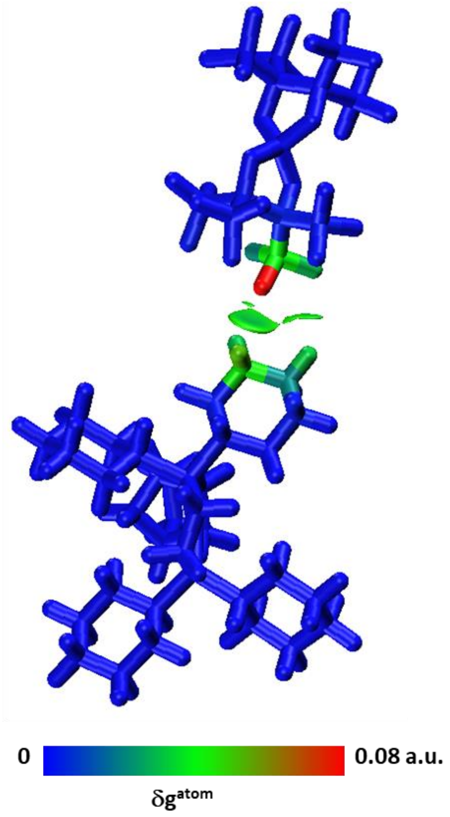

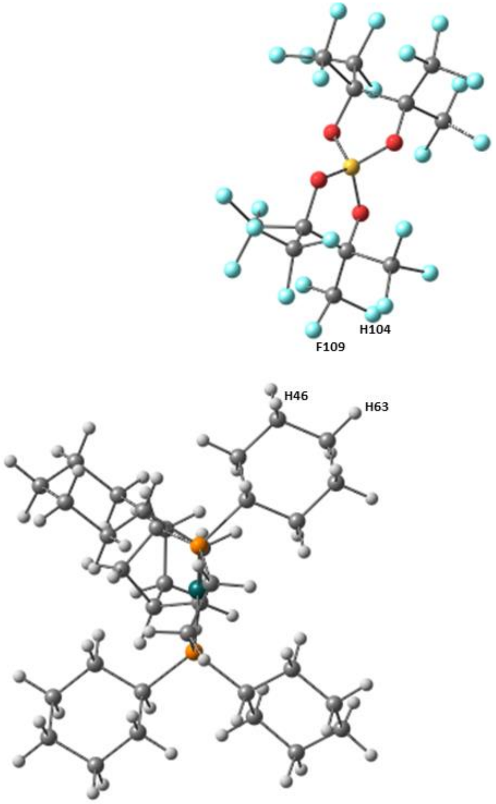

ΔE_{int} = 32.7 kcal/mol
ΔE_{int} = 2.1 kcal/mol

Atom(A)···Atom(B)	δg^{inter}	Distance (Å)
H46···F109	0.0292	2.402
H63···F104	0.0108	2.830

Figure S45. IGMH plot showing interactions between **[1-NBA]⁺** and a neighbouring [FPB]⁻ anion. Cation and anion are defined as separate fragments; sign(λ_2)ρ–coloured isosurfaces are plotted with δG^{inter} = 0.003 a.u; relative atomic contributions coloured by %δg^{atom}. The molecular structure with selected atom labelling is also shown along with tabulated dg^{inter} data and distances for selected atom pairs, ion pair interaction energies (DE_{int}, PBE-D3) and an estimate of the contribution of dispersion (DE dispersion).

S4.3 Cation-Cation Ion-pair Interactions

Cation-Cation Ion-pair1:

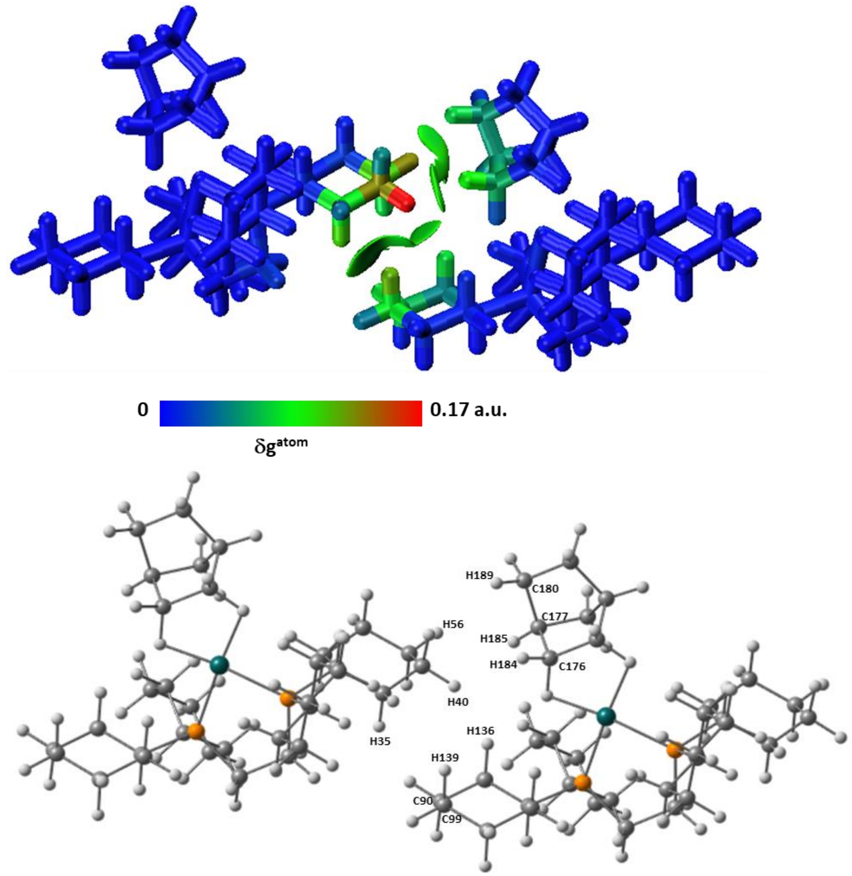

ΔE_{int} = -20.0 kcal/mol

$\Delta E_{dispersion}$ = 7.1 kcal/mol

**Atom(A)···Atom(B)**	**δg^{inter}**	**Distance (Å)**
H40···H184	0.0261	2.169
H35···H139	0.0250	2.345
H56···H189	0.0213	2.397
H40···H136	0.0206	2.486
H35···C99	0.0192	2.959
H56···C180	0.0192	2.936
H56···C177	0.0183	2.958
H40···H139	0.0166	2.568
H40···C176	0.0164	3.103
H56···H185	0.0161	2.523
H35···C90	0.0132	3.163
H56···C176	0.0105	2.958

Figure S46. IGMH plot showing interactions between **[1-NBA]⁺** and a neighbouring [1-NBA]⁺ cation. Cation and anion are defined as separate fragments; sign(λ_2)ρ–coloured isosurfaces are plotted with δG^{inter} = 0.003 a.u; relative atomic contributions coloured by %δg^{atom}. The molecular structure with selected atom labelling is also shown along with tabulated dg^{inter} data and distances for selected atom pairs, ion pair interaction energies (DE_{int}, PBE-D3) and an estimate of the contribution of dispersion (DE dispersion).

Cation-Cation Ion-pair2:

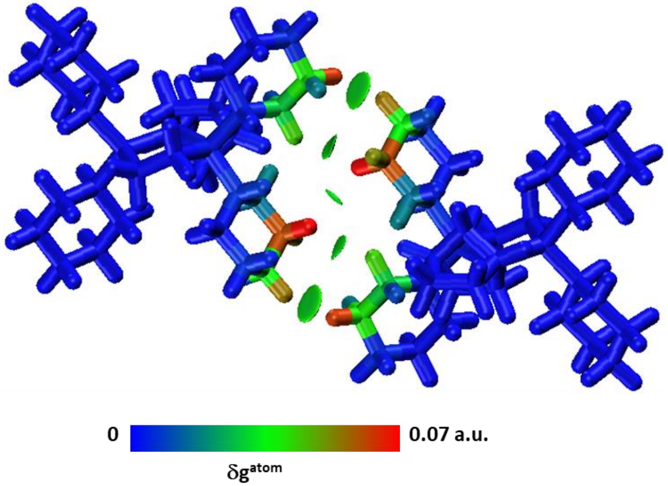

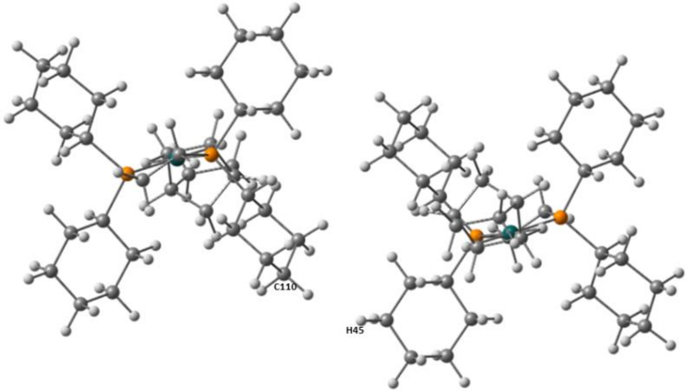

ΔE_{int} = -20.7 kcal/mol
$\Delta E_{dispersion}$ = 5.3 kcal/mol

Atom(A)···Atom(B)	δg^{inter}	Distance (Å)
H45···C110	0.0127	3.191

Figure S47. IGMH plot showing interactions between **[1-NBA]⁺** and a neighbouring [1-NBA]⁺ cation. Each cation is defined as a separate fragment; sign(λ_2)ρ–coloured isosurfaces are plotted with δG^{inter} = 0.003 a.u; relative atomic contributions coloured by %δg^{atom}. The molecular structure with selected atom labelling is also shown along with tabulated dg^{inter} data and distances for selected atom pairs, ion pair interaction energies (DE_{int}, PBE-D3) and an estimate of the contribution of dispersion (DE dispersion).

Cation-Cation Ion-pair3:

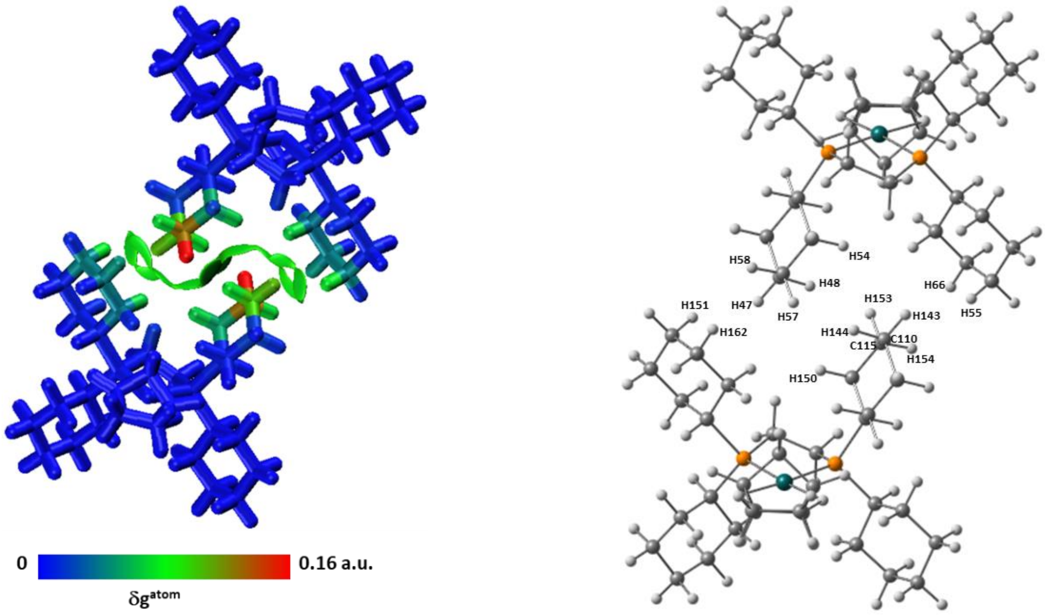

E_{int} = -17.4 kcal/mol
$\Delta E_{dispersion}$ = 9.0 kcal/mol

Atom(A)···Atom(B)	δg^{inter}	Distance (Å)
H54···H153	0.0193	2.529
H57···H150	0.0193	2.529
H47···H151	0.0190	2.405
H55···H143	0.0190	2.405
H57···H144	0.0181	2.456
H48···H153	0.0181	2.456
H57···C115	0.0123	3.233
H58···H162	0.0122	2.618
H66···C154	0.0122	2.618
H55···C110	0.0115	3.206

Figure S48. IGMH plot showing interactions between **[1-NBA]⁺** and a neighbouring [1-NBA]⁺ cation. Cation and anion are defined as separate fragments; sign(λ_2)ρ–coloured isosurfaces are plotted with δG^{inter} = 0.003 a.u; relative atomic contributions coloured by %δg^{atom}. The molecular structure with selected atom labelling is also shown along with tabulated dg^{inter} data and distances for selected atom pairs, ion pair interaction energies (DE_{int}, PBE-D3) and an estimate of the contribution of dispersion (DE dispersion).

Cation-Cation Ion-pair4:

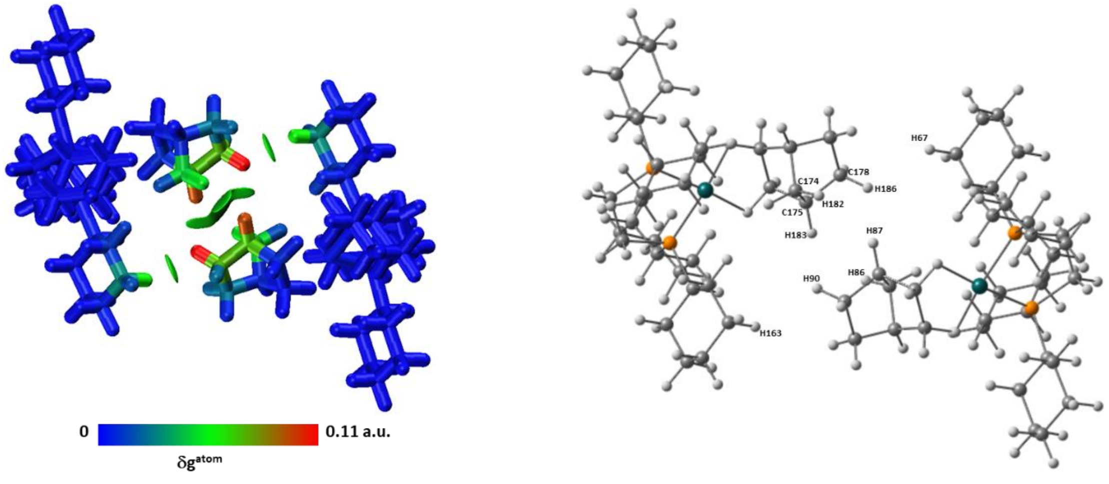

E_{int} = -21.4 kcal/mol
$\Delta E_{dispersion}$ = 6.2 kcal/mol

Atom(A)···Atom(B)	δg^{inter}	Distance (Å)
H90···H183	0.0158	2.494
H67···H186	0.0158	2.494
H87···H186	0.0154	2.564
H90···H183	0.0154	2.564
H87···H182	0.0151	2.497
H86···H183	0.0151	2.497
H87···C175	0.0123	3.211
H67···H178	0.0111	3.198
H87···C174	0.0108	3.229

Figure S49. IGMH plot showing interactions between **[1-NBA]⁺** and a neighbouring [1-NBA]⁺ cation. Cation and anion are defined as separate fragments; sign(λ_2)ρ–coloured isosurfaces are plotted with δG^{inter} = 0.003 a.u; relative atomic contributions coloured by %δg^{atom}. The molecular structure with selected atom labelling is also shown along with tabulated dg^{inter} data and distances for selected atom pairs, ion pair interaction energies (DE_{int}, PBE-D3) and an estimate of the contribution of dispersion (DE dispersion).

Cation-Cation Ion-pair5:

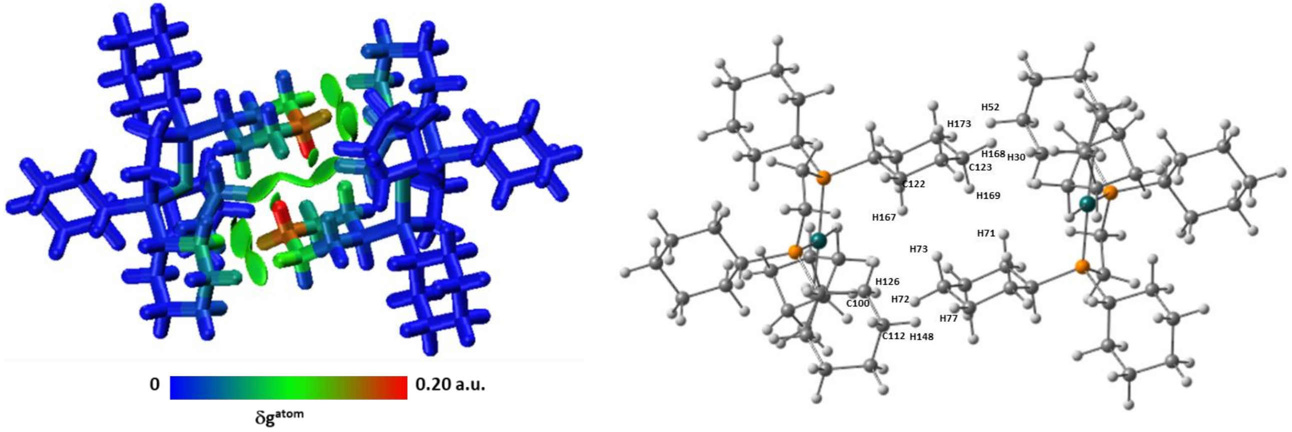

E_{int} = -17.0 kcal/mol
$\Delta E_{dispersion}$ = 12.0 kcal/mol

**Atom(A)···Atom(B)**	**δg^{inter}**	**Distance (Å)**
H73···H167	0.0288	2.199
H71···H169	0.0288	2.199
H30···H168	0.0244	2.339
H72···H126	0.0244	2.339
H77···H148	0.0236	2.199
H52···H173	0.0236	2.199
H73···C169	0.0213	2.526
H30···C123	0.0209	2.928
H77···C112	0.0168	2.985
H71···C123	0.0156	3.236
H73···C122	0.0151	3.177
H77···C100	0.0132	3.182

Figure S50. IGMH plot showing interactions between **[1-NBA]⁺** and a neighbouring [1-NBA]⁺ cation. Cation and anion are defined as separate fragments; sign(λ_2)ρ–coloured isosurfaces are plotted with δG^{inter} = 0.003 a.u; relative atomic contributions coloured by %δg^{atom}. The molecular structure with selected atom labelling is also shown along with tabulated dg^{inter} data and distances for selected atom pairs, ion pair interaction energies (DE_{int}, PBE-D3) and an estimate of the contribution of dispersion (DE dispersion).

S5.0 X-Ray Crystallography

Single-crystal X-Ray diffraction data were collected on a Rigaku SuperNova diffractometer with Cu-Kα (λ = 1.54184 Å) radiation equipped with a nitrogen gas Oxford Cryosystems Cryostream unit or on a Rigaku Synergy HyPix-Arc diffractometer with Cu-Kα (λ = 1.54184 Å) equipped with a nitrogen gas Oxford Cryosystems Cryostream-1000 unit, at the University of York. The crystals were kept at 110.00(10) K during data collection. Diffraction images from raw frame data were reduced using the CrysAlisPro suite of programmes. The structures were solved using SHELXT[20] structure solution program using Intrinsic Phasing and refined with SHELXL2[20] refinement package using least squares minimisation through the Olex2 GUI.[21] All nonhydrogen atoms were refined anisotropically and hydrogen atoms were geometrically placed and allowed to ride on their parent atoms. Distances and angles were calculated using the full covariance matrix. Crystallographic data are available free of charge via the Cambridge Crystallographic Data Centre, under deposition numbers 2434355-2434359

S5.1 Crystallographic Table of Data

Identification code	**Na[FPB]**	**[H(OEt$_2$)][FPB]**	**[1-NBD][FPB]**	**[1-NBA][FPB]**	**[1-F2C$_6$H$_4$][FPB]**
CCDC#	2434355	2434356	2434357	2434358	2434359
Empirical formula	$BC_{12}F_{24}NaO_4$	$C_{16}H_{11}BF_{24}O_5$	$C_{45}H_{56}BO_4F_{24}P_2Rh$	$C_{45}H_{60}BO_4F_{24}P_2Rh$	$C_{50}H_{56}BO_4F_{28}P_2Rh$
Formula weight	697.92	750.06	1292.55	1296.59	1428.60
Temperature/K	110.00(10)	110.00(10)	110.00(10)	110.00(10)	110.00(10)
Crystal system	tetragonal	monoclinic	Triclinic	triclinic	triclinic
Space group	$P4_2/nbc$	$P2_1/c$	$P\bar{1}$	$P\bar{1}$	$P\bar{1}$
a/Å	13.2556(2)	17.2455(2)	11.59080(10)	11.4794(2)	10.63160(10)
b/Å	13.2556(2)	14.7350(2)	14.9216(2)	15.6582(3)	14.98470(10)
c/Å	12.1193(3)	20.8636(3)	16.2883(2)	15.6878(3)	17.87050(10)
α/°	90	90	98.8530(10)	99.748(2)	93.6830(10)
β/°	90	112.234(2)	109.1600(10)	98.188(2)	100.5810(10)
γ/°	90	90	101.4160(10)	107.198(2)	101.7080(10)
Volume/Å^3	2129.49(8)	4907.50(13)	2534.50(5)	2598.78(9)	2725.06(4)
Z	4	8	2	2	2
ρcalcg/cm^3	2.177	2.030	1.694	1.657	1.741
μ/mm^{-1}	2.898	2.439	4.526	4.414	4.388
F(000)	1344.0	2944.0	1308.0	1316.0	1440.0
Crystal size/mm^3	0.08 x 0.03 x 0.02	0.16 × 0.08 × 0.03	0.2 × 0.15 × 0.13	0.1 × 0.08 × 0.06	0.13 × 0.1 × 0.07
Radiation	Cu Kα (λ = 1.54184)	Cu Kα (λ = 1.54184)	Cu Kα (λ = 1.54184)	Cu Kα (λ = 1.54184)	Cu Kα (λ = 1.54184)
2Θ range for data collection/°	9.436 to 157.668	5.536 to 160.842	7.502 to 153.934	5.84 to 160.922	5.058 to 160.46
Index ranges	-16 ≤ h ≤ 11, -16 ≤ k ≤ 16, -5 ≤ l ≤ 14	-22 ≤ h ≤ 21, -18 ≤ k ≤ 17, -26 ≤ l ≤ 26	-13 ≤ h ≤ 14, -18 ≤ k ≤ 17, -20 ≤ l ≤ 20	-14 ≤ h ≤ 14, -19 ≤ k ≤ 20, -17 ≤ l ≤ 19	-13 ≤ h ≤ 13, -19 ≤ k ≤ 19, -22 ≤ l ≤ 19
Reflections collected	5351	48611	48814	29638	53370
Independent reflections	1090 [R_{int} = 0.0222, R_{sigma} = 0.0191]	9991 [R_{int} = 0.0405, R_{sigma} = 0.0281]	10380 [R_{int} = 0.0380, R_{sigma} = 0.0262]	10456 [R_{int} = 0.0308, R_{sigma} = 0.0340]	11271 [R_{int} = 0.0337, R_{sigma} = 0.0249]
Data/restraints/parameters	1090/0/97	9991/0/841	10380/0/694	10456/0/702	11271/0/775
Goodness-of-fit on F^2	1.058	1.080	1.047	1.045	1.059
Final R indexes [I>=2σ (I)]	R_1 = 0.0455, wR_2 = 0.1066	R_1 = 0.0446, wR_2 = 0.1230	R_1 = 0.0270, wR_2 = 0.0706	R_1 = 0.0326, wR_2 = 0.0808	R_1 = 0.0253, wR_2 = 0.0620
Final R indexes [all data]	R_1 = 0.0499, wR_2 = 0.1095	R_1 = 0.0525, wR_2 = 0.1292	R_1 = 0.0278, wR_2 = 0.0712	R_1 = 0.0410, wR_2 = 0.0850	R_1 = 0.0271, wR_2 = 0.0629
Largest diff. peak/hole / e Å^{-3}	0.50/-0.41	0.42/-0.48	0.91/-0.65	0.57/-0.87	0.58/-0.43

S5.2 X-Ray crystallographic Figures

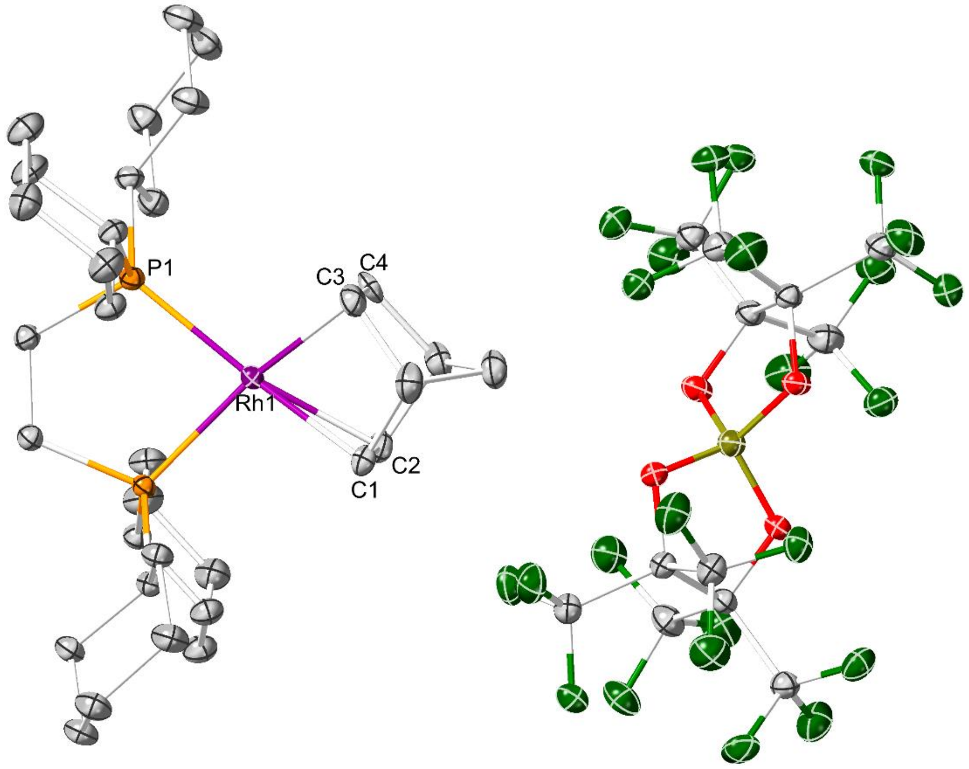

Figure S51. Solid-state molecular structure of complex **[1-NBD][FPB]** (Displacement ellipsoids are set at 50% probability, selected hydrogen atoms have been removed for clarity). Selected bond lengths (Å): Rh1–P1 2.3076(6), Rh1–P2 2.2987(5), Rh1–C1/2 2.1050(12), Rh1–C3/4 2.0941(12). Selected bond angles (°): P1–Rh1–P2: 84.99(2).

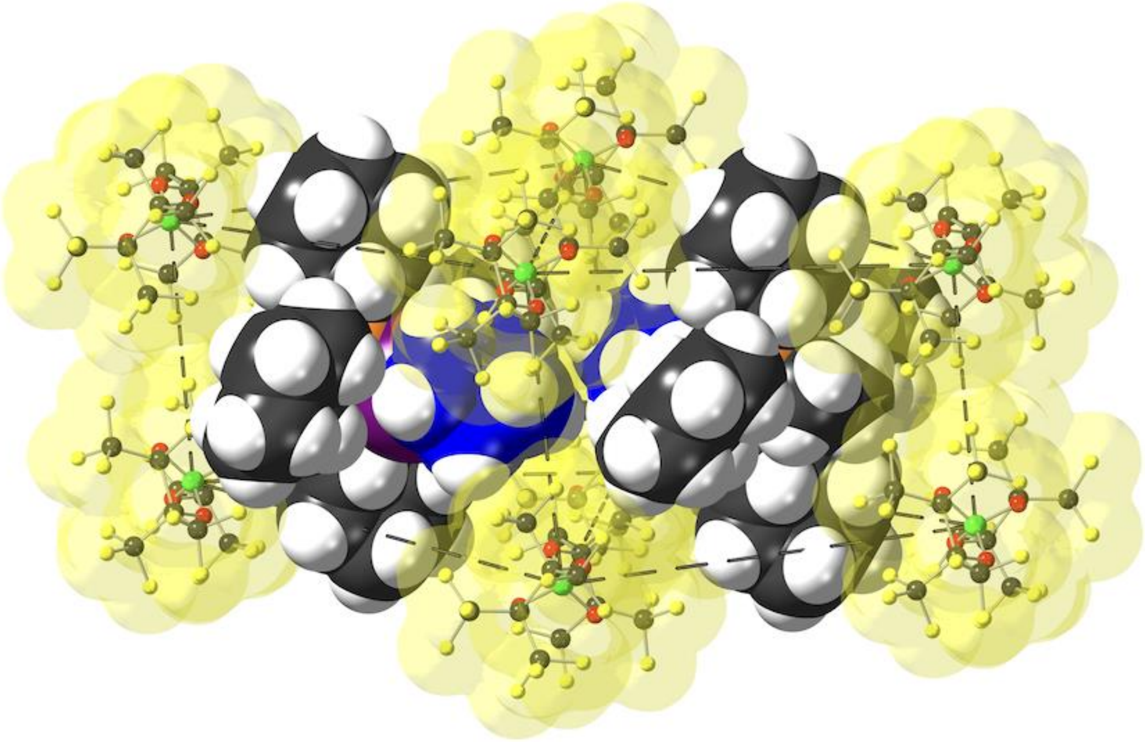

Figure S52. Packing arrangement of complex **[1-NBD][FPB]** showing the norbornadiene ligands directed (blue) towards each other. Van der Waals surfaces.

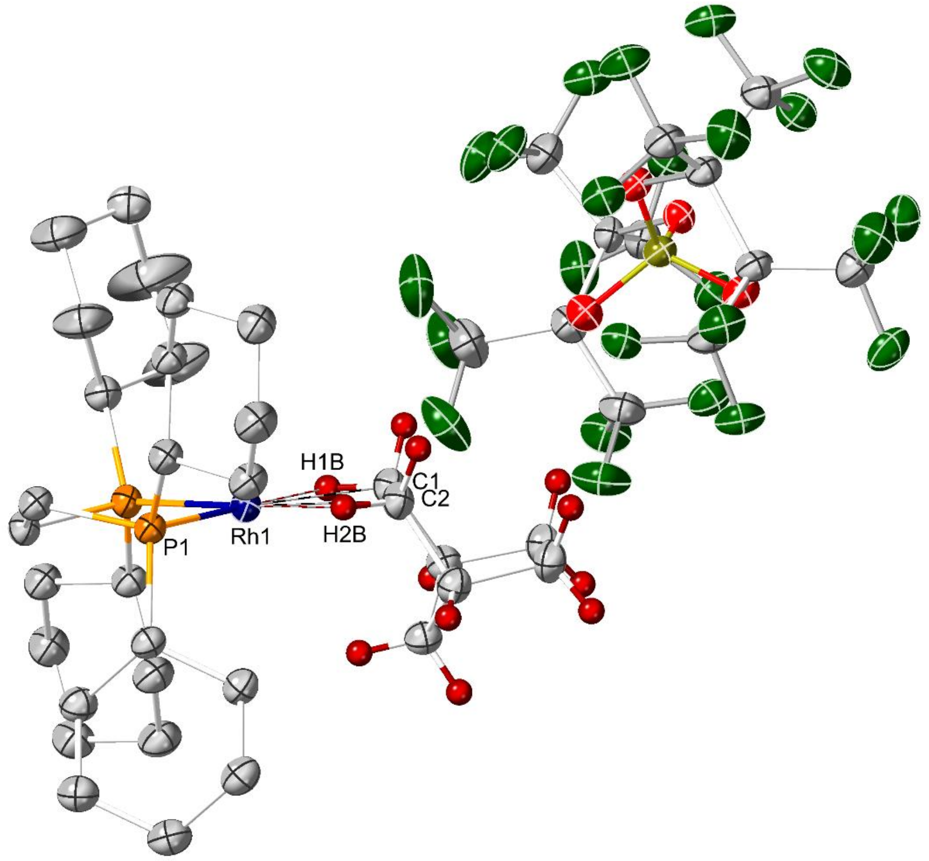

Figure S53. Solid-state molecular structure of complex **[1-NBA][FPB]** (Displacement ellipsoids are set at 50% probability, selected hydrogen atoms have been removed for clarity). Selected bond lengths (Å): Rh1–P1 2.2107(7), Rh1–P2 2.2089(7), Rh1–C1 2.384(3), Rh1–C2 2.363(3). Selected bond angles (°): P1–Rh1–P2 85.19(2), P1–Rh1–C2 117.11(7), P2–Rh1–C1 119.63(7), C1–Rh1–C2 38.07(9),

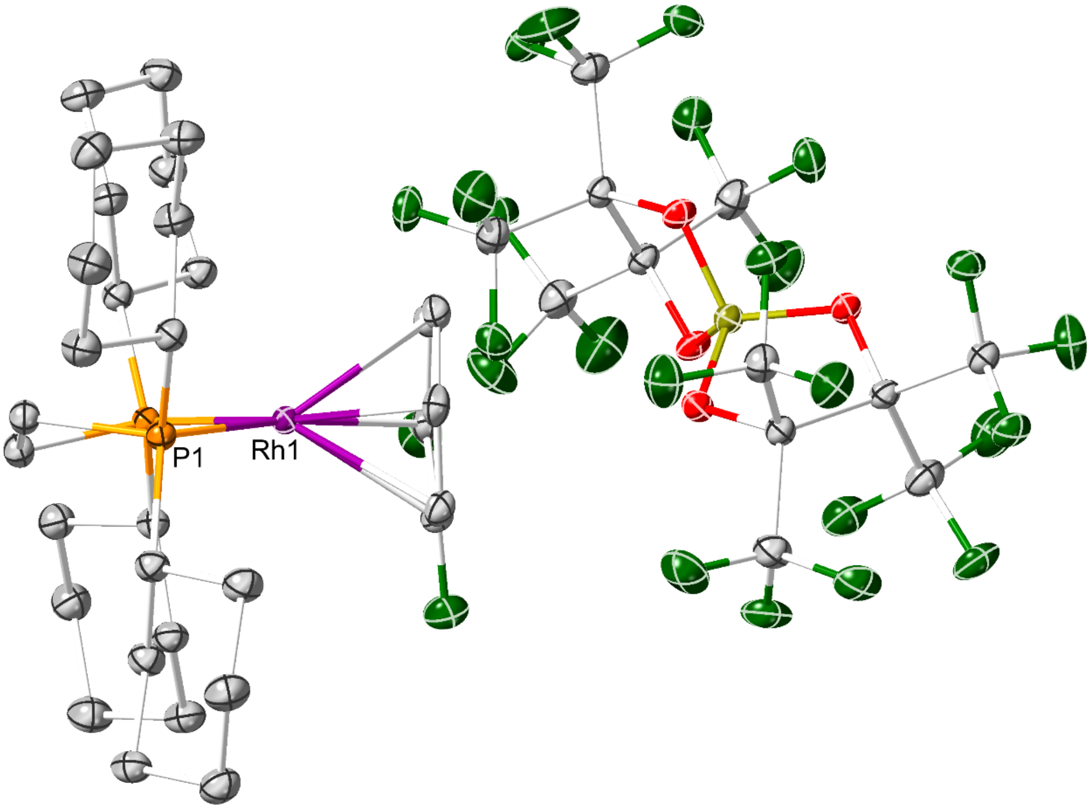

Figure S54. Solid-state molecular structure of complex **[1-$F_2C_6H_4$][FPB]** (Displacement ellipsoids are set at 50% probability, hydrogen atoms and one molecule of lattice 1,2-difluorobenzene have been removed for clarity). Selected bond lengths (Å): P1–Rh1 2.2342(5), P2–Rh1 2.2440(6), Rh1···(1,2-$F_2C_6H_4$) centroid 1.853 Å.

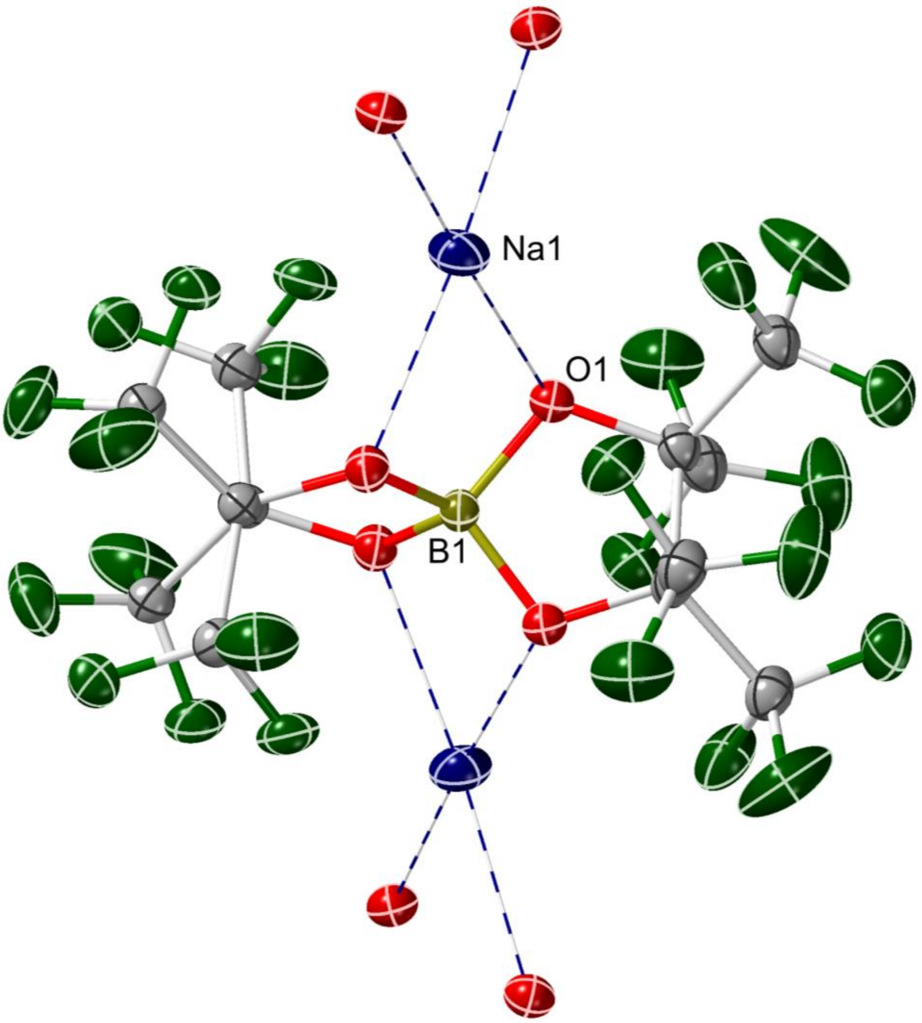

Figure S55. Solid-state molecular structure of **Na[FPB]** (Displacement ellipsoids are set at 50% probability). Selected bond lengths (Å): Na1···O1 2.4432(15).

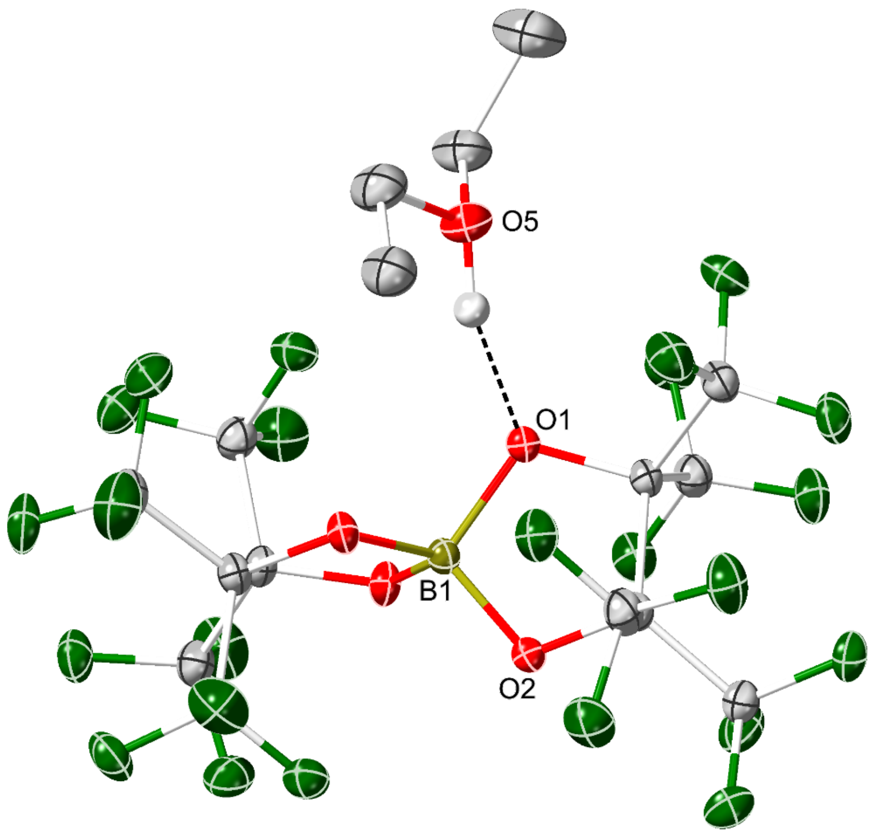

Figure S56. Solid-state molecular structure of **[H(Et$_2$O)][FPB]** (Displacement ellipsoids are set at 50% probability). Selected bond lengths (Å): B1-O1 1.514(3), B1-O2 1.459(2), O1-H5 1.64(3). Selected bond angles (°): O1-B1-O2 102.8(2), O4-B1-O3 104.5(2), B1-O1-H5 123(1), O5-H5-O1 161(3).

S6.0 References

(1) Ni, L.; Zhang, S.; Li, C.; Lu, J.; Li, J.; Wang, J.; Zhang, S.; Chen, G.; Zhang, Z.; Sun, F.; et al. Tailored Cation–Anion Coordination in Carbonate Electrolyte Enabling a Rigid-Flexible Compact Solid-Electrolyte Interphase for Potassium Batteries. *Adv. Funct. Mater*. **2024**, 34, 2400570.

(2) Brookhart, M.; Grant, B.; Volpe, A. F. [(3,5-(CF3)2C6H3)4B]-[H(OEt2)2]+: a convenient reagent for generation and stabilization of cationic, highly electrophilic organometallic complexes. *Organometallics* **2002**, 11, 3920-3922.

(3) Chadwick, F. M.; Kramer, T.; Gutmann, T.; Rees, N. H.; Thompson, A. L.; Edwards, A. J.; Buntkowsky, G.; Macgregor, S. A.; Weller, A. S. Selective C-H Activation at a Molecular Rhodium Sigma-Alkane Complex by Solid/Gas Single-Crystal to Single-Crystal H/D Exchange. J. Am. Chem. Soc. 2016, 138, 13369-13378

(4) VandeVondele, J.; Krack, M.; Mohamed, F.; Parrinello, M.; Chassaing, T.; Hutter, J. Quickstep: Fast and accurate density functional calculations using a mixed Gaussian and plane waves approach. *Comput. Phys. Commun.* **2005**, *167*, 103-128.

(5) Hutter, J.; Iannuzzi, M.; Schiffmann, F.; VandeVondele, J. cp2k: atomistic simulations of condensed matter systems. *Wires Comput. Mol. Sci.* **2014**, *4*, 15-25.

(6) VandeVondele, J.; Hutter, J. Gaussian basis sets for accurate calculations on molecular systems in gas and condensed phases. *J. Chem. Phys.* **2007**, *127*,

(7) Hartwigsen, C.; Goedecker, S.; Hutter, J. Relativistic separable dual-space Gaussian pseudopotentials from H to Rn. *Phys. Rev. B* **1998**, *58*, 3641-3662.

(8) Goedecker, S.; Teter, M.; Hutter, J. Separable dual-space Gaussian pseudopotentials. *Phys. Rev. B* **1996**, *54*, 1703-1710.

(9) Krack, M. Pseudopotentials for H to Kr optimized for gradient-corrected exchange-correlation functionals. *Theor. Chem. Acc.* **2005**, *114*, 145-152.

(10) Perdew, J. P.; Burke, K.; Ernzerhof, M. Generalized Gradient Approximation Made Simple. *Phys. Rev. Lett.* **1996**, *77*, 3865-3868.

(11) Grimme, S.; Antony, J.; Ehrlich, S.; Krieg, H. A consistent and accurate ab initio parametrization of density functional dispersion correction (DFT-D) for the 94 elements H-Pu. *J. Chem. Phys.* **2010**, *132*, 154104.

(12) Lu, T.; Chen, F. Multiwfn: A multifunctional wavefunction analyzer. *J. Comput. Chem.* **2012**, *33*, 580-592.

(13) Lu, T.; Chen, Q. Independent gradient model based on Hirshfeld partition: A new method for visual study of interactions in chemical systems. *J. Comput. Chem.* **2022**, *43*, 539-555.

(14) Humphrey, W.; Dalke, A.; Schulten, K. VMD: Visual molecular dynamics. *Journal of Molecular Graphics* **1996**, *14*, 33-38.

(15) Bader, R. F. W. *Atoms in Molecules: A Quantum Theory*; Clarendon Press, 1994.

(16) *AIMAll (Version 17.11.14)*; Keith, T. A.; TK Gristmill Software: Overland Park KS, USA, 2017.

(17) Johnson, E. R.; Keinan, S.; Mori-Sánchez, P.; Contreras-García, J.; Cohen, A. J.; Yang, W. Revealing Noncovalent Interactions. *J. Am. Chem. Soc.* **2010**, *132*, 6498-6506.

(18) Contreras-García, J.; Johnson, E. R.; Keinan, S.; Chaudret, R.; Piquemal, J.-P.; Beratan, D. N.; Yang, W. NCIPLOT: A Program for Plotting Noncovalent Interaction Regions. *J. Chem. Theory Comput.* **2011**, *7*, 625-632.

(19) *NBO 6.0*; Glendening, E. D.; Badenhoop, J. K.; Reed, A. E.; Carpenter, J. E.; Bohmann, J. A.; Morales, C. M.; Landis, C. R.; Weinhold, F.; Theoretical Chemistry Institute, University of Wisconsin, Madison, WI, 2013.

(20) Sheldrick, G. M. SHELXT - integrated space-group and crystal-structure determination. *Acta Cryst*. **2015**, C71, 3-8.

(21) Dolomanov, O. V.; Bourhis, L. J.; Gildea, R. J.; Howard, J. A. K.; Puschmann, H. OLEX2: a complete structure solution, refinement and analysis program. *J. Appl. Cryst*. **2009**, 42, 339-341.
